# Crystallization-Driven Quadrant-Specific Spherulitic Self-Assembly in Partially Miscible Biodegradable PBS/PCL/PBS-*ran*-PCL Blends

**DOI:** 10.1021/jacs.5c22442

**Published:** 2026-03-30

**Authors:** Maryam Safari, Roy Kneepkens, Ricardo A. Pérez-Camargo, Agurtzane Mugica, Manuela Zubitur, Manfred Burghammer, Guoming Liu, Dujin Wang, Jules A.W. Harings, Alejandro J. Müller

**Affiliations:** † 5211 Maastricht University-Aachen Maastricht Institute for Biobased Materials (AMIBM), Urmonderbaan 22, Geleen 6167 RD, The Netherlands; ‡ POLYMAT and Department of Polymers and Advanced Materials: Physics, Chemistry and Technology, Faculty of Chemistry, 160665 University of the Basque Country UPV/EHU, Paseo Manuel de Lardizabal 3, Donostia-San Sebastián 20018, Spain; § Chemical and Environmental Engineering Department, Polytechnic School, 16402 University of the Basque Country UPV/EHU, Plaza Europa 1, Donostia-San Sebastián 20018, Spain; ∥ 55553 European Synchrotron Radiation Facility, 6 rue Jules Horowitz, BP220, Grenoble 38043 cedex 9, France; ⊥ Beijing National Laboratory for Molecular Sciences, CAS Key Laboratory of Engineering Plastics, Institute of Chemistry, 12381 Chinese Academy of Sciences, Beijing 100190, China; # University of Chinese Academy of Sciences, Beijing 100049, China; ∇ IKERBASQUE, Basque Foundation for Science, Plaza Euskadi 5, Bilbao 48009, Spain

## Abstract

The inherent immiscibility of biodegradable aliphatic polyesters, such as poly­(butylene succinate) (PBS) and poly­(ε-caprolactone) (PCL), hampers the development of strong yet degradable plastics. Here, we demonstrate that random isodimorphic copolyesters, poly­(butylene succinate-*ran*-ε-caprolactone) (BS_*x*_CL_*y*_), effectively compatibilize equimolar PBS/PCL blends through a matrix-driven crystallization mechanism that couples the two phases at the molecular level. Multiscale characterization, combining DSC, *in situ* and spatially resolved polarized FT-IR imaging, and nanobeam synchrotron WAXD/SAXS, reveals the first structural evidence of quadrant-specific spherulites, in which alternating quadrants exhibit distinct lamellar architectures: banded regions with continuous twisting and nonbanded regions with uniform orientation. This pronounced morphological anisotropy arises from the selective cocrystallization of BS-rich segments (within the random copolymer) with the PBS blend component and the formation of PBS β-form crystals with looser molecular packing. These features promote interfacial coupling and enhance degradability. The concept of matrix-directed crystallization establishes a potentially general framework for compatibilizing immiscible biodegradable polyesters and for designing biobased plastics with tunable crystalline hierarchy, mechanical performance, and controlled biodegradation behavior.

## Graphical abstract

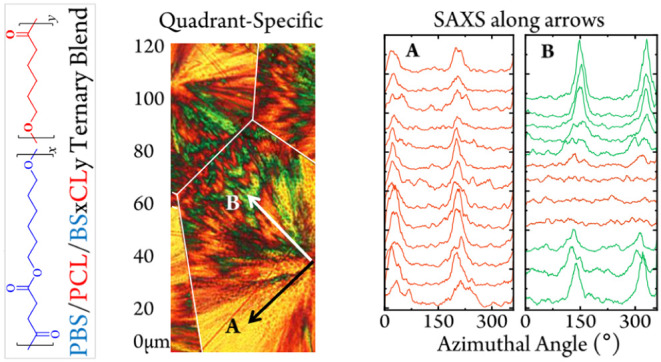

## Introduction

The growing demand for ecofriendly plastics has boosted interest in biobased and biodegradable aliphatic polyesters. Among them, poly­(butylene succinate) (PBS) combines partial biobased origin with mechanical strength comparable to polyolefins,^1,2^ while poly­(*ε*-caprolactone) (PCL) is soft and ductile (*T*_g_ ≈ −60 °C). The biodegradation of these polyesters depends on their degree of crystallinity and the degradation environment (hydrolytic versus enzymatic degradation).^3^ Blending PBS with PCL hypothetically offers a sustainable strategy to balance stiffness, flexibility, and degradability. However, their immiscibility in all states leads to phase separation, weak interfacial adhesion, and poor mechanical integrity.^1,4–9^

Compatibilization using copolymers that share structural motifs with both components is a common strategy to overcome this limitation.^10–14^ Random PBS-*ran*-PCL copolyesters (BS_*x*_CL_*y*_) are interesting materials; they exhibit isodimorphic crystallization^5,15–22^ where each crystalline phase partially incorporates the comonomer, enabling tunable thermal and mechanical properties.^5,20^ PBS/PCL blends are immiscible and experience uncontrolled crystallization and limited phase integration.^5,9^ Recent studies reveal that the crystalline phase adopted by BS_*x*_CL_*y*_ depends on the surrounding matrix, suggesting a promising route to compatibilize immiscible polyester blends through matrix-driven crystallization.^5^

Here, we explore the compatibilization of equimolar PBS/PCL blends using isodimorphic random BS_*x*_CL_*y*_ copolyesters with compositions spanning both sides of the pseudoeutectic point. In the SI (Section S3), we explain what an isodimorphic random copolymer is and how the first-order crystallization and melting transitions depend on composition, displaying a pseudoeutectic point. By combining multiscale structural and thermal characterization—from differential scanning calorimetry (DSC) and polarized light optical microscopy (PLOM) to *in situ* Fourier transform infrared (FT-IR) and polarized FT-IR (PFT-IR) mapping—together with WAXD/SAXS using an ∼70 nm diameter synchrotron X-ray beam, we achieve the first direct molecular-level elucidation of an unusual “quadrant-specific” spherulitic morphology. As the observed spherulites are not simply divided into two identical halves but exhibit quadrant-dependent optical contrast, with alternating banded and nonbanded regions. The quadrant-specific term reflects their anisotropic yet symmetric sectorial organization rather than a mirror-like two-face morphology—a feature previously observed only optically but never structurally resolved.

Our results show that isodimorphic compatibilizers enhance miscibility (as evidenced by *T*_g_ shifts and merged DSC transitions) and cocrystallize with the PBS blend component, leading to the formation of quadrant-specific spherulites. BS-rich copolymers improve cocrystallization and compatibilization, while CL-rich ones are less effective. These structural effects directly tune crystallinity, interphase structure, and ultimately biodegradation behavior.

This work establishes a matrix-driven crystallization strategy to bridge the gap between well-controlled random copolyester properties and the complex microstructure of practical polyester blends. Beyond PBS/PCL, the approach—combining isodimorphic compatibilizers with real-space, nanometer-resolved structural mapping—provides a general framework to design sustainable biobased plastics with tunable performance and controlled end-of-life degradation.

## Results and Discussion

Figure 1a shows the route leading to BS_*x*_CL_*y*_ random copolyesters using the TTP catalyst.^15,20^ Figure 1b and 1c, and Figure S1 show the nonisothermal cooling and second-heating DSC curves of neat PBS, neat PCL, their 50/50 blend, and the 50/50/10 PBS/PCL/BS_*x*_CL_*y*_ ternary blends. Moreover, in Figure 1b,c, the faded lines represent the theoretical sum curves obtained by mathematically combining the individual components. As can be seen in Figure S1, PBS crystallizes and melts at temperatures higher than those of PCL. The binary 50/50 PBS/PCL blend displays two distinct glass transitions (*T*_g_,_PBS_ ≈ −31 °C and *T*_g_,_PCL_ ≈ −63 °C) and independent crystallization and melting events that mirror those of the homopolymers, indicating that the blend is indeed immiscible.

**Figure 1:**
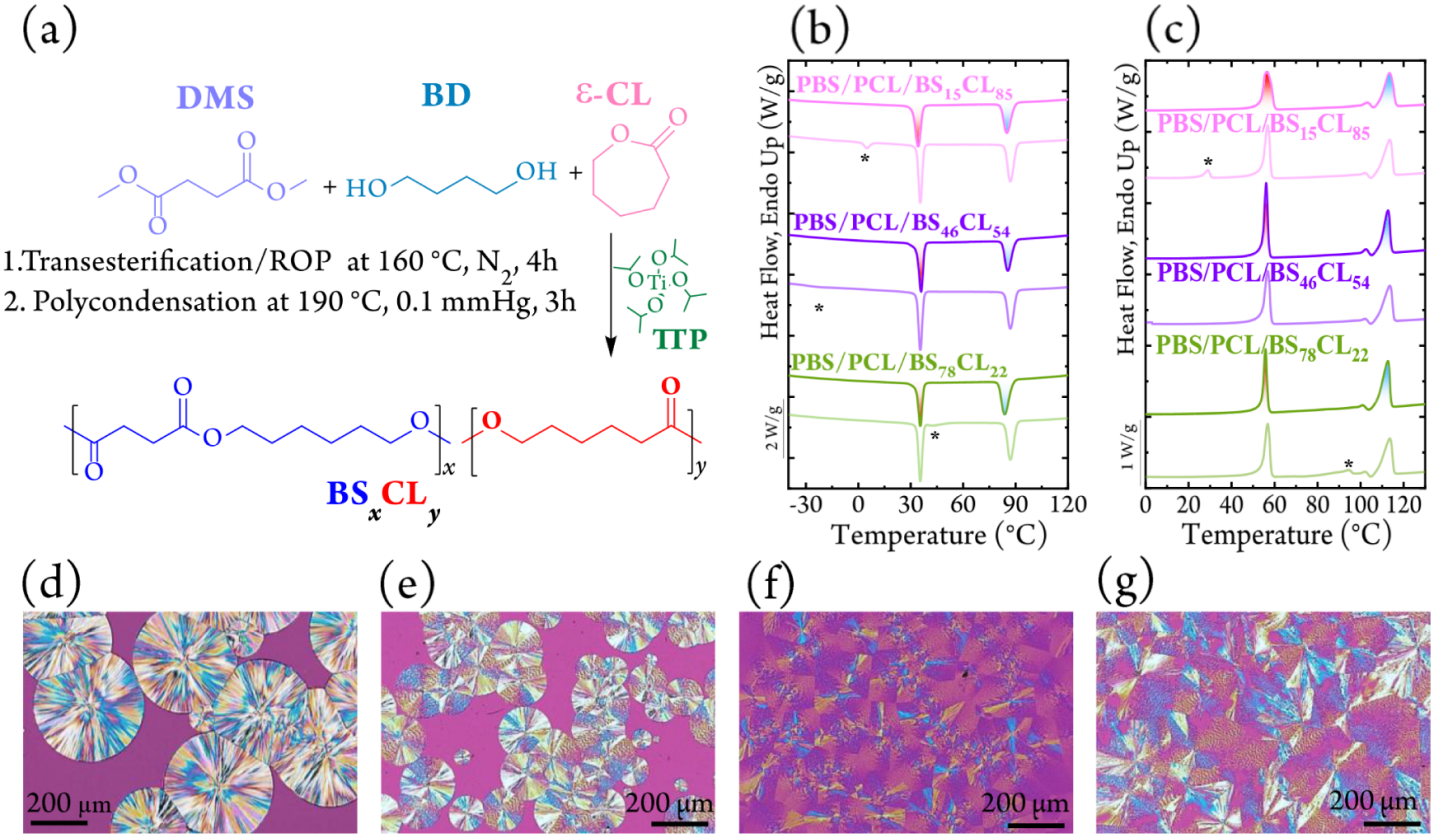
(a) Synthetic route for the preparation of BS*_x_* CL*_y_* random copolymers, (b) DSC scans during cooling, and (c) second heating scans for the indicated samples. PLOM micrographs for neat PBS (d), PBS/PCL/BS_78_CL_22_ (e), PBS/PCL/BS_46_CL_54_ (f), and PBS/PCL/BS_15_CL_85_ (g) samples. In parts (a) and (b), the asterisk denotes the thermal transition position of the BS*_x_* CL*_y_* copolymers in the theoretical blend (i.e., a mathematical superposition of the DSC scans of the homopolymers, scaled by their composition).

Adding 10 wt % of the isodimorphic compatibilizers BS_*x*_CL_*y*_ noticeably modifies this thermal response. The *T*_g_ of both phases shifts toward each other: the *T*_g,PBS_ moves slightly to lower temperatures, while the *T*_g,PCL_ increases, signaling partial miscibility and interphase mixing (see Figure S3).

In Figure 1b,c, the asterisks in the sum curves mark the thermal transitions originating from the BS_*x*_CL_*y*_ copolymers in the blends. Although the neat BS_*x*_CL_*y*_ copolymers exhibit two separate crystallization and melting transitions, these transitions vanish in the blends, implying that the copolymer remains amorphous or cocrystallizes with one of the homopolymers.

The effect is strongly composition-dependent; BS-rich compatibilizers (e.g., BS_78_CL_22_) enhance the PBS phase component crystallization (from Δ*H*_*c*_ = 28 to 35 J/g) and slightly raise PCL’s crystallization temperature (from *T*_*c*_ = 35 to 36 °C), suggesting PBS lamellae act as nucleating sites for PCL lamellae. The pseudoeutectic BS_46_CL_54_ composition shows intermediate behavior, while CL-rich copolymers (e.g., BS_15_CL_85_) interact weakly, leaving transitions similar to those of the immiscible blend.

These DSC results provide clear evidence that isodimorphic compatibilizers can promote partial miscibility and direct crystalline phase selection in equimolar PBS/PCL blends. Reduced comonomer exclusion on the BS-rich side enhances cocrystallization and interfacial adhesion, enabling quadrant-specific spherulites, as revealed by polarized light optical microscopy (PLOM) and later confirmed by *in situ* FT-IR/PFT-IR and nanobeam WAXD/SAXS. In contrast, CL-rich compositions remain less effective and retain an immiscible thermal profile.

PLOM images (Figure 1d–g) illustrate how the isodimorphic compatibilizers control spherulitic morphology during nonisothermal crystallization. Neat PBS forms the expected large, nonbanded, radially growing negative spherulites, while neat PCL forms smaller, nonbanded negative spherulites (see Figure S5). The 50/50 PBS/PCL binary blend exhibits phase-separated morphology: coarse PBS-banded spherulites form first, followed by delayed PCL crystallization confined to the interspherulitic regions (Figure S8).

Adding 10 wt % of the isodimorphic compatibilizers BS_*x*_CL_*y*_ markedly modifies this optical behavior. All compatibilizer compositions yield quadrant-specific spherulites, with two quadrants showing ring-banded extinction and the other two remaining nonbanded.

The nucleation density and band definition depend strongly on composition: BS-rich compatibilizers (e.g., BS_78_CL_22_) produce more nuclei and well-defined bands, while CL-rich ones (e.g., BS_15_CL_85_) generate fewer nuclei and broader, less distinct bands. This quadrant-specific morphology arises from heterogeneous lamellar twisting stabilized by the copolymer, with the nucleation density and band sharpness controlled by composition.

Woo et al.^23^ previously reported Janus-like spherulites of PLLA comprising fast-growing *α′-*form and slower α-form lamellae. In contrast, in the present ternary systems, both banded and nonbanded quadrants grow at identical rates, preserving a perfectly circular growth front throughout crystallization. In addition, Mandala et al.^24^ showed that Janus-like spherulites in PBS/PEO blends form at intermediate compositions and require a top-free surface for quadrant-specific development. In contrast, all ternary samples studied in this contribution displayed identical dual-face morphologies with or without a top glass covering the sample, indicating that film thickness and possible surface effects (due to the glass or air surface) have no impact on our quadrant-specific spherulitic morphologies.

Together, the PLOM and DSC data show that BS-rich compatibilizers promote dense nucleation and stabilize lamellar twisting, producing well-defined quadrant-specific spherulites. CL-rich systems still induce quadrant-specific morphologies, but with lower nucleation density and poorer band contrast (Figure 1). The emergence of these quadrant-specific spherulites provides a clear optical signature of compatibilization and matrix-directed crystallization, anticipating the molecular-level differences explored in the following FT-IR analyses.

*In-situ* FT-IR microscopy was employed to probe the conformational contrast between the banded and nonbanded quadrants of quadrant-specific PBS spherulites^24–27^ in the ternary blend, an approach previously unreported in the literature. By targeting specific regions under the microscope, FT-IR spectra revealed clear differences between the nonbanded (marked A in Figure 2a) and banded (marked B in Figure 2a) areas within a PBS/PCL/BS_78_CL_22_ ternary blend spherulite. Variations in peak intensities and positions, particularly within the C–H stretching region, reflect differences in molecular packing, crystalline vs amorphous content, and molecular conformation. Further details are provided in Supporting Information Section S2.5.

**Figure 2:**
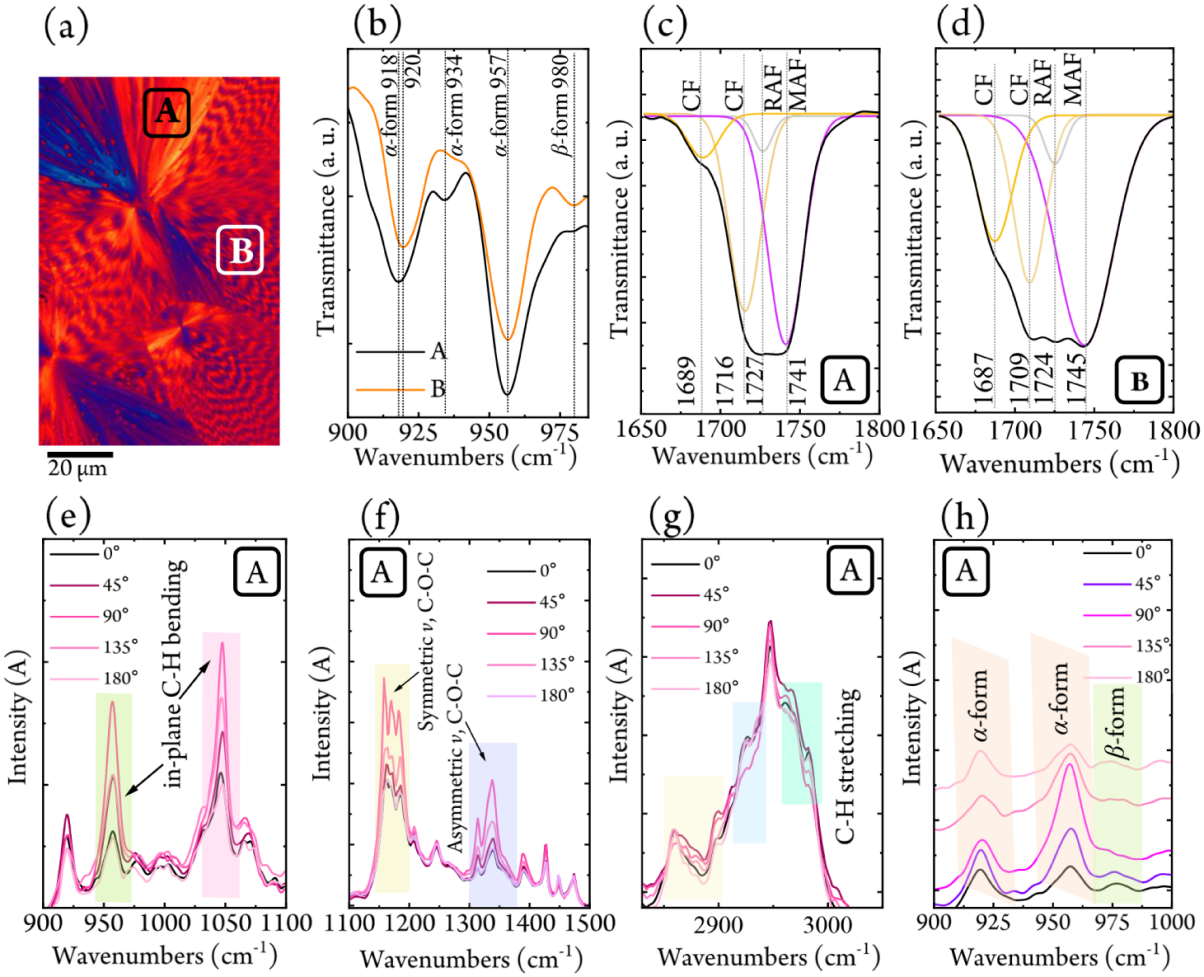
(a) PLOM micrograph for the PBS/PCL/BS_78_CL_22_ sample at 85 °C. A and B indicate the regions where FT-IR spectra were measured within a 20 × 20 μm^2^ area at 85 °C. FT-IR spectra at (b) 900–1000 cm^–1^, (c) 1650–1800 cm^–1^ for the nonbanded part, and (d) for the banded part of a PBS/PCL/BS_78_CL_22_ sample. The dotted lines correspond to the experimental results, and the solid lines to the convoluted curves. PFT-IR spectra for the nonbanded part of PBS/PCL/BS_78_CL_22_ spherulite at (e) 900–1100 cm^–1^, (f) 1100–1500 cm^–1^, (g) 2800–3050 cm^–1^, and (h) 910–990 cm^–1^ at 85 °C.

In Figure 2b, the FT-IR peaks at 918/920, 934, and 955/957 cm^–1^ correspond with the α-form conformation of PBS, while the peak at 979 cm^–1^ is characteristic of the β-form.^28,29^ The coexistence of these peaks in both the A and B regions confirms the PBS polymorphism. The higher intensity of the β-form in the B region indicates its predominance within the banded quadrants, consistent with the β-phase developing under localized stress or deformation, a characteristic feature of PBS.^30,31^

In the nonbanded region (Figure 2c), four C=O stretching bands were identified: 1689 and 1716 cm^–1^ for crystalline fractions (CF); 1727 cm^–1^ for the rigid amorphous fraction (RAF); and 1741 cm^–1^ for the mobile amorphous fraction (MAF).^32–35^ In the banded region (Figure 2d), these bands shift slightly, with CF and RAF to lower wavenumbers (1687, 1709, and 1724 cm^–1^) and MAF to higher wavenumbers (1745 cm^–1^). The red-shift of CF and RAF peaks suggests an increased proportion of β-form crystals, whose lattice parameters (*a* = 0.584, *b* = 0.832, *c* = 1.186 nm and β ≈ 124–125°)^29,31,36^ reflect a less compact arrangement than the α-form crystal (*a* = 0.523, *b* = 0.912, *c* = 1.090 nm, and β ≈ 123°).^29,31,36^ This looser packing weakens intermolecular interactions, resulting in lower-energy vibrational modes. Conversely, the blue-shift of the MAF peak points to a more dynamic, less constrained amorphous environment, consistent with enhanced chain mobility in the banded morphology.

Deconvolution analysis in Figure 2c and d shows that while the relative fractions of amorphous and crystalline domains remain nearly constant (∼4% RAF and ∼46% MAF), the crystalline domains undergo pronounced structural changes between the A and B regions. The CF peak at 1689 cm^–1^ likely corresponds to the β-form, whereas the one at 1716 cm^–1^ is attributed to the α-form, in line with the greater β-phase content observed in the banded regions (Figure 2b).

Figure 2e–g displays the PFT-IR spectra of the nonbanded (A) PBS/PCL/BS_78_CL_22_ region at 85 °C, recorded at various polarizer angles (0° to 180° relative to the glass substrate). The sample was first isothermally crystallized from the melt at 85 °C and maintained at this temperature for 20 min to allow PBS spherulitic growth, after which the FT-IR measurements were performed at 85 °C. At 85 °C, only PBS crystals are present, together with amorphous regions formed by PBS chains and molten PCL chains (PCL crystals melt at temperatures lower than 60 °C; see Figure 1). Pronounced intensity changes (Figure 2e–g) are observed for the in-plane C–H bending, symmetric and asymmetric C–O–C stretching, and C–H stretching vibrations. The C–H bending and C–O–C modes reach maximum intensity at 90°, whereas the C–H stretching band nearly vanishes at the same angle. In contrast, spectra from the banded regions (Figure S17b–d) show no angular dependence, indicating distinct molecular orientations compared to the nonbanded areas.

These observations suggest that in the nonbanded regions, lamellae are predominantly vertical and uniformly aligned, whereas in the banded regions, the lamellae alternate periodically between vertical and horizontal orientations.^24^ This alternation averages out the molecular orientation, explaining the minimal angular variation in the PFT-IR response of the banded areas. The findings are fully consistent with nanobeam X-ray diffraction results, which directly visualize contiguous lamellar twisting and alternating crystal orientations across the banded quadrants, as discussed in the following paragraph.

Figure 2h further illustrates the angular dependence of the α- and β-form crystals.^29^ The β-form peak reaches its maximum intensity at 0° and 180°, while the α-form peak dominates at 90°.

The distinct responses arise from the different monoclinic unit-cell parameters of the two polymorphs: the β-form (β = 123.9°) aligns chains more parallel to the substrate, whereas the α-form (β = 131.6°) adopts a greater chain tilt. Consequently, the β-form peaks at 0°/180°, while the α-form resonates most strongly at 90°, consistent with their respective chain orientations.

*In-situ* simultaneous WAXD/SAXS measurements were performed on all samples during cooling from the melt and subsequent heating at 20 °C/min. Detailed analyses for each homopolymer, binary blend, and ternary blend are provided in the Supplementary Section, S2.6.

Figure 3a,b compares the WAXD and SAXS profiles at −40 °C for the 50/50 PBS/PCL blend and two extreme ternary compositions: PBS/PCL/BS_78_CL_22_ and PBS/PCL/BS_15_CL_85_. In the WAXD profiles (Figure 3a), adding the copolymer induces a slight shift of the reflections toward lower *q* (higher *d*-spacing) values relative to those of the binary blend. The BS_78_CL_22_ additive reduces the intensity of PCL-related peaks while enhancing those of PBS, suggesting that the crystallization of the BS-rich fraction is promoted by PBS crystallization, which in turn restricts the crystallization of the PCL phase. In contrast, BS_15_CL_85_ produces negligible changes in peak areas, indicating limited compatibilization and lower miscibility.

**Figure 3:**
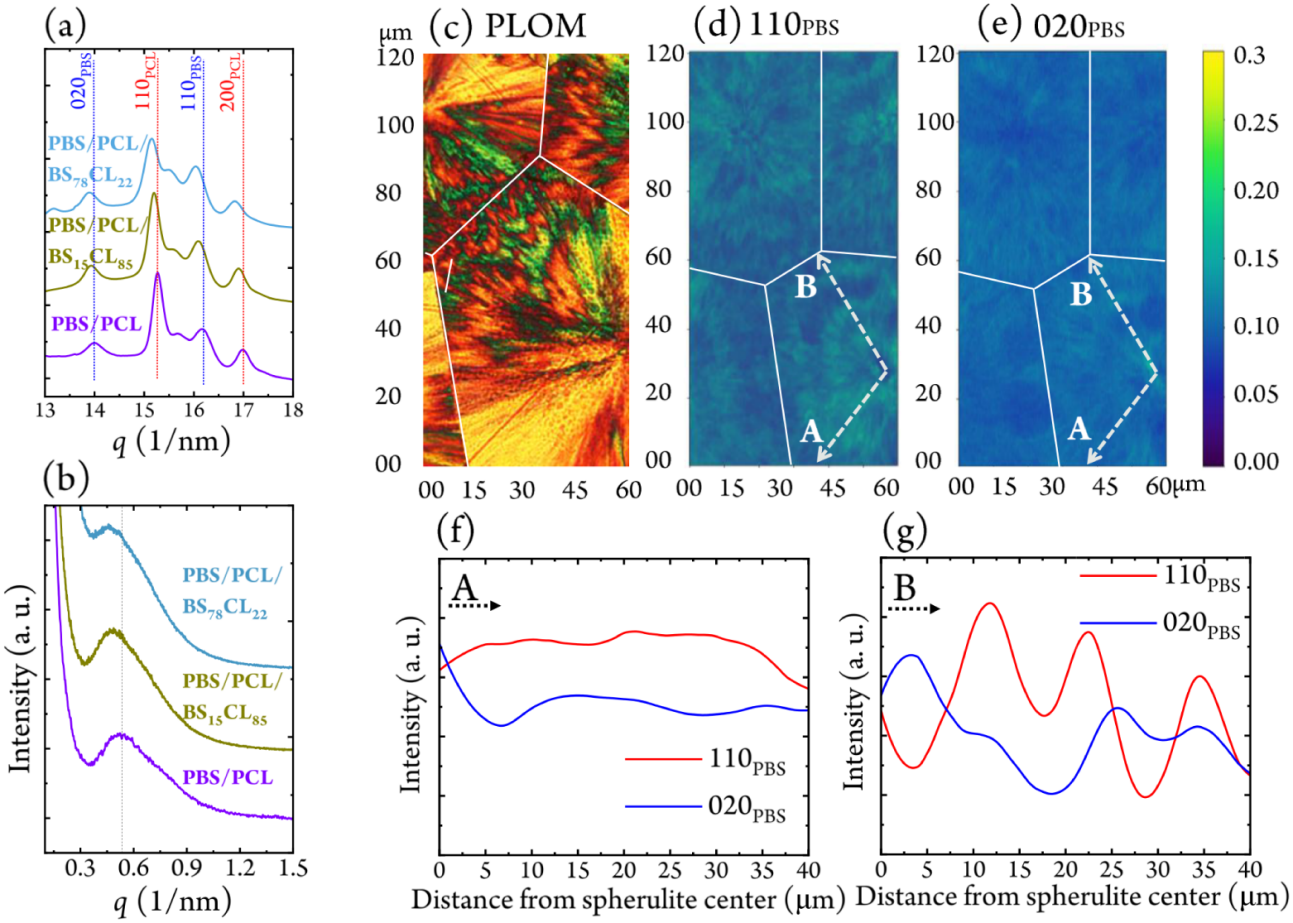
SAXS (a) and WAXD (b) of PBS/PCL binary, PBS/PCL/BS_15_CL_85_ ternary, and PBS/PCL/BS_78_CL_22_ ternary blends at −40 °C. (c) PLOM micrograph of the PBS/PCL/BS_78_CL_22_ ternary blend showing a representative quadrant-specific spherulite sample used for nanobeam X-ray measurements. Intensity map of the 110_PBS_ (d) and 020_PBS_ reflections (e). The solid white lines in parts (c–e) are drawn to guide the eye, indicating the borders between spherulites where they impinged. The extracted intensities of the 110_PBS_ and 020_PBS_ reflections, along dashed arrows labeled A for the nonbanded region (f) and B for the banded region (g), are plotted as a function of the radial distance from the spherulite center.

The SAXS profiles (Figure 3b and see S2.6) show a similar composition-dependent response. Both ternary blends exhibit a small shift toward lower *q* values, corresponding to longer periods (d*), relative to the binary blend. The increase is more pronounced for PBS/PCL/BS_78_CL_22_, with d* = 13.7 nm compared to 12.0 nm for the binary blend, while that of PBS/PCL/BS_15_CL_85_ reaches 12.7 nm. The larger d* in the BS_78_CL_22_ system implies that the copolymer’s amorphous segments insert between PBS lamellae, consistent with the ring-banded morphology observed by PLOM (Figure 1). Conversely, the smaller increase in the level of BS_15_CL_85_ reflects poorer interlamellar penetration and enhanced phase separation.

During cooling, SAXS patterns reveal that PBS crystallizes first at higher *q*, followed by PCL crystallization near 25 °C at lower *q*. In the PBS/PCL/BS_15_CL_85_ blend, two distinct maxima are visible, reflecting its lower miscibility. For PBS/PCL/BS_78_CL_22_, the PBS-related signal appears broadened, indicating stronger interphase interactions, likely due to partial cocrystallization promoted by the copolymer. As the temperature falls below 25 °C, the double maxima merge into a single peak, evidencing progressive phase coupling during crystallization. From the oscillatory SAXS intensity variations observed in the banded quadrants, an approximate lamellar twist periodicity of 36°/μm has been estimated, which agrees with the observations made by PLOM.

We investigated how lamellae self-assemble into quadrant-specific PBS spherulites from the nucleation center to the outer edge. Using nanoscale X-ray beams (70 nm, applied here for the first time in the context of quadrant-specific spherulites),^37^ we directly imaged banded and nonbanded regions within individual spherulites. Simultaneous WAXD and SAXS measurements were collected at multiple radial positions with submicrometer spatial resolution. Figure 3c shows a PLOM image of the PBS/PCL/BS_78_CL_22_ blend prepared for nanobeam X-ray mapping. Each spherulite exhibits quadrant-specific morphology. Intensity maps of the 110_PBS_ and 020_PBS_ reflections are shown in Figure 3d and e, while Figure 3f–g displays the corresponding intensity profiles extracted along the dashed arrows (A, nonbanded; B, banded), from the spherulite center to its edge.

Although the overall WAXD patterns remain consistent across radial positions, the relative intensities of the reflections vary periodically depending on the probed region. In the nonbanded area (Figure 3f), intensity remains nearly constant, indicating uniform lamellar orientation. In contrast, in the banded area (Figure 3g), the intensity oscillates with distance, displaying a repeating wave-like modulation with a period of ∼12 μm. This periodicity reveals continuous lamellar twisting^37–39^ across the banded quadrants.

Nanobeam SAXS analyses further confirmed these structural variations (Figure 4). The SAXS intensity maps (Figure 4a,b, *q* = 0–2.5 nm^–1^) highlight distinct scattering orientations in banded versus nonbanded regions. Spectra were extracted along ∼20 μm paths with 1 μm spacing from areas near the spherulite nucleus. In the nonbanded region (Figure 4c), maxima occur consistently at azimuthal angles of 30° and 210°, suggesting lamellae are uniformly oriented parallel to the X-ray beam. In contrast, the banded region (Figure 4d) shows maxima around 140° and 320°, whose intensity varies periodically (strong at B1/B5, weak at B3/B7 locations), consistent with alternating lamellar orientations, similar to observations in banded PEA spherulites.^39^

**Figure 4:**
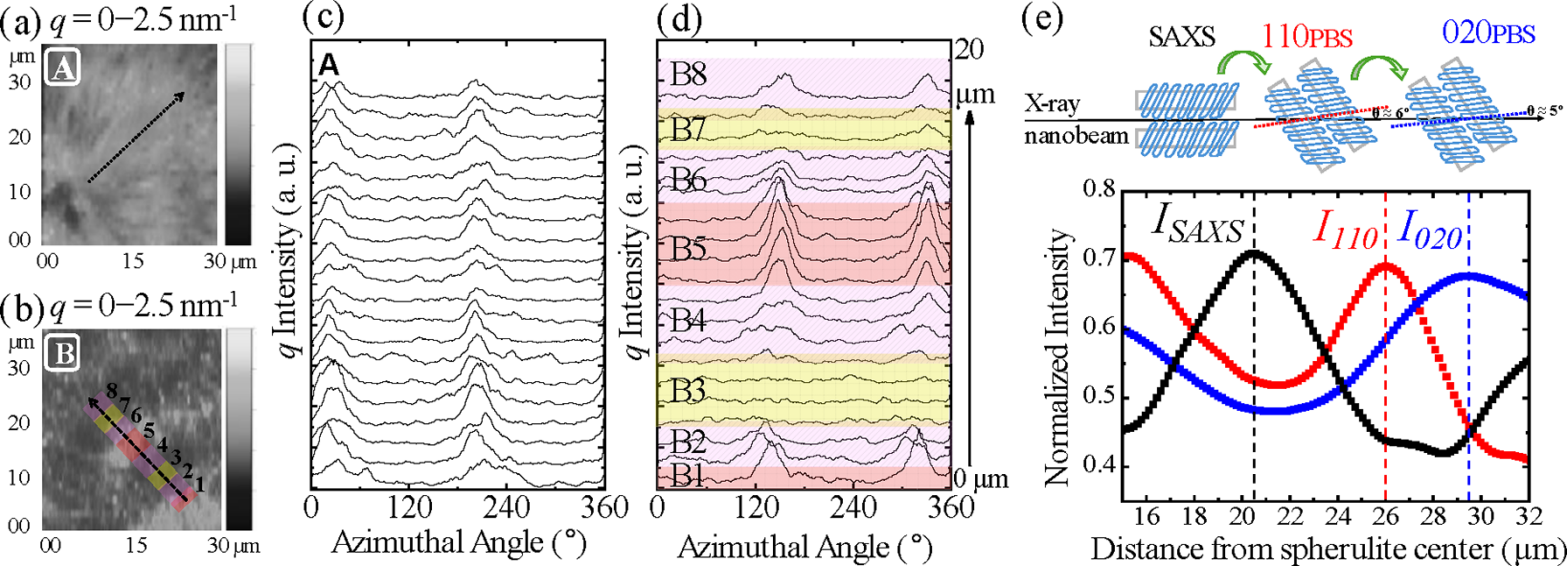
Intensity map of SAXS (*q* = 0–2.5 nm^–1^) in the nonbanded (a) and banded region (b). The extracted intensities of SAXS along dashed arrows for the nonbanded region (c) and B for the banded region (d) are plotted for the indicated spherulite. (e) 2D representation of PBS crystal planes (020) and (110), along with SAXS data: when these planes are oriented almost parallel to the X-ray nanobeam, they exhibit maximum intensity in the plot of normalized reflection intensity versus distance from the spherulite center.

Figure 4e schematically depicts the orientation of the 020_PBS_ and 110_PBS_ crystal planes relative to the lamellae and the incident nanobeam. When these planes are almost parallel to the beam, constructive interference enhances the diffraction intensity. The periodic intensity modulation in the banded region thus reflects a regular twisting of the lamellae, consistent with WAXD evidence of continuous crystal rotation. In contrast, the nonbanded area maintains constant intensity, confirming stable lamellar alignment. The alternating maxima and minima in WAXD and SAXS intensities correspond to the periodic crystal reorientation that gives rise to the quadrant-specific spherulitic architecture (schematic in Figure 4e).

Hydrolytic degradation resulted in minimal weight loss over 9 weeks (S2.8 section in the SI). In contrast, enzymatic degradation with lipase was more effective,^4^ especially for PCL (Figure 5a). Enzymes cleave ester bonds in PCL,^40^ causing surface erosion, while hydrolytic degradation proceeds via bulk erosion.^41^ After 35 days, PCL lost ∼61% of its weight under enzymatic conditions, while PBS lost less than 1%, confirming its high resistance to lipase enzymatic degradation.^42–45^ Nonetheless, PBS degradability can increase significantly under specific environmental or enzymatic conditions.^4^

**Figure 5:**
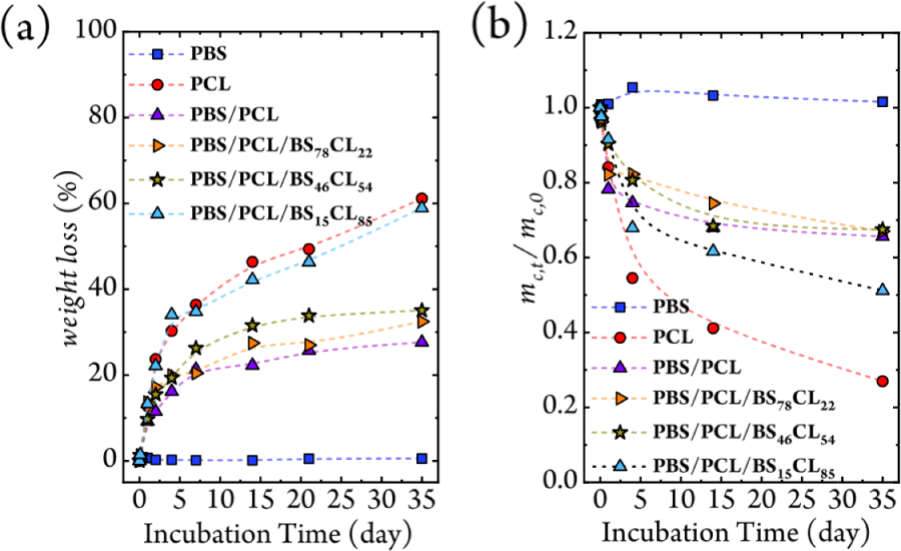
(a) Biodegradation results of homopolymer, binary blend, and ternary blend samples. (b) Relative amount of crystalline mass (*m_c,t_* /m_*c,*0_) as a function of degradation time.

The binary PBS/PCL blend exhibited intermediate enzymatic degradation behavior, governed primarily by the PCL segments. In contrast, the ternary blends showed significantly enhanced biodegradation upon the incorporation of only 10 wt % of the BS_*x*_CL_*y*_ copolyester. This acceleration likely arises from PCL’s lower crystallinity (see Table S3), which facilitates water diffusion and enzyme penetration.

This accelerated degradation properly correlates with the predominance of β-form PBS crystals in the ternary blends, whose looser molecular packing and larger lattice parameters increase interlamellar free volume; parameters that have recently been postulated to affect enzymatic degradation mechanisms.^46^ Remarkably, the CL-rich BS_15_CL_85_ copolymer degrades almost as rapidly as neat PCL, demonstrating that fine-tuning the copolymer composition enables precise control over biodegradation kinetics through morphological and crystallization pathways.

DSC analysis for the PCL phase (see Figure S25a,b) shows that Δ*H*_*m*_ decreased, indicating enzymatic attack not only in the amorphous regions but also in the crystalline regions. Complete loss of crystallinity (Δ*H*_*m*_ ≈ 0) occurred after 35 days in the blends, consistent with prior studies.^42–45^ For the PBS phase, Δ*H*_*m*_ increased, suggesting the preferential degradation of the amorphous regions. Overall, blending with PCL promotes biodegradation in PBS-rich phases by facilitating enzyme access to amorphous domains.^47^

The relative amount of crystalline material with respect to its initial value *m*_*t,0*_/*m*_*c,*0_ evolves over time according to eq 1:^48^

1 $$m_{c,t}/m_{c,0}=\left(\Delta H_{m,t}/\Delta H_{m,0}\right)\left(1-m_{L,t}\right)$$

where Δ*H*_*m,*0_ is the heat of fusion of the material at the beginning, and Δ*H*_*m,t*_ is the heat of fusion at time *t*. *m*_*L,t*_ denotes the mass loss at time *t*. In Figure 5b, for neat PBS, an increase in the ratio *m*_*t,*0_/*m*_*c,*0_ over time indicates ongoing (re)­crystallization during the early stages of the experiment, up to approximately 5 days. Beyond this point, the ratio remains almost constant, with no mass loss observed, confirming that PBS is largely resistant to enzymatic degradation. In contrast, both PCL and the PBS/PCL binary and ternary blends display continuous decreases in *m*_*t,*0_/*m*_*c,*0_. This trend reflects progressive enzymatic degradation of their crystalline domains, driven primarily by the susceptibility of the PCL segments, whether as a homopolymer or a copolymer.

These degradation analyses reflect the overall degradation rate but do not reveal the specific degradation mechanism, and further investigation is required in future work.

## Conclusions

Isodimorphic BS_*x*_CL_*y*_ copolyesters enable the compatibilization of immiscible PBS/PCL blends through a matrix-driven crystallization mechanism. BS-rich compositions promote cocrystallization with PBS, giving rise to structurally coupled quadrant-specific spherulites composed of alternating banded and nonbanded quadrants. Nanobeam synchrotron X-ray mapping directly visualizes continuous lamellar twisting and spatially periodic reorientation of crystal planes, revealing the polymorphic interplay between α- and β-form crystals that supports this quadrant-specific morphology. These molecular interactions translate into tunable crystallinity and enzymatic degradation kinetics. CL-rich compatibilizers, by contrast, reduce cocrystallization and favor more open, enzyme-permeable morphologies, leading to faster enzymatic degradation. Altogether, this study establishes matrix-directed isodimorphic crystallization as a powerful design principle for tuning the morphology and end-of-life biodegradability in sustainable polyester blends.

## Supplementary Material



## Notes

Materials and methods, experimental procedures, synthesis results of polymer samples, polymer characterization data via DSC, SEM, PLOM, WAXD, SAXD, FT-IR, PFT-IR, nanobeam X-ray, and hydrolytic/enzymatic degradation results (PDF)

## Abbreviations

*T*_g_: glass transition temperature
DSC: differential scanning calorimetry
PBS: poly­(butylene succinate)
PCL: poly­(ε-caprolactone)
BS_*x*_CL_*y*_: PBS-*ran*-PCL copolyesters
PLOM: polarized light optical microscopy
FT-IR: Fourier transform infrared
PFT-IR: polarized FT-IR
WAXD: wide-angle X-ray Diffraction
SAXS: small-angle X-ray scattering.
