# Supplementary material for: Crystallization-Driven Quadrant-Specific Spherulitic Self-Assembly in Partially Miscible Biodegradable PBS/PCL/PBS-ran-PCL Blends
Source: J Am Chem Soc. 2026 Mar 30;148(18):18782–90. doi: 10.1021/jacs.5c22442 (PMC13185093; doi:10.1021/jacs.5c22442)
Supplement: Supplementary file 1 [file ja5c22442_si_001.pdf]

Crystallization-driven Quadrant-Specific Spherulitic Self-assembly in Partially Miscible Biodegradable PBS/PCL/PBS-ran-PCL Blends

Maryam Safari [1]*, Roy Kneepkens [1], Ricardo A. Pérez-Camargo [2]*, Agurtzane Mugica [2], Manoli Zubitur [3], Manfred Burghammer [4], Guoming Liu [5,6], Dujin Wang [5,6], Jules A.W. Harings [1], and Alejandro J. Müller [2,7]*

[1] *Maastricht University-Aachen Maastricht Institute for Biobased Materials (AMIBM), Urmonderbaan 22, Geleen 6167 RD, The Netherlands*

[2] *POLYMAT and Department of Polymers and Advanced Materials: Physics, Chemistry and Technology, Faculty of Chemistry, University of the Basque Country UPV/EHU, Paseo Manuel de Lardizabal 3, 20018, Donostia-San Sebastián, Spain*

[3] *Chemical and Environmental Engineering Department, Polytechnic School, University of the Basque Country UPV/EHU, Plaza Europa 1, 20018, Donostia-San Sebastián, Spain*

[4] *European Synchrotron Radiation Facility, 6 rue Jules Horowitz, BP220, 38043 Grenoble, cedex 9, France*

[5] *Beijing National Laboratory for Molecular Sciences, CAS Key Laboratory of Engineering Plastics, Institute of Chemistry, Chinese Academy of Sciences, Beijing 100190, China*

[6] *University of Chinese Academy of Sciences, Beijing 100049, China*
[7] *IKERBASQUE, Basque Foundation for Science, Plaza Euskadi 5, 48009, Bilbao, Spain.*

*Corresponding authors: maryam.safari@maastrichtuniversity.nl, ricardoarpad.perez@ehu.eus, alejandrojesus.muller@ehu.es

S1. Experimental

S1.1. Materials

Commercial PBS and PCL were used to prepare binary (50/50 PBS/PCL) and ternary blends as indicated below. Nature-Plast supplied the PBS under the trade name PBE003 with a number average molecular weight, M_n = 19,800 g/mol[1]. Ravago Chemicals Spain provided the PCL under the trade name Capa-6250, with a weight average molecular weight, M_w = 25,000 g/mol. The main characteristics of these samples are listed in Table 1.

The third component in preparing ternary blends was BS_xCL_y copolymers in different compositions determined by Nuclear magnetic resonance, H^1 NMR (see Table 1). These copolymers were synthesized and characterized in previous works.[2, 3] The synthesis involved transesterification and the ring-opening reaction of dimethyl succinate (DMS), 1,4-butanediol (BD), and ε-caprolactone (CL), resulting in high-molecular-weight BS_xCL_y copolymers. The weight average molecular weight *(M_w)* and number average molecular weight *(M_n)* were determined using Size Exclusion Chromatography (SEC) with poly(methyl methacrylate) (PMMA) standards and HFIP solvent (see Table 1).

Table S1. Results (M_n, M_w, and dispersity, *Đ*) of the synthesis of the copolymerization of BS and CL.

Copolyester	M_n (g/mol)	M_w (g/mol)	*Đ*
PBS	19,800	79,250	4.0
$BS_{78}CL_{22}$	19,750	51,400	2.6
$BS_{46}CL_{54}$	26,000	60,700	2.3
$BS_{15}CL_{85}$	26,350	53,100	2.0
PCL	N.A.	25,000	N.A.

Preparation of the Binary and Ternary blends

The neat materials, binary (i.e., PBS/PCL blends), and ternary blends (see Table 2) were prepared by melt extrusion mixing using the mini extrusion (DSM-Micro-5) technique due to the limited amount of copolymers available. The ternary blends were formulated with a 50/50/10 PBS/PCL/BS_xCL_y composition. In all cases, BSxCLy copolymers (see Table 1) were incorporated as compatibilizers, accounting for 10% of the total weight of the PBS/PCL blend. Before extrusion, the PBS and PCL were dried in a vacuum oven at 80 ºC and 40 ºC for 24 hours, respectively. During extrusion, the speed was maintained at 150 rpm, while the

temperature was set at 150 ºC. The 50/50 PBS/PCL composition was chosen as a model system because it signifies the most challenging condition for compatibilization, where both phases are present in comparable volume portions and the largest interfacial area is shaped. Under these conditions, interfacial phenomena such as matrix-directed crystallization can be most clearly seen, allowing the effectiveness of the random copolymer compatibilizer to be evaluated unambiguously. In addition, the crystallization kinetics of PBS and PCL are adequately comparable near this composition, enabling competitive crystallization to occur and making it possible to directly observe how the copolymer controls resulting spherulitic morphology.

Three representative random copolymer compositions (BS-rich, pseudo-eutectic, and CL-rich) were selected to systematically probe the influence of copolymer chemical structure on crystallization in PBS/PCL blends. The pseudo-eutectic composition was chosen because it represents the critical condition at which the crystallization tendencies of the BS and CL segments are balanced, allowing competitive crystallization to occur without dominance of either component. This composition therefore provides a sensitive reference point for evaluating interfacial crystallization behavior. In contrast, the BS-rich and CL-rich copolymers define the upper and lower compositional limits of segmental participation in co-crystallization with the surrounding matrix, enabling assessment of how deviations from the balanced composition alter the resulting spherulitic morphology. Together, these three compositions span the key compositional regimes necessary to establish the structure-crystallization relationships governing the compatibilization mechanism.

Table S2. Samples used in this study.

Samples without compatibilizer	Samples with compatibilizer
1. PBS	4. PBS/PCL/$BS_{78}CL_{22}$ (50/50/10)
2. PCL	5. PBS/PCL/$BS_{46}CL_{54}$ (50/50/10)
3. PBS/PCL (50/50)	6. PBS/PCL/$BS_{15}CL_{85}$ (50/50/10)

S1.2. Characterization techniques

The neat materials and the binary 50/50 PBS/PCL blends served as references, and the ternary blends were characterized using various techniques.

Differential Scanning Calorimetry (DSC)

A PerkinElmer (8000-Pyris model) was used to perform DSC measurements. It features a cooling system (Intracooler 2P) and operates under a nitrogen flow of 20 mL/min. Calibration was performed with indium (T_m = 156.61 °C and ΔH_m = 28.71 J/g). The samples (~ 5 mg) were sealed in standard aluminum pans.

The samples were measured under non-isothermal conditions, with the first heating, cooling, and second heating scans recorded. Initially, the as-received samples were heated (first heating scan) from 25 °C to 150 °C, holding at 150 °C for 3 minutes to eliminate prior thermal history. Subsequently, the sample was cooled from the melt (cooling scan) to -60 °C, holding this final temperature for 1 minute, establishing a standard thermal history. Finally, the sample was heated from -60 °C to 150 °C (second heating scan). All scans were conducted at a scanning rate of 20 °C/min.

Scanning Electron Microscopy (SEM)

A SEM from the Hitachi brand (TM3030-Plus model) with an acceleration voltage of 15 kV was used to further examine the morphology of the reference binary blend, namely, the PBS/PCL blend, as well as the ternary blends containing different compatibilizers. Extruded samples were utilized and subsequently subjected to a hot-press process. Next, we employed the cryo-fracturing technique to prepare the samples for SEM. Each sample was immersed in liquid nitrogen for 2 hours before being shattered into small pieces. The surface of the samples was coated with a thin layer of gold. The samples were securely held on a sample holder using double-sided tape. The average size of the droplets was calculated using *ImageJ* software.

Polarized Light Optical Microscopy (PLOM)

The morphology of the samples was studied using an Olympus BX-51 optical microscope equipped with a digital camera (Olympus SC-50). Two methods were employed to prepare the samples for PLOM analysis to investigate the effects of top confinement and sample thickness on spherulite morphology.

1. Solvent and top-free surface method: In this approach, 50 mg of each sample was dissolved in 10 mL of $CHCl_3$. Next, 1–2 drops of the solution were placed on a glass slide (without a top cover) and allowed to dry at room temperature for 1-2 hours. The final thickness of the samples was approximately 30 μm.

2. Sandwiched method: A small amount of each sample was placed on a hot stage (Linkam, TP-91 model) and melted. The molten sample was then pressed between two glass slides. The final thickness of the samples was approximately 100 μm.

After sample preparation, a two-step crystallization thermal protocol was applied: (1) Erase the thermal history at 150 °C; (2) Cool at 50 °C/min (the maximum controlled cooling rate allowed by this Linkam equipment) to 85 °C and maintain at 85 °C for 10 minutes to observe PBS crystallization with PCL in the molten state; (3) Cool at 50 °C/min to 40 °C for specified durations to observe the subsequent PCL crystallization. The morphological observations from this protocol allow for the identification of the miscibility features of the 50/50 PBS/PCL blends and how the copolymer addition affects the morphology. Under the two-step crystallization protocol, a unique banded/non-banded pattern morphology was detected.

Non-isothermal PLOM experiments were conducted to investigate whether the banded/non-banded pattern is solely produced during a two-step crystallization process: erasing the thermal history at 150 ºC, then cooling at 10 ºC/min to 20 ºC.

A peculiar mixture of banded and non-banded defined regions was observed in the spherulites of the ternary blends. Further investigations into this pattern were conducted on the spherulites using the previously mentioned thermal protocol with different T_c values. Two experimental setups were performed: (a) the sample was rotated using a Leica DMLP while the polarizers remained fixed, and (b) the polarizers were rotated while the sample stayed in place. These experiments aimed to verify whether the observed pattern represents a true extinction pattern.

Fourier transform infrared spectroscopy (FT-IR)-microscopy

The PLOM experiments revealed a complex morphology in the ternary blends. To investigate molecular-level changes in PBS spherulites within these samples, FT-IR was performed at 85 °C, a temperature at which PBS spherulites form and PCL remains molten. FT-IR spectra of the PBS/PCL/$BS_{78}CL_{22}$ blend were recorded specifically in the banded and non-banded regions of a single spherulite using a PerkinElmer Spotlight 400 FT-IR microscope, covering a spectral range of 4000 to 600 cm^{-1} with a spectral resolution of 2 cm^{-1} and 64 accumulations. Additionally, Polarized FT-IR (PFTIR) measurements were conducted under the same conditions using a gold wire grid polarizer (Perkin-Elmer) at various angles (0°, 45°, 90°, 135°, and 180°) relative to the glass substrate.

Simultaneous Synchrotron Radiation Wide-Angle X-ray Diffraction, and Small-Angle X-ray Scattering (WAXD/SAXS)

Simultaneous *in situ* WAXD/SAXS experiments were conducted on selected samples under non-isothermal conditions at beamline BL11-NCD of the ALBA Synchrotron radiation facility in Barcelona, Spain. This beamline offers two detectors that operate concurrently: the Rayonix LX255-HS, which has an active area of 230.4 × 76.8 mm (pixel size: 44 μm^2) as the

WAXD detector, and the Pilatus 1M from Dectris, featuring an active image area of 168.7 × 179.4 mm^2, a total pixel count of 981 × 1043, and a pixel size of 172 × 172 μm^2 as the SAXS detector. The detectors were positioned differently. The WAXD configuration included a sample–detector distance of 15.5 mm with a tilt angle of 27.3°, while the SAXS employed a distance of 6.463 m. The energy of the X-ray source was set to 12.4 keV (λ = 1.0 Å). The intensity profile was presented as a plot of scattering intensity versus scattering vector, $q = 4\pi \sin(\theta)\lambda^{-1}$, where λ is the X-ray wavelength (λ = 1 Å) and 2θ represents the scattering angle. The scattering vector was calibrated using silver behenate for SAXS and chromium(III) oxide for WAXD.

The samples in the DSC pans were placed in a Linkam hot stage (THMS-600 model) that is equipped with a liquid nitrogen cooling system. The non-isothermal DSC experiment was replicated in the Linkam hot stage, capturing SAXS/WAXD spectra every 6 seconds.

Nanobeam X-ray imaging

Nano-focused synchrotron X-ray scattering experiments were performed to resolve the local crystalline structure along the spherulitic radii. Ternary PBS/PCL/BSxCLy blends were analyzed at the ESRF ID13 beamline using a high-energy X-ray beam (15 keV) focused on a sub-70 nm spot with a sample area of 60 × 120 μm^2. The acquisition time was 0.01 s. Azimuthal integration of all diffraction patterns was automatically performed at the ESRF immediately after acquisition.

Image reconstruction was performed by taking the intensity (counts) at a given *q*-value (±0.2 nm-1) from the integrated data for each measured pixel and colormapping the intensities, resulting in a 2D image of intensity at a *q*-value versus location in the sample.

Hydrolytic Degradation Test

Hydrolytic degradation was performed on the blends. Film samples were prepared using the hot-press method (Collin P200E) at 150 ºC and 150 bar. The material was placed into the chosen mold and sandwiched between two metal plates. Teflon papers were used to prevent the material from adhering to the metal. The resulting blend was cut into small pieces, which were then placed into the test tubes. Next, a buffer solution at pH = 12 (Pan Reac Appli Chem ITW Reagents) was added and kept in an incubator at 37 ± 0.5 ºC while being shaken at a speed of 20 rpm. Each week, three samples were withdrawn from each blend composition and thoroughly washed with distilled water before being placed in a vacuum oven for 24 hours at 30 ºC to remove all moisture. Samples were weighed before and after the degradation process, and the average percentage of mass loss for each blend was calculated.

Biodegradation Test: Enzymatic degradation

The enzymatic degradation was carried out using neat materials and ternary blends, employing Lipase from *Pseudomonas Cepacia* obtained from Sigma Aldrich and a buffer

solution (pH = 7 ± 0.01 at 25 ºC) sourced from Labprocess. Approximately 100 mg of the blended films were submerged in a sodium phosphate buffer solution containing 5 mg/mL of lipase (*Pseudomonas Cepacia*). The vials were continuously shaken in an incubator at 37 ± 0.5°C with a speed of 20 rpm. Three samples of each type of blend were extracted at predetermined intervals from the buffer-lipase solution, rinsed with distilled water, and thoroughly dried in a vacuum oven at 30 ºC for 24 hours. Samples were weighed before and after the degradation process, and the average percentage of mass loss for each blend was calculated.

S2. Results that are not included in the main text

S2.1. Thermal transitions (DSC non-isothermal experiments)

Figure S1 indicates that neat PBS crystallizes and melts at higher temperatures than neat PCL, consistent with reports in the literature [4, 5] Blending PBS and PCL in a 50/50 composition results in macro-phase separation of the amorphous[6, 7] molten, and solid states due to their immiscibility. This phase separation is evidenced in the amorphous state by two T_g values, T_g = -31.6 °C (PBS) and -63.5 °C (PCL) (see Figure S3 and Table S1). In the solid state, immiscibility is evidenced by two separate T_c and T_m values corresponding to the PBS and PCL phases of the blend. These thermal transitions show only minor shifts in their positions, indicating that the blend is immiscible, as these transitions closely resemble those of the neat materials[8] It is important to note that neat PBS and PBS in the blend exhibit multiple endothermic peaks. These peaks have been the subject of recent investigation and could be linked to the melting of two polymorphs of neat PBS or reorganization during the scans.[9]

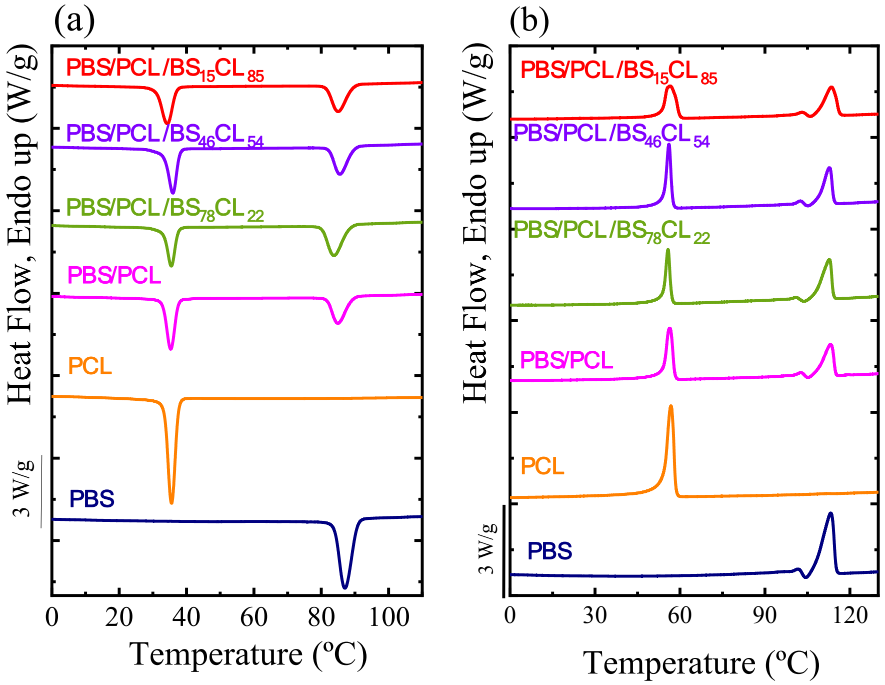

Figure S1. DSC (a) Cooling, and (b) Second heating experimental scans, performed with a scanning rate of 20 ºC/min for neat PCL and PBS, and their binary and ternary blends.

The minor changes in the thermal transitions in the 50/50 PBS/PCL blend have been studied in detail. In the blend, the PBS phase transition temperatures and enthalpies are shifted to lower values than in the neat PBS. In contrast, the PCL phase-related values are only slightly shifted when compared to neat PCL. These results are similar to those reported by John et al.[10] in the 50/50 PBS/PCL blend of commercial PBS and PCL. Other compositions, such as 80/20 PBS/PCL blends, have been studied by Liu and Zhou[11] and Wang et al. [12] Overall, it has been found that molten PCL has a minimal impact on the thermal transitions of PBS, while the PBS phase, which crystallizes first, can nucleate and confine the PCL phase, thereby influencing its thermal transitions. The nucleating effect may be hindered in 50/50 PBS/PCL blends due to the increased immiscibility associated with this composition.

Adding 10% of the BS_xCL_y copolymer to the 50/50 PBS/PCL blend results in some changes in the thermal transitions, indicating an interaction between the compatibilizer and the blend's components. In the amorphous state, adding the copolymer decreases the T_g values of the PBS phase while increasing those of the PCL phase (see Figure S1 and Table S1). The shift in the T_g values of each phase toward the other serves as evidence of partial miscibility, and interactions between the two polymers facilitated by the compatibilizer. In this instance, the interactions between the copolymer and homopolymers are influenced by the copolymer

composition and its isodimorphic nature, specifically the balance of comonomer exclusion and inclusion. A higher level of comonomer exclusion indicates a limited interaction between the BS and CL fractions of the copolymer, restricting its co-crystallization ability and not enhancing the miscibility of the PBS and PCL phases in the blend. Conversely, a compatibilization effect is anticipated for the copolymer with a lower comonomer exclusion.

Figures S1a and b reveal no transitions of the BS_xCL_y copolymer during the cooling or heating scans of the blends. However, these transitions are expected (refer to the displayed BS_xCL_y transitions) according to the sum curves in Figures 1a and b. The thermal transitions of all the copolymers occur at different temperatures than those of the PBS and PCL phases, as seen in Figures 1a and b. For the $BS_{46}CL_{54}$, the BS fraction of the copolymer melts at the same position as the PCL, which may create an overlapping effect. Therefore, the absence of BS_xCL_y transitions in the blends, as shown in Figures Sa and b, indicates that the copolymers either remain in the amorphous region or co-crystallizes with the PBS or PCL phase, depending on the composition. The lack of an independent transition signal for the BSxCLy copolymer demonstrates its miscibility with the PBS and PCL components of the 50/50 PBS/PCL blend.

Another evidence of a miscible or partially miscible character of the ternary blend is the shifting of its thermal transitions compared with the binary blend. Figure 2 shows the T_c and T_m values and the corresponding enthalpies (crystallization, ΔH_c, and melting, ΔH_m, enthalpies) as a function of the BS content for the PBS and PCL phases. For a BS content of zero, the plotted values correspond to the PBS or PCL phase of the 50/50 PBS/PCL blend, and they are indicated with a horizontal dashed line.

Figure S2a illustrates that both T_c and T_m values exhibit slight variations with the BS content of the copolymer without following a specific trend. For the enthalpies, as shown in Figure S2c, the values increase with increasing crystallinity (Xc) as the BS content rises. This may suggest a degree of co-crystallization between the PBS phase and the BS fraction (the BS component of the BS_xCL_y copolymer), which is encouraged by a reduced exclusion of CL co-units in the BS-rich copolymer, such as $BS_{78}CL_{22}$. These co-crystallization phenomena enhance the copolymer's compatibilization effect. Additionally, the increase in PBS phase T_g values indicates positive interactions between the PBS and BS fractions, demonstrating improved miscibility.

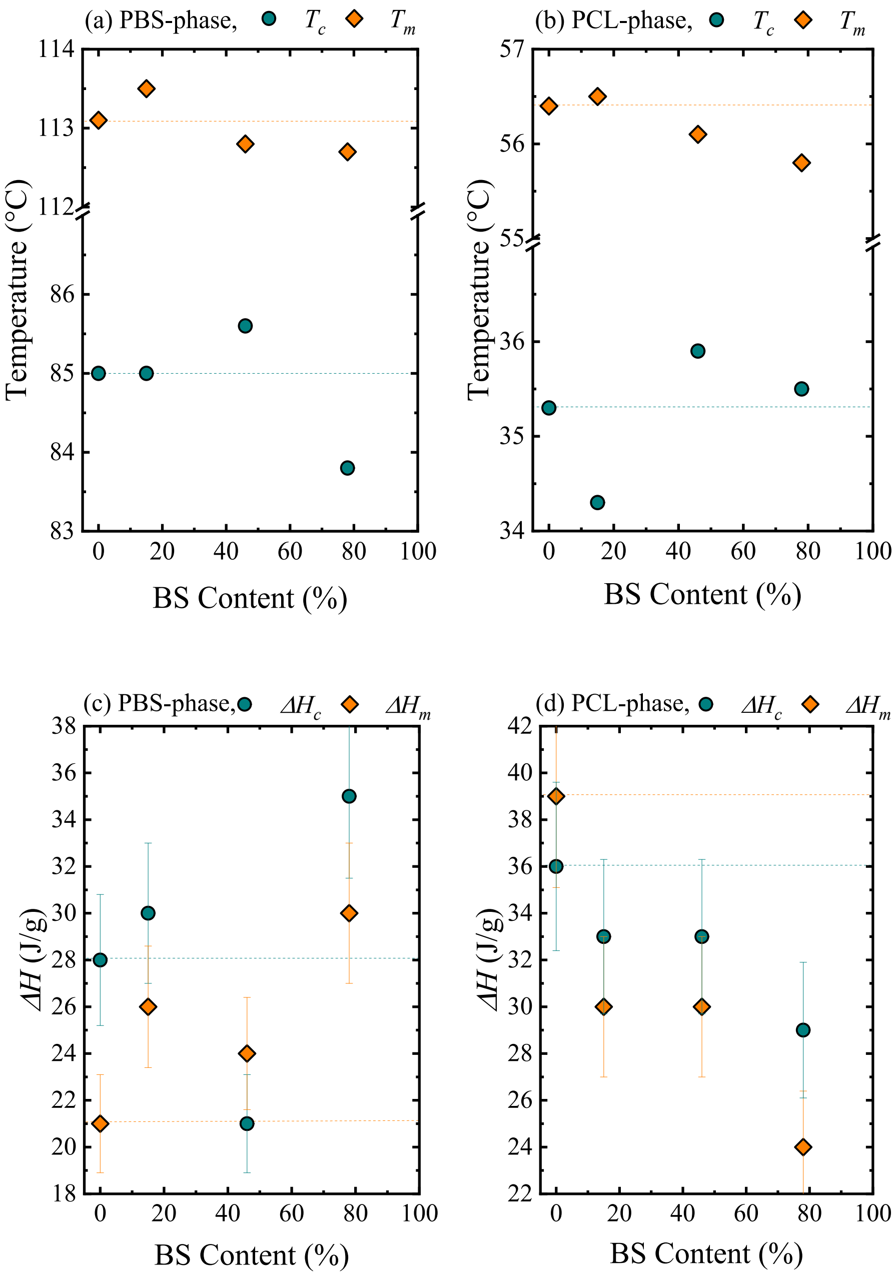

Figure S2. Crystallization and melting temperatures (a, b) and the corresponding enthalpies (c, d) as a function of the BS content of BS_xCL_y copolymer in the 50/50/10 PBS/PCL/BS_xCL_y blend for PBS (a, c) and PCL phase (b, d). The values at BS content of 0% correspond to the PBS or PCL phase in the 50/50 PBS/PCL blend and are also indicated with a horizontal dashed line.

The T_c and T_m values change slightly with the BS content for the PCL phase, as shown in Figure S2b. At high BS content, specifically, $BS_{78}CL_{22}$ and $BS_{46}CL_{54}$, the T_c increases while the T_m decreases compared to the values of the PCL phase in the 50/50 PBS/PCL blend. The rise in the T_c values of the PCL phase at elevated BS content aligns with the findings of Liu and Zhou[53], who reported an increase in the T_c values of the PCL phase when 5% of BS_xCL_y copolymer, with a molar ratio of 0.473, was added to an 80/20 PBS/PCL blend. This phenomenon can be attributed to a nucleation effect of the PBS phase, which crystallizes first and shows enhanced crystallization in the PCL phase due to the presence of the BS fraction.

Figure S2d indicates that the enthalpy values of the PCL phase decrease as the BS content increases. Consequently, the PBS phase nucleated the PCL phase and confined it, which affected crystallization. For low BS content, such as $BS_{15}CL_{85}$, the excluded BS co-units restricted co-crystallization of the PCL phase and the CL fraction, resulting in a weaker compatibilization effect than in the $BS_{78}CL_{22}$ copolymer.

In conclusion, all the BS_xCL_y copolymers modify the thermal transitions of the 50/50 PBS/PCL blend, indicating a degree of interaction that may enhance the blend's properties. Further evaluation of these ternary blends involved morphological, conformational, structural, and degradation studies.

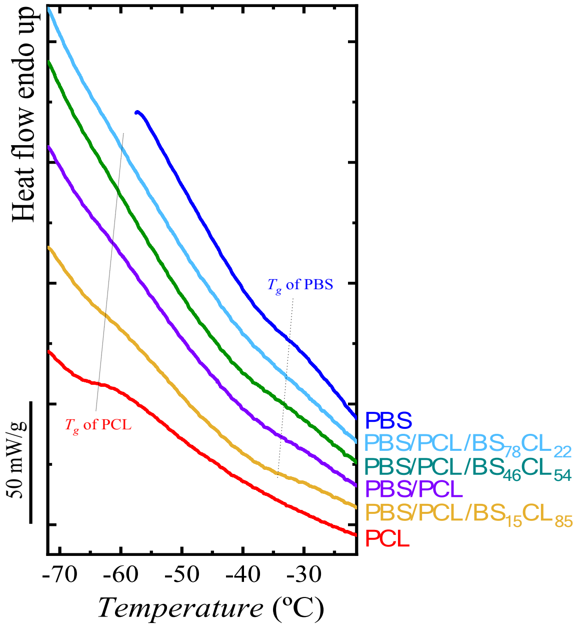

Figure S3: DSC heating runs from -80 ºC at 10 ºC/min for the indicated samples.

Table S3. Thermal transitions of the samples in this study.

	T_c PBS (ºC)	T_c PCL (ºC)	ΔH_c PBS (J/g)	ΔH_c PCL (J/g)	T_g PBS (ºC)	T_g PCL (ºC)	T_{cc} PBS (ºC)	T_m PBS (ºC)	T_m PCL (ºC)	ΔH_m PBS (J/g)	X_c PBS (%)	ΔH_m PCL (J/g)	X_c PCL (%)
PBS	87.04	-----	65	-----	-31.4	-----	107.03	113.95	-----	54	58	-----	-----
PCL	-----	35.5	-----	69	-----	-63.8	-----	-----	56.8	-----	-----	61	44
PBS/PCL	85.0	35.2	28	36	-31.6	-63.5	105.2	113.1	56.4	21	11	39	56
PBS/PCL/$BS_{78}CL_{22}$	83.8	35.6	35	29	-34.7	-61.4	103.9	112.7	55.8	30	17	24	33
PBS/PCL/$BS_{46}CL_{54}$	85.6	35.9	21	33	-34.1	-62.3	104.9	112.7	56.1	24	13	30	39
PBS/PCL/$BS_{15}CL_{85}$	85.00	34.3	30	33	-32.8	-60.7	105.9	113.5	56.6	26	13	30	37

S2.2. Scanning Electron Microscopy (SEM)

SEM enables a deeper observation of the morphology of the blends. First, we analyzed images of the 50/50 PBS/PCL blends (see Figure S4a) compared with those of the compatibilized PBS/PCL/BSxCLy samples (Figure S4b-d).

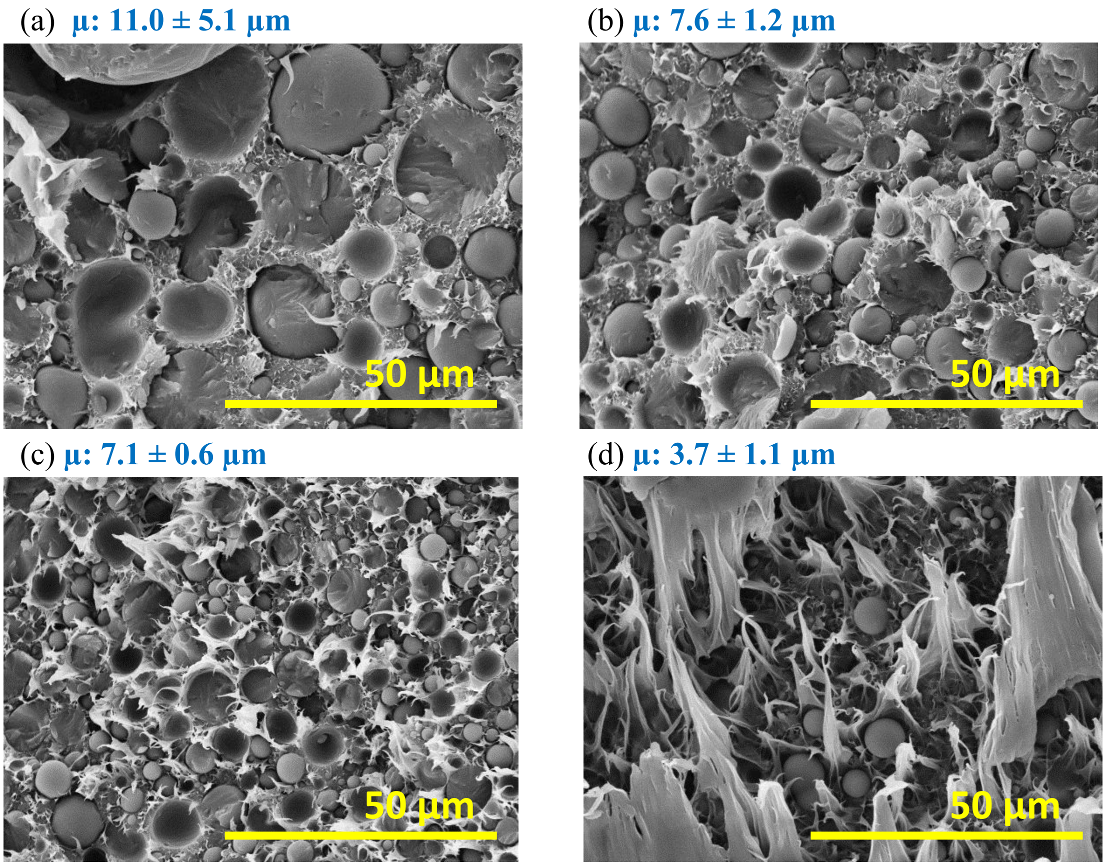

Figure S4. Micrographs obtained by scanning electron microscopy (SEM): (a) 50/50 PBS/PCL, (b) 50/50/10 PBS/PCL/$BS_{78}CL_{22}$, (c) 50/50/10 PBS/PCL/$BS_{46}CL_{54}$ and (d) 50/50/10 PBS/PCL/$BS_{15}CL_{85}$. The average size of the detected droplets is indicated at the top of each Figure.

Figure S4a shows the SEM image for the 50/50 PBS/PCL blend. It reveals a sea-island morphology, in which the blend is segregated into two distinct phases: droplets and a matrix. This morphology is typical of immiscible blends and is consistent with the findings of Wang et al.[12] in their study of 80/20 PBS/PCL blends. It is difficult to determine which phase in the 50/50 PBS/PCL blend corresponds to PBS and which to PCL, as their compositions are similar. One possibility is that there are regions with PBS matrix and PCL droplets, and vice versa. Liu et al. have reported a similar morphology.[11] Another possibility is that PCL is the matrix and PBS is the droplets, as PCL is softer and more stretchable than PBS. Assuming the latter, the average size of the PBS droplets is approximately 11.0 μm.

Figures S4b-c-d show the images of the final morphology when the BS_xCL_y copolymers are employed as a compatibilizer of the 50/50 PBS/PCL blends. There is a phase as a matrix and one as droplets for all the blends. The compatibilizer is expected to reduce the droplets' size, as obtained by Liu and Zhou.[11] for the 80/20/5 PBS/PCL/BS_xCL_y blends. Assuming PBS droplets, Figures S4b, S4c, and S4d show that the size of the PBS droplets decreases with the

addition of the copolymer, evidencing the effect of the compatibilizer. Interestingly, in the $BS_{15}CL_{85}$ blend, the droplets are not uniformly distributed as in $BS_{78}CL_{22}$, indicating a difference in the compatibilization effect between the two blends.

S2.3. PLOM experiments

We have performed PLOM tests on samples obtained from both the solution and the melt, which resulted in different thicknesses. However, we did not observe any differences in the morphology of the samples obtained from the melt or the solution. Therefore, we can conclude that the observed morphology is not affected by the thickness of the samples.
The PLOM images for the neat PCL and PBS are displayed in Figure S5 at *Tc* = 40 °C (for PCL) and 85 °C (for PBS).

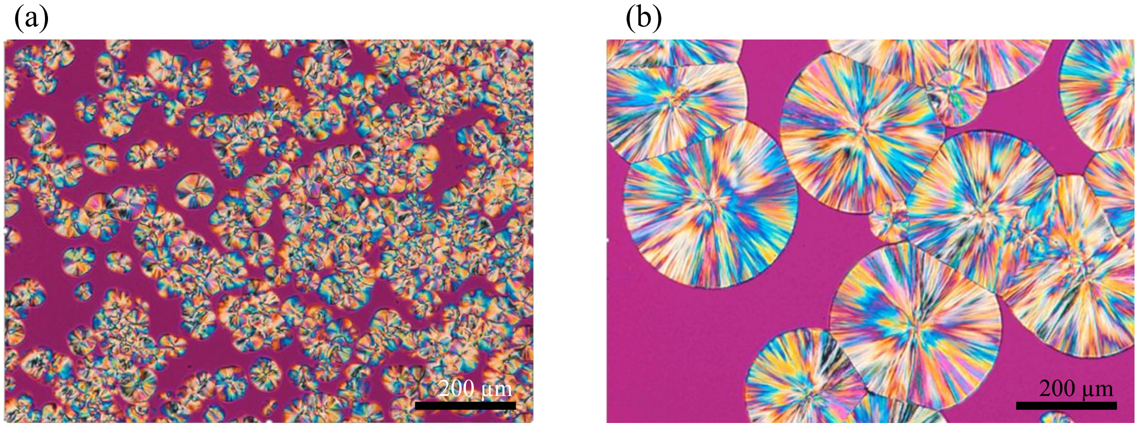

Figure S5. PLOM micrographs for the neat PCL sample at 40 ºC after 2 min (a), and for the neat PBS sample at 85 ºC after 2 min (b).

S2.4. Analysis of the banding/no-banding patterns

The ternary 50/50/10 PBS/PCL/BS_xCL_y blends exhibited a novel banded/non-banded pattern, which is studied in this section. First, the temperature dependence of this novel pattern was investigated, and next, it was proved that the pattern corresponds to a real extinction pattern. For all experiments, the 50/50/10 PBS/PCL/$BS_{78}CL_{22}$ blend was used as the reference.

S2.4.1. Temperature dependence of the banded/non-banded pattern

The 50/50/10 PBS/PCL/$BS_{78}CL_{22}$ blend was crystallized at different T_c values to study the temperature dependence of the banded/non-banded pattern. A set of images is shown in Figure S6.
Figure S6 shows that the banding region changes with T_c. At low T_c, e.g., 70 °C, the banded region is clear and defined, displaying short distances between the bands. As T_c increases, the banded region becomes less clear and defined, with increased band spacing, ultimately leading

to a diffuse banded pattern. In fact, at T_c = 100 °C, the bands are not clear, but a difference between "banded" and "non-banded" regions is observed. It is worth noting that as Figure S5b shows, the PBS at already 85 °C does not show any banding, and instead it shows clear not banded spherulites.

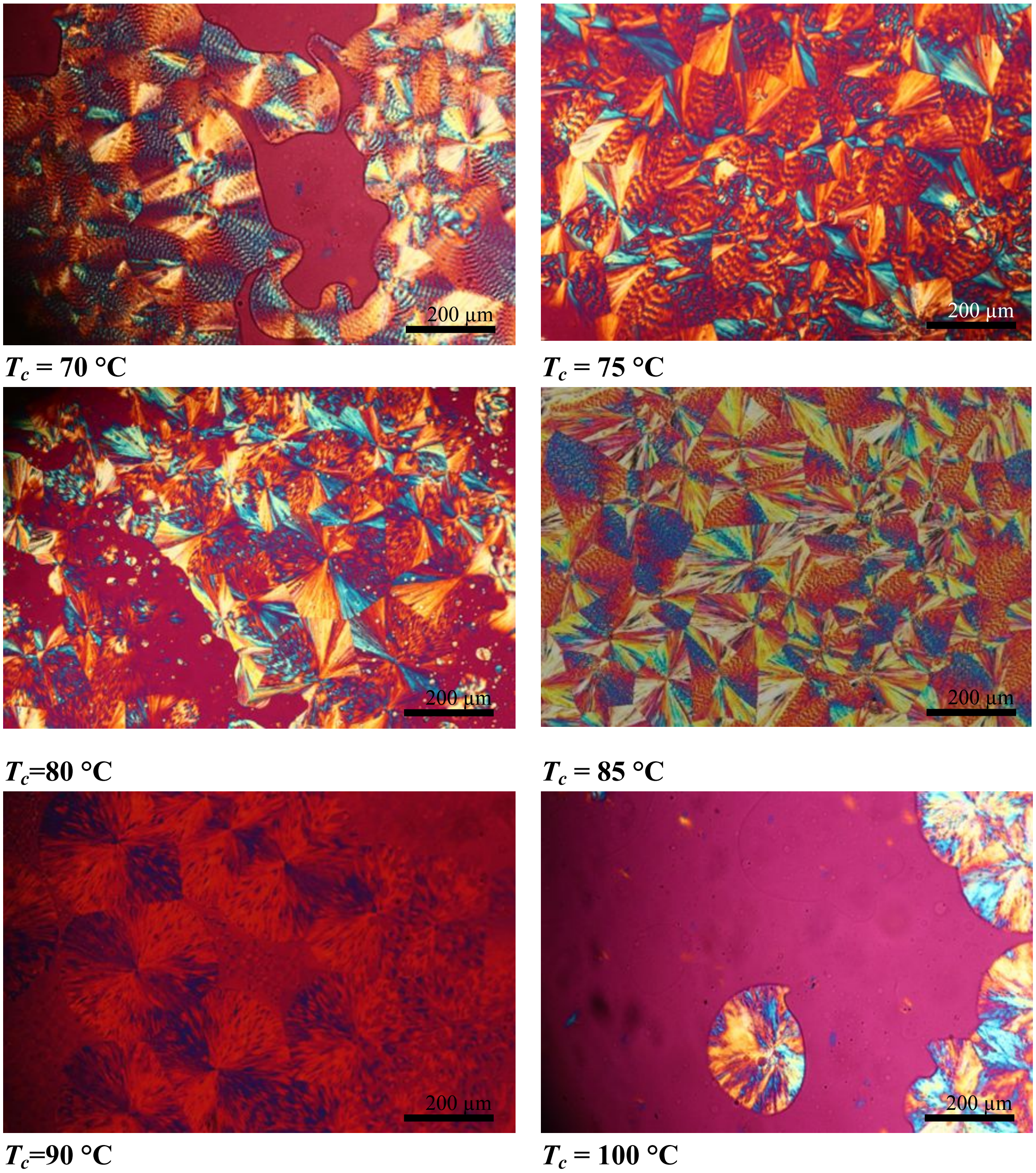

Figure S6. PLOM micrographs for PBS/PCL/$BS_{78}CL_{22}$ 50/50/10 sample at the indicated crystallization temperatures.

S2.4.2. Rotating PLOM experiments

The mixture of banded/no-banded regions observed in the ternary blends' spherulites corresponds to a novel extinction pattern. Two experimental approaches were employed to prove that this extinction pattern is real and thus is present in the 3D spherulitic structure. On the one hand, (a) the polarizers were rotated (maintaining them crossed) with a fixed position of the sample, whereas, on the other hand, (b) the sample was rotated, keeping the polarizers fixed. These experiments were performed in the selected PBS/PCL/$BS_{78}CL_{22}$ 50/50/10 sample, employing different T_c. Figure S7 shows the results on (a) Rotating polarizers with a fixed sample position. The various images were taken with (not shown) and without the lambda plate.

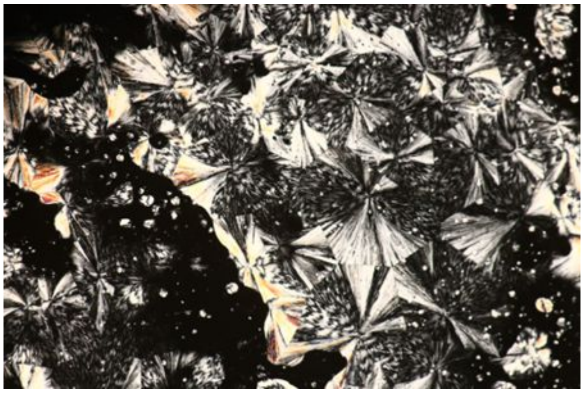

0°, T_c = 80 °C

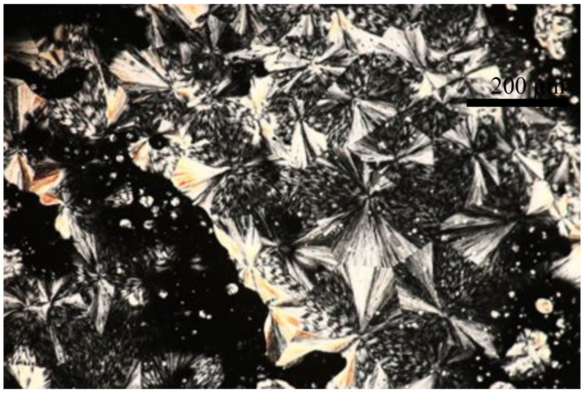

20°, T_c = 80 °C

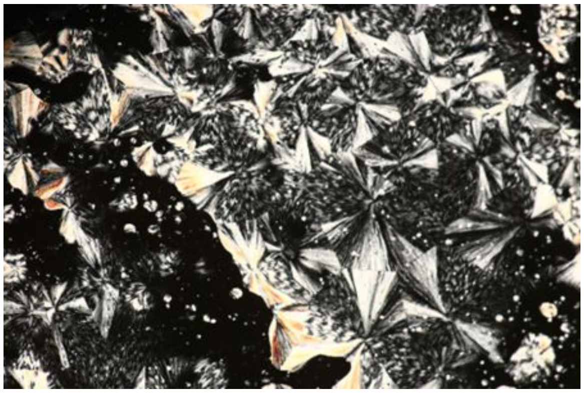

30°, T_c = 80 °C

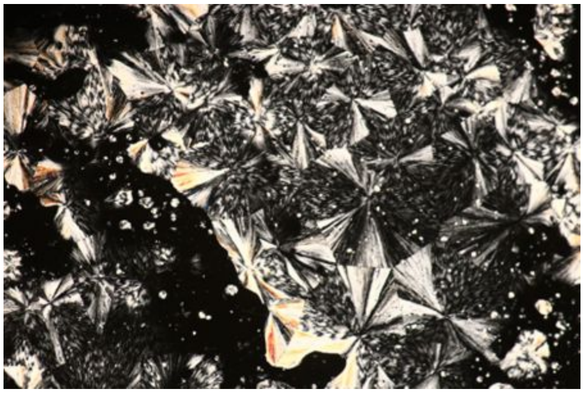

40°, T_c = 80 °C

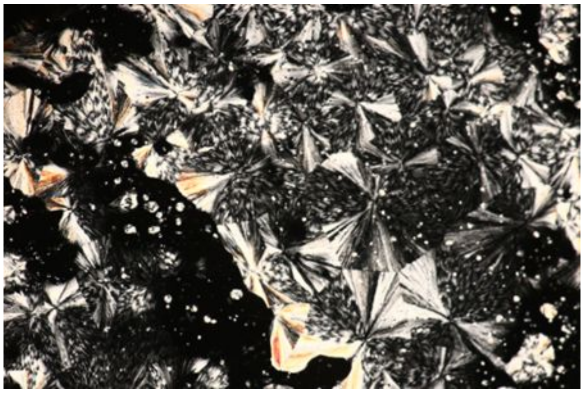

50°, T_c = 80 °C

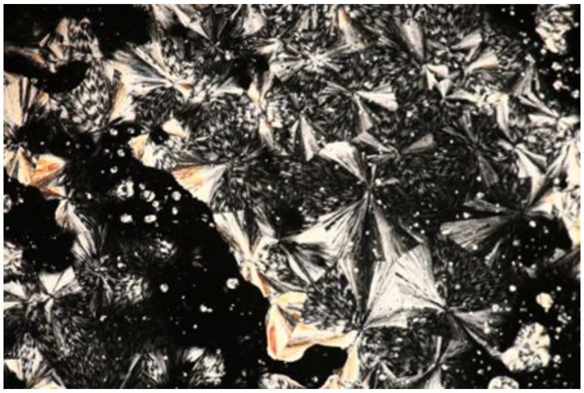

60°, T_c = 80 °C

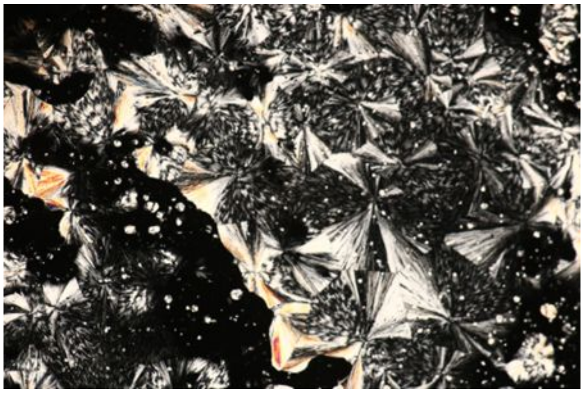
70°, T_c = 80 °C

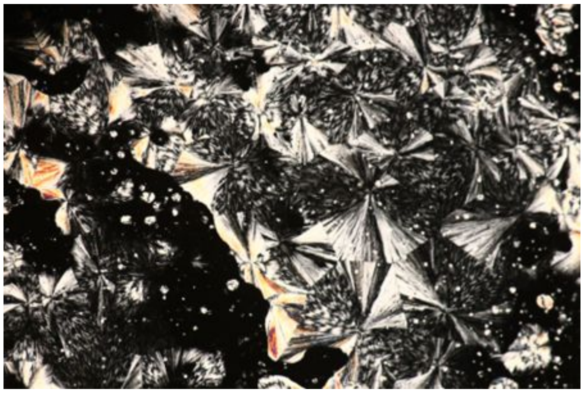
80°, T_c = 80 °C

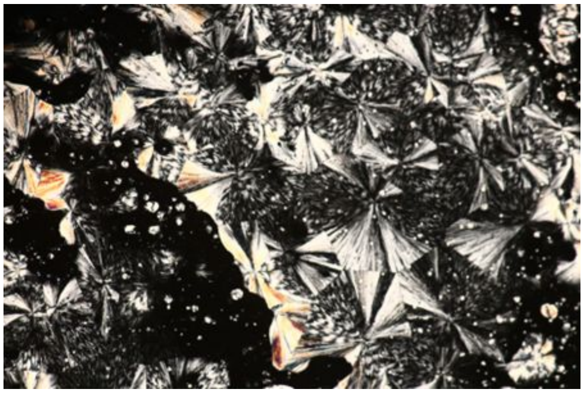
90°, T_c = 80 °C

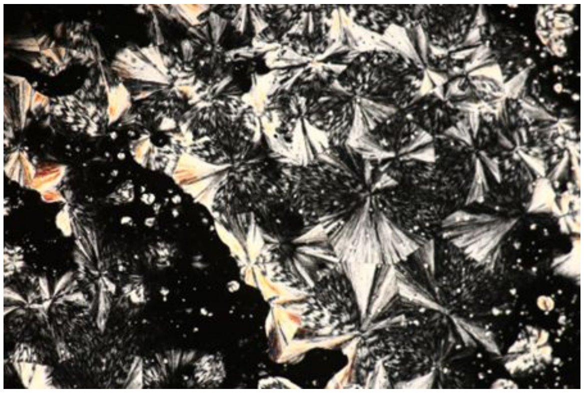
100°, T_c = 80 °C

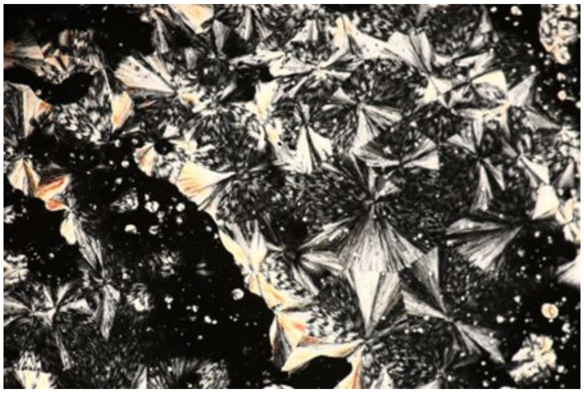
110°, T_c = 80 °C

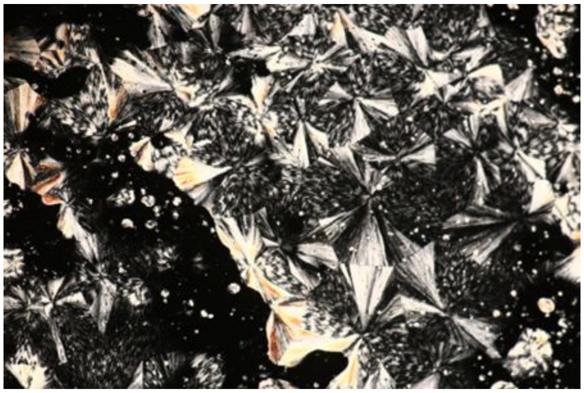
120°, T_c = 80 °C

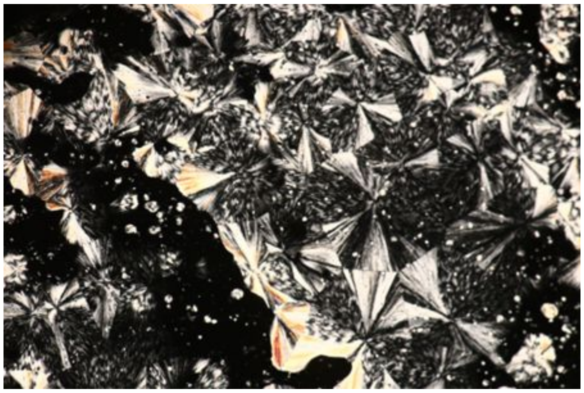
130°, T_c = 80 °C

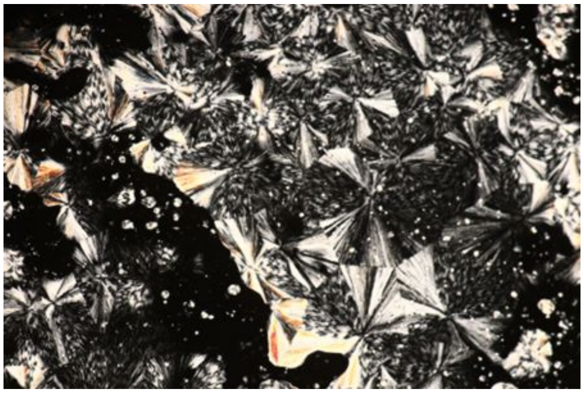
140°, T_c = 80 °C

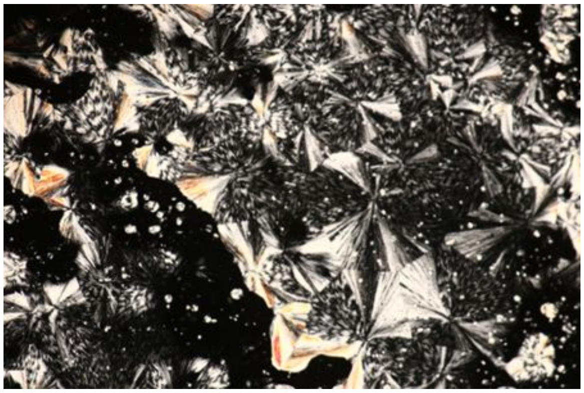
150°, T_c = 80 °C

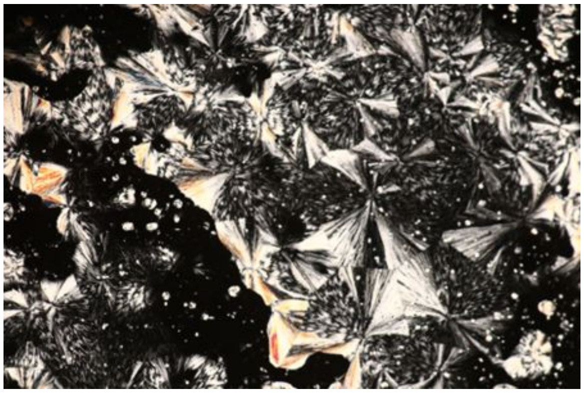
160°, T_c = 80 °C

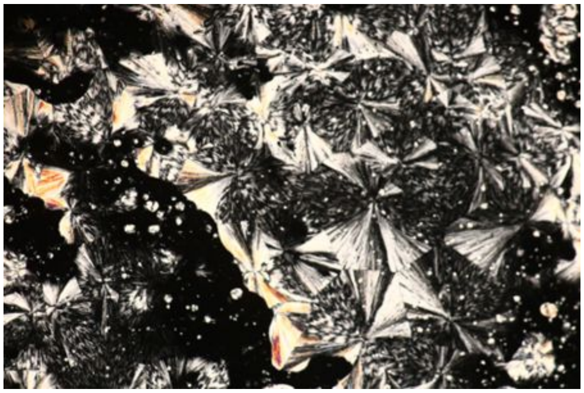
170°, T_c = 80 °C

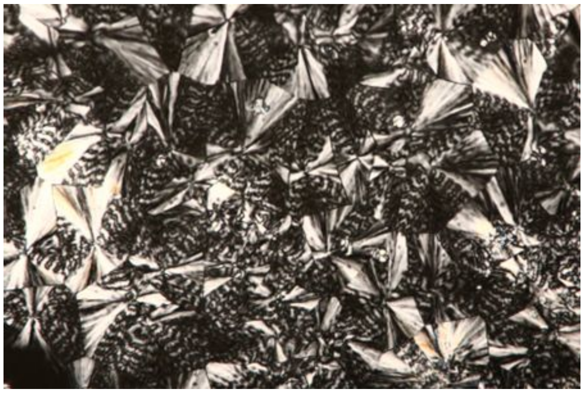
0°, T_c = 75 °C

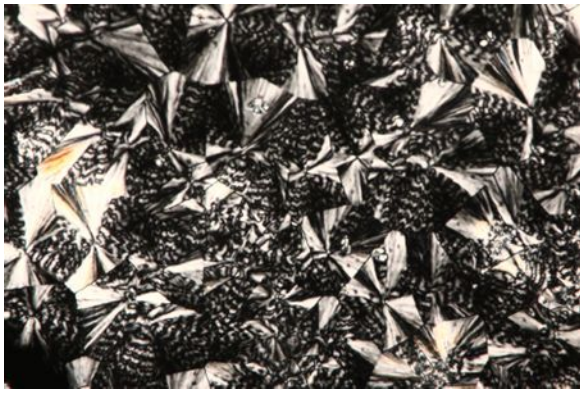
20°, T_c = 75 °C

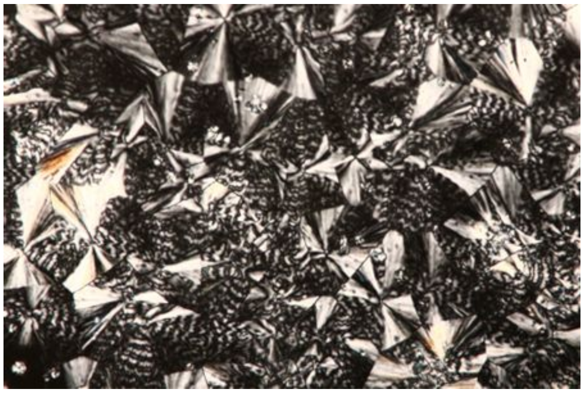
30°, T_c = 75 °C

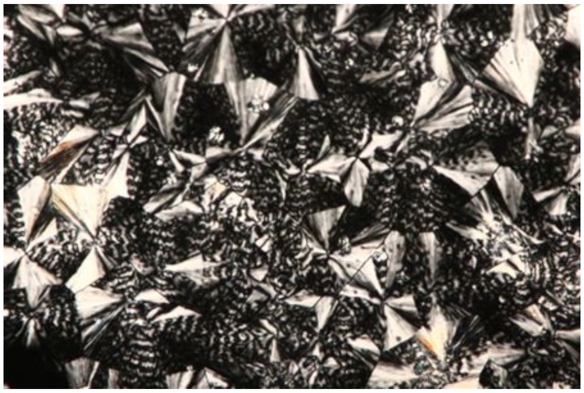
40°, T_c = 75 °C

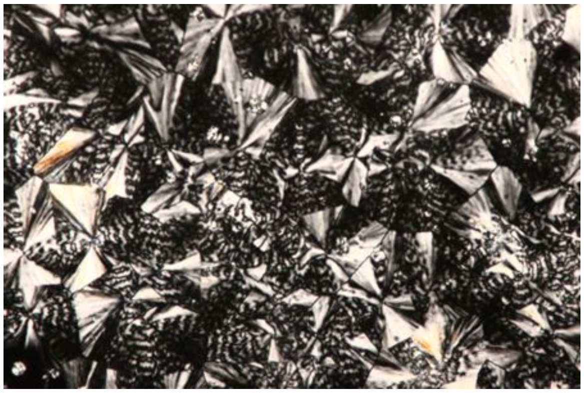
50°, T_c = 75 °C

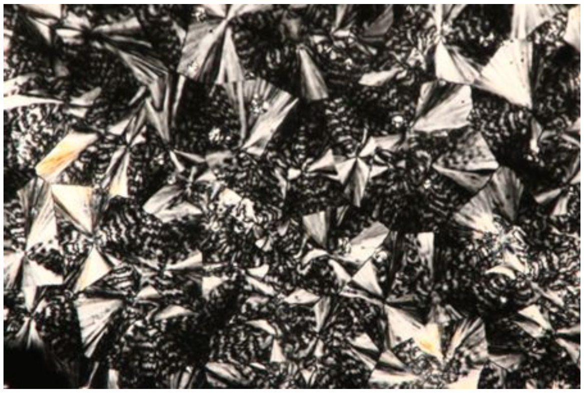

60°, T_c = 75 °C

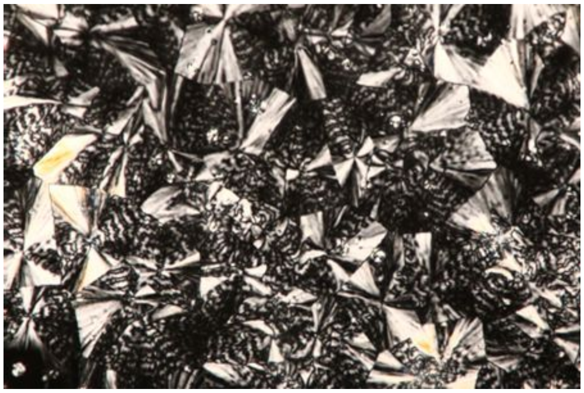

70°, T_c = 75 °C

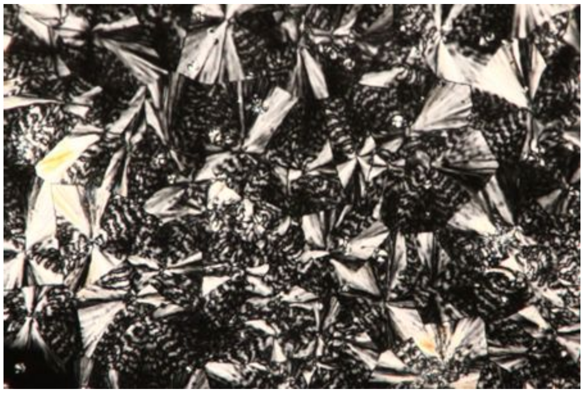

80°, T_c = 75 °C

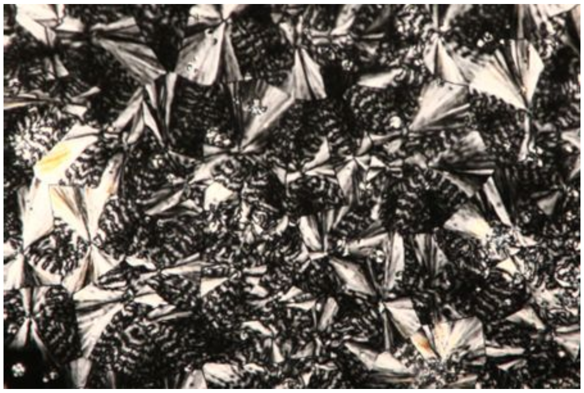

90°, T_c = 75 °C

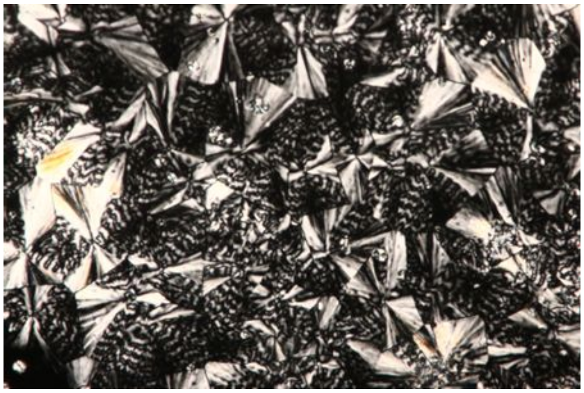

100°, T_c = 75 °C

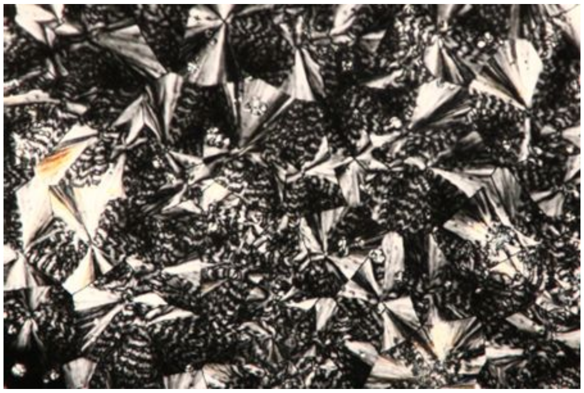

110°, T_c = 75 °C

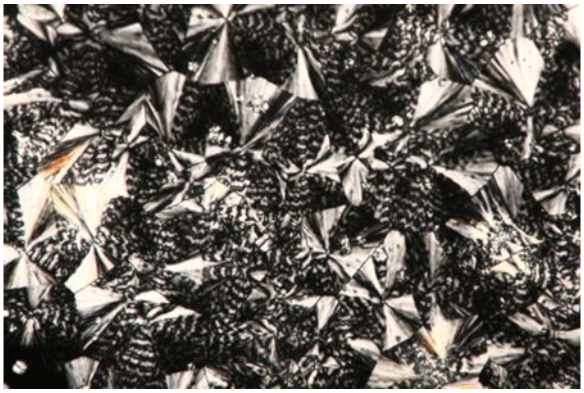

120°, T_c = 75 °C

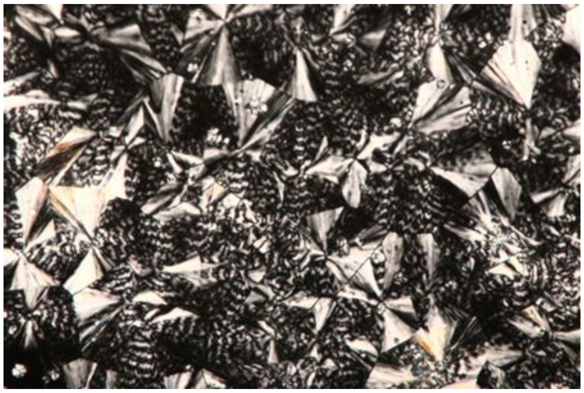

130°, T_c = 75 °C

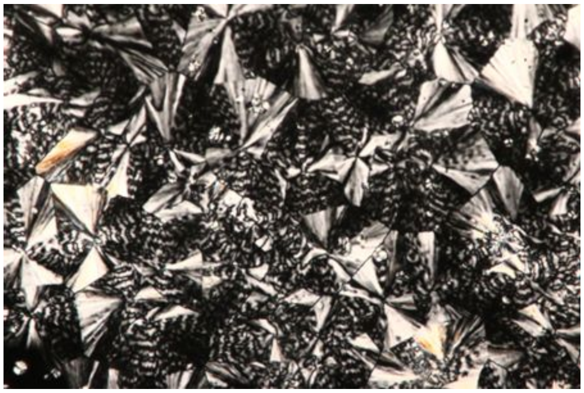

140°, T_c = 75 °C

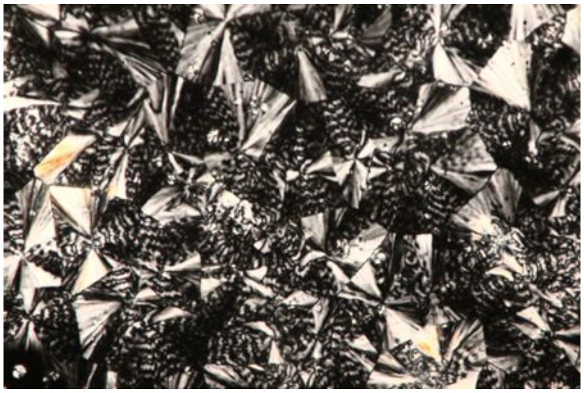

150°, T_c = 75 °C

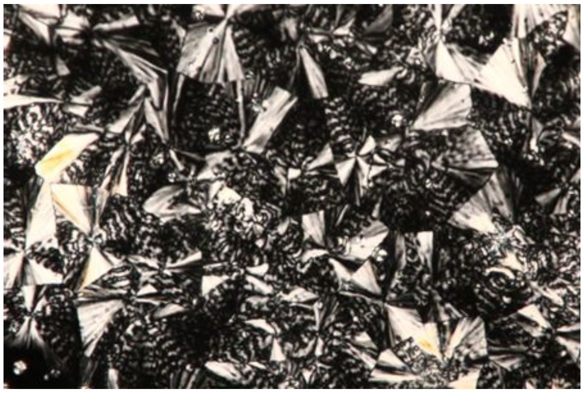

160°, T_c = 75 °C

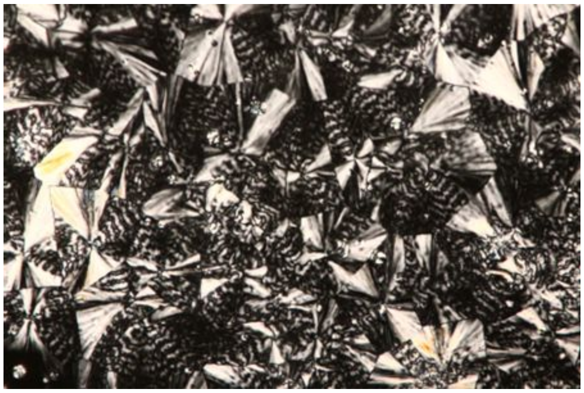

170°, T_c = 75 °C

Figure S7. PLOM images for 50/50/10 PBS/PCL/$BS_{78}CL_{22}$ sample, employing at different T_c and rotating polarizers with a fixed sample position.

Figure S7 shows images taken at 80 and 75 °C after rotating the polarizers through the indicated angles from 0° (standard position) to 180°. Experimentally, first, the polarizer is rotated to a selected position, which modifies the background color (e.g., grey instead of black if the lambda plate is not used). Next, the analyzer was rotated until a black background was obtained, indicating the polarizers' crossed position. With this operation, Figure S4 shows the Maltese cross rotated when the polarizers are rotated. The banded/non-banded regions maintain their positions and ratios between the quadrants with and without banding. This is the first evidence that the observed mixture of regions is real.

Figure S7 shows the results of (b) Rotating the sample with a fixed position of the polarizers. In this case, to facilitate sample rotation, the Linkam hot stage was not used; instead, the sample was previously thermally treated. The thermal treatment consists of a first crystallization step at 75 °C, followed by a second crystallization step at 40 °C.

Once the sample was thermally treated, it was placed at RT in the rotating stage (0 to 360°) of the Leica Microscope. Figure S5 shows images of the sample at different rotation angles. In this case, independent of the sample rotation, a fixed position of the Maltese cross is

observed (i.e., the polarizers are fixed). On the contrary, the banded and non-banded regions rotate with the sample. Independent of the degree of rotation, the ratio of two quadrants with and without banding is maintained, indicating that the novel banded/non-banded extinction pattern is real, and occurs for all spherulites within the sample.

S2.4.3. Non-isothermal PLOM experiments

Non-isothermal PLOM experiments were performed to study whether the banded/non-banded pattern is only generated during two-step crystallization. Figures S8-S11 show that the banded/non-banded pattern is present even during non-isothermal crystallization for all the ternary blends. In the case of the immiscible binary blend, Figure S8, only banded spherulites can be observed at temperatures below 90 ºC.

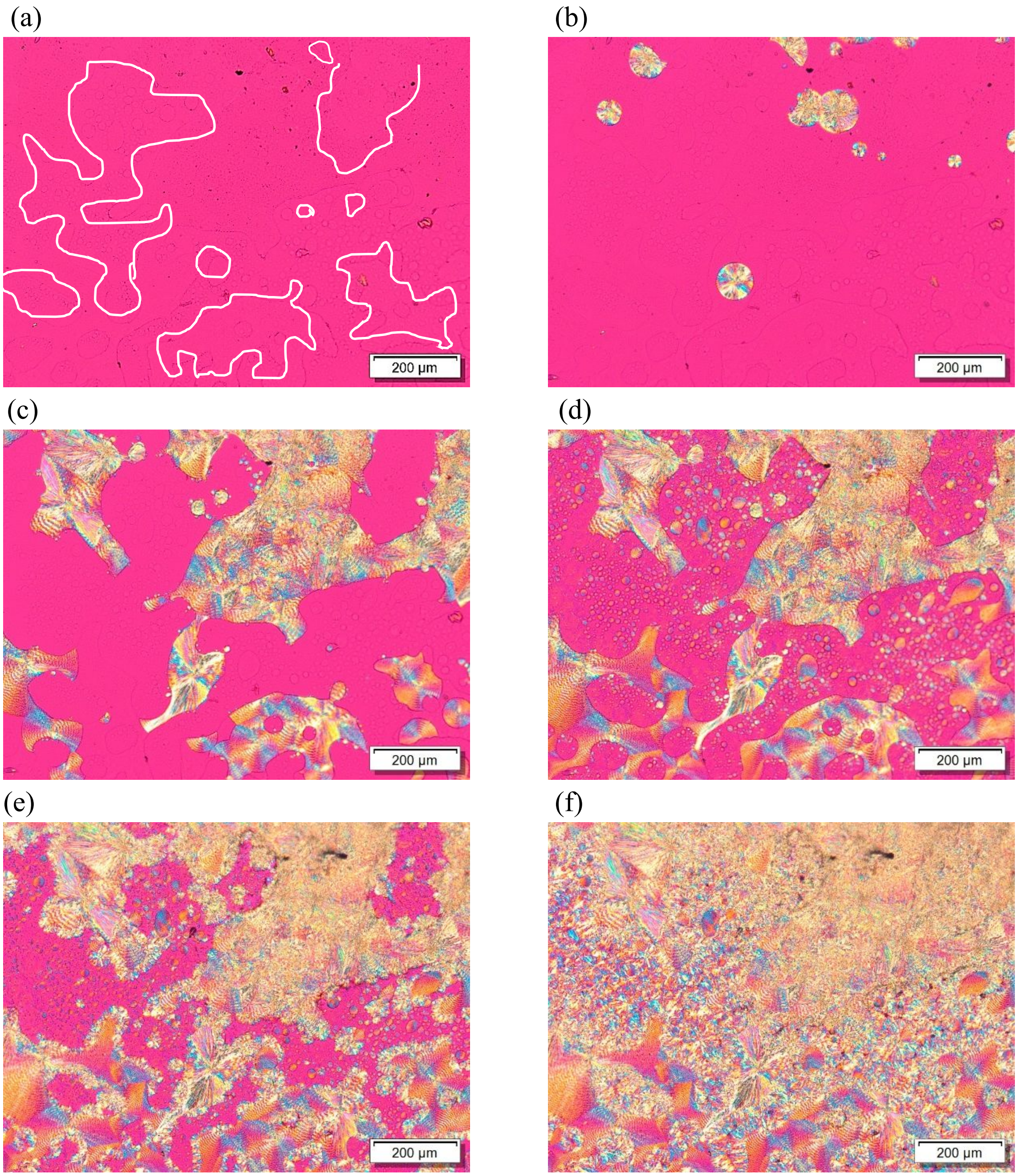

Figure S8. PLOM micrographs of PBS/PCL (50/50) sample that was cooled down from 150 ºC to 20 ºC at 10 ºC/min cooling rate. Images were taken at (a) 150 ºC, (b) 90 ºC, (c) 80 ºC, (d) 70 ºC, (e) 35 ºC, and (f) 20 ºC.

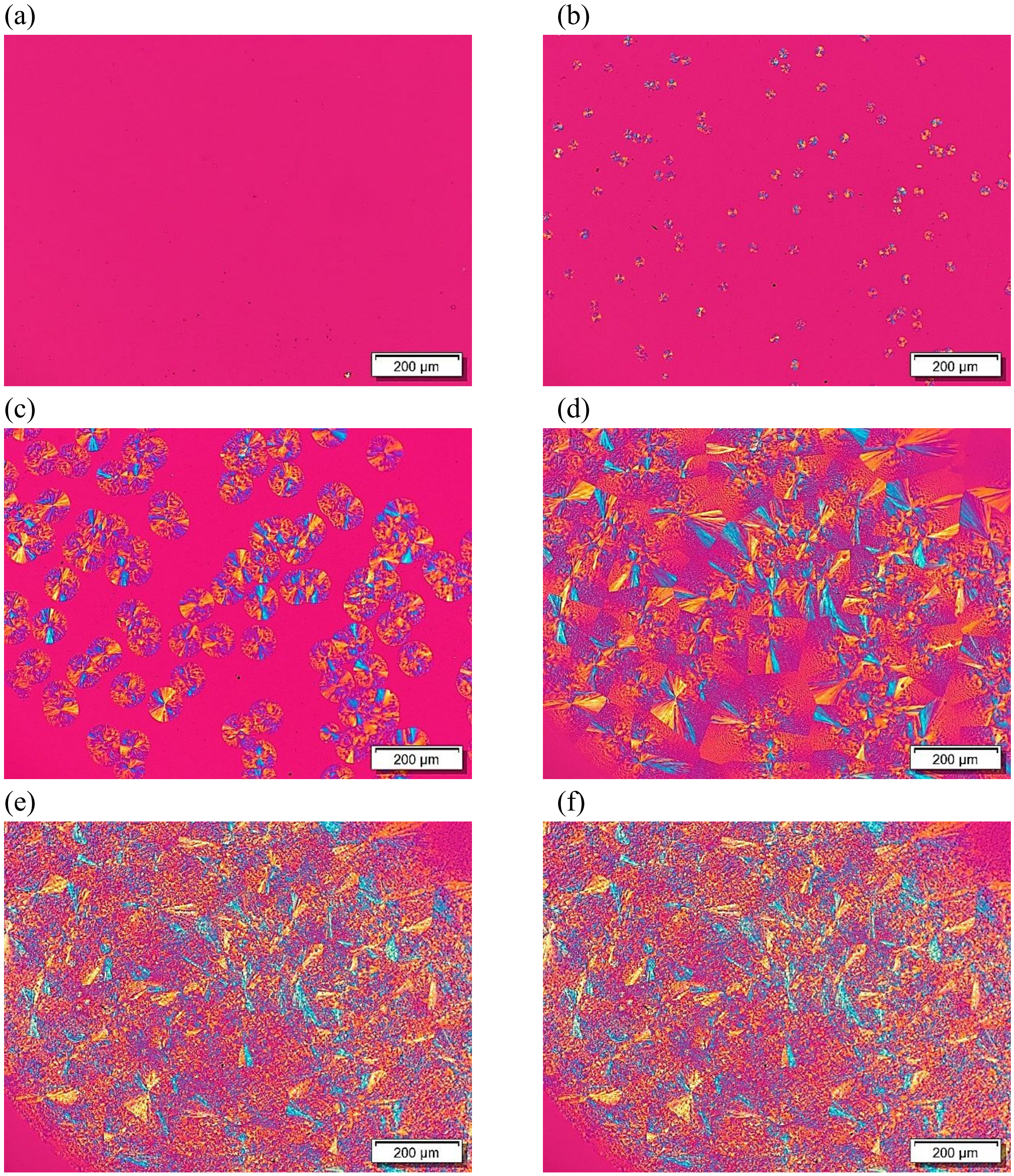

Figure S9. PLOM micrographs of PBS/PCL/$BS_{78}CL_{22}$ (50/50/10) sample that was cooled down from 150 ºC to 20 ºC at 10 ºC/min cooling rate. Images were taken at (a) 150 ºC, (b) 90 ºC, (c) 80 ºC, (d) 70 ºC, (e) 30 ºC, and (f) 20 ºC.

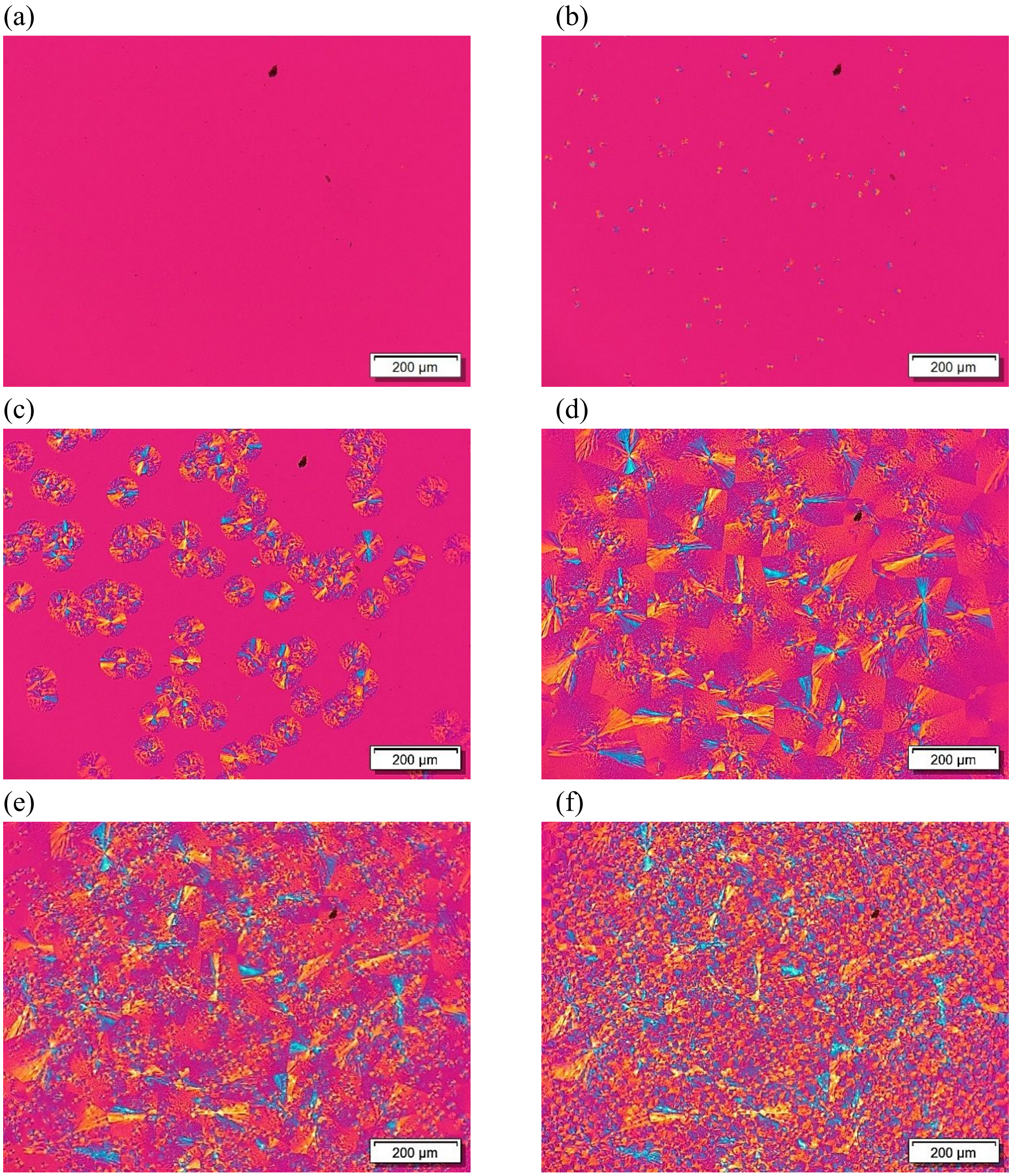

Figure S10. PLOM micrographs of PBS/PCL/$BS_{46}CL_{54}$ (50/50/10) sample that was cooled down from 150 ºC to 20 ºC at 10 ºC/min cooling rate. Images were taken at (a) 150 ºC, (b) 90 ºC, (c) 80 ºC, (d) 70 ºC, (e) 30 ºC, and (f) 20 ºC.

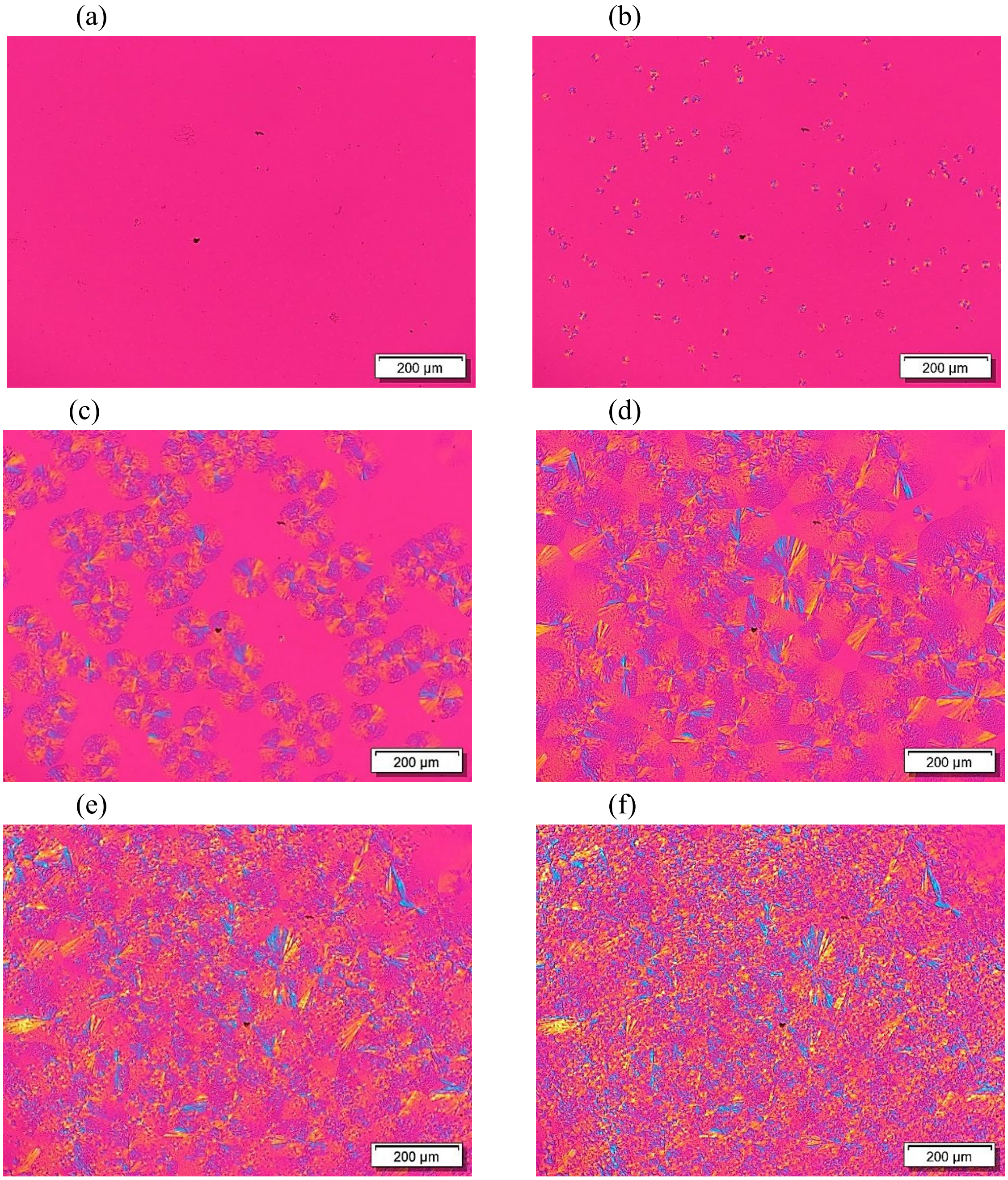

Figure S11. PLOM micrographs of PBS/PCL/$BS_{15}CL_{85}$ (50/50/10) sample that was cooled down from 150 ºC to 20 ºC at 10 ºC/min cooling rate. Images were taken at (a) 150 ºC, (b) 90 ºC, (c) 80 ºC, (d) 70 ºC, (e) 30 ºC, and (f) 20 ºC.

2.4.4. Two-step Non-isothermal PLOM experiments

Morphological studies were conducted with PLOM, utilizing a two-step crystallization process to further investigate the thermal transitions. Figure S5 illustrates the PCL (S5a) and PBS (S5b) spherulites after isothermal crystallization at 40 ºC and 85 ºC, respectively.

Figure 12a shows the phase separation resulting from the immiscibility of the 50/50 PBS/PCL binary blend in the molten state, as indicated by the white lines. Figures 12b to 12d present the morphology at the isothermal temperature (T_c) of 85 °C after crystallization from the melt at different times: 1 minute (12b), 5 minutes (12c), and 10 minutes (12d). At T_c = 85 °C, only the PBS phase can crystallize, leading to the formation of non-banded spherulites. After 10 minutes, the PBS phase is fully crystallized while the PCL phase remains molten (see the pink background in Figure 4c). Once the PBS phase crystallized at T_c = 85 °C, the blend sample was cooled to 40 °C and held for 5 minutes to allow the PCL phase to crystallize. The PCL formed small spherulites nucleated by the pre-existing crystalline structures from the PBS phase. The PCL was crystallized to saturation after 5 minutes at 40 °C (Figure 12f). Figure S12 shows that the PBS and PCL phases in the blend crystallized independently, each forming its own supercrystalline structure due to macrophase separation.

The two-step crystallization protocol used in the binary blends was also applied to the ternary blends. The effect of adding BS_xCL_y copolymers was analyzed in Figures S13 to 15, revealing that the blend morphology is influenced by the BS_xCL_y composition.

Figure 13 illustrates how the morphology of the 50/50 PBS/PCL blends changes with the addition of the $BS_{78}CL_{22}$ copolymer. Figure 13a shows the absence of separated domains in the molten state, at least at this scale, indicating that the addition of $BS_{78}CL_{22}$ enhances the miscibility of the blend in the melt state. Figures 13b to 13d depict the crystallization of the PBS phase at T_c = 85 °C, featuring spherulitic structures. Compared to the binary blend (see Figure 12), the observed spherulites are smaller yet compact, exhibiting a higher nucleation density. This is attributed to enhanced crystallization resulting from the co-crystallization of the PBS phase with the BS fraction, consistent with the DSC results (see Figure 1).

Interestingly, the spherulites in the ternary blends exhibit a unique combination of banded and non-banded regions (two quadrants with banding and two without), which may suggest miscibility or partial miscibility (without macroscopic phase segregation at the observed microscopic scale).

PLOM experiments were conducted under non-isothermal conditions for all 50/50/10 PBS/PCL/BSxCLy blends (see Figures S13 to S15), revealing the unique mixture of banded and non-banded regions in the spherulites. This morphology resembles the Janus-face spherulites discussed below. It is important to note that in the 50/50 PBS/PCL binary blend, PBS spherulites exhibited complete ringed patterns when formed through non-

isothermal crystallization (Figure S12). However, when PBS crystallized via two-step isothermal crystallization (Figure 4), the PBS spherulites displayed only a non-banded morphology.

The authenticity of this peculiar extinction pattern was verified using various setups. We performed an experiment in which the polarizers were rotated while remaining crossed, with the sample position fixed. The polarizers' rotation from 0 to 180° demonstrates that the same ratio of two quadrants with and without banding is preserved, regardless of the polarizers' rotation, as shown by the Maltese Cross pattern (see Figure S4). A second experiment involved rotating the sample from 0 to 360° with the polarizers fixed. In this instance, the Maltese Cross pattern consistently remains in the same position, while the banded and non-banded regions rotate. Despite the rotation, the ratio of banded to non-banded regions remained consistent across all rotations (see Figure S4). This confirms the pattern's presence throughout the entire 3D structure of the spherulite, thereby establishing that the extinction pattern is authentic.

Further studies on the banded region were conducted by crystallizing the sample at various T_c. A temperature-dependent behavior of the banded region was observed, evolving from clear, well-defined bands (with a short banding spacing) at low T_c to diffuse bands (with a wider banding spacing) at T_c as high as 100 °C. The banded/non-banded pattern remains independent of T_c.

Nurkhamidah et al.[13] reported that spherulite morphology with a Janus-face (dendritic and ring-banded regions) in PBS/PEO blends forms under specific conditions. These structures emerge at intermediate PEO contents (50/50-80/20 wt.%) at a fixed T_c of 70°C. Detailed SEM and PLOM analyses reveal a dual-layer nuclei geometry, with dendritic lamellae forming first, followed by bent sheaf crystals that create ring-banded patterns. Additionally, they note that top-free surfaces are essential for dual-face development, emphasizing the importance of nucleation and lamellar assembly.

In our study, PLOM tests were conducted on samples prepared using both solution and melt methods, with and without a top cover, leading to variations in thickness and confinement. However, no differences in morphology were observed among samples with varying thickness. Therefore, we conclude that the observed morphology is not influenced by the confinement of the samples between glass slides, which differs from the findings reported by Nukhamidah et al.[13] Furthermore, Nukhamidah et al. offered deeper insights into the formation mechanisms of Janus-face spherulites by analyzing SEM and AFM. They stated that these unique spherulites consist of two distinct regions that share a common nucleus but grow independently and simultaneously.

Figures S13e-f show the crystallization of the PCL phase at T_c = 40 °C. It occurs preferentially in empty domains and extends to all the observed areas, indicating a similar mechanism for the different ternary blends. The PCL phase spherulites were smaller and

had a higher nucleation density than those in the binary blend. This phenomenon is attributed to the nucleation effect of the enhanced PBS phase on the PCL phase, consistent with the DSC results (Figure 1).

Figure S14 presents a notably different scenario for the addition of $BS_{46}CL_{54}$ copolymer. On one hand, PBS crystallizes from a homogeneous melt with a similar spherulite size (in the early stages) and nucleation density compared to the blends with $BS_{78}CL_{22}$ (see Figures S12). On the other hand, the qualitative growth of PBS spherulites is considerably slower; even after extended durations, the PBS spherulites in the $BS_{46}CL_{54}$ blend remain smaller than those in the $BS_{78}CL_{22}$. Moreover, despite exhibiting a mixture of banded and non-banded regions, the ringed pattern is diffuse while utilizing the same T_c, indicating that the molten PCL phase has minimal impact as a diluent due to inadequate compatibilization. Figures S14e-14f illustrate that the PCL phase spherulites are larger and more distinct, showcasing a reduced nucleation effect of the PBS phase on the PCL phase. Overall, the PBS/PCL/$BS_{46}CL_{54}$ blends demonstrate decreased miscibility.

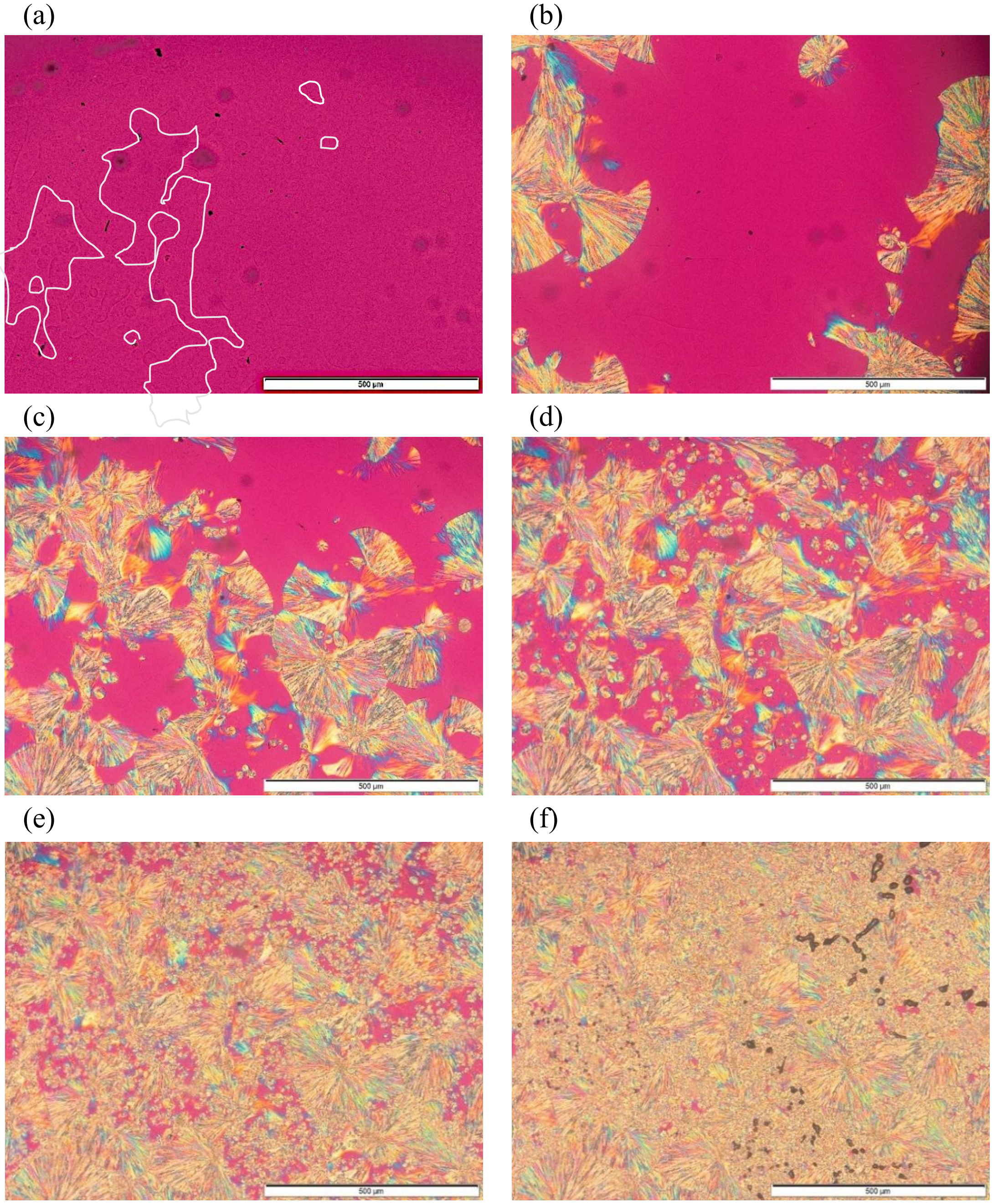

Figure S12. PLOM micrographs of PBS/PCL (50/50) sample that was cooled down from 150 ºC (a) to 85 ºC and held at this temperature for (b) 1 min, (c) 5 min, and (d) 10 min. Then the sample was cooled to 40 ºC and held at this temperature for 1 min (e), and 5min (f). The scale bar is 500 µm.

(a) (b) (c) (d) (e) (f)

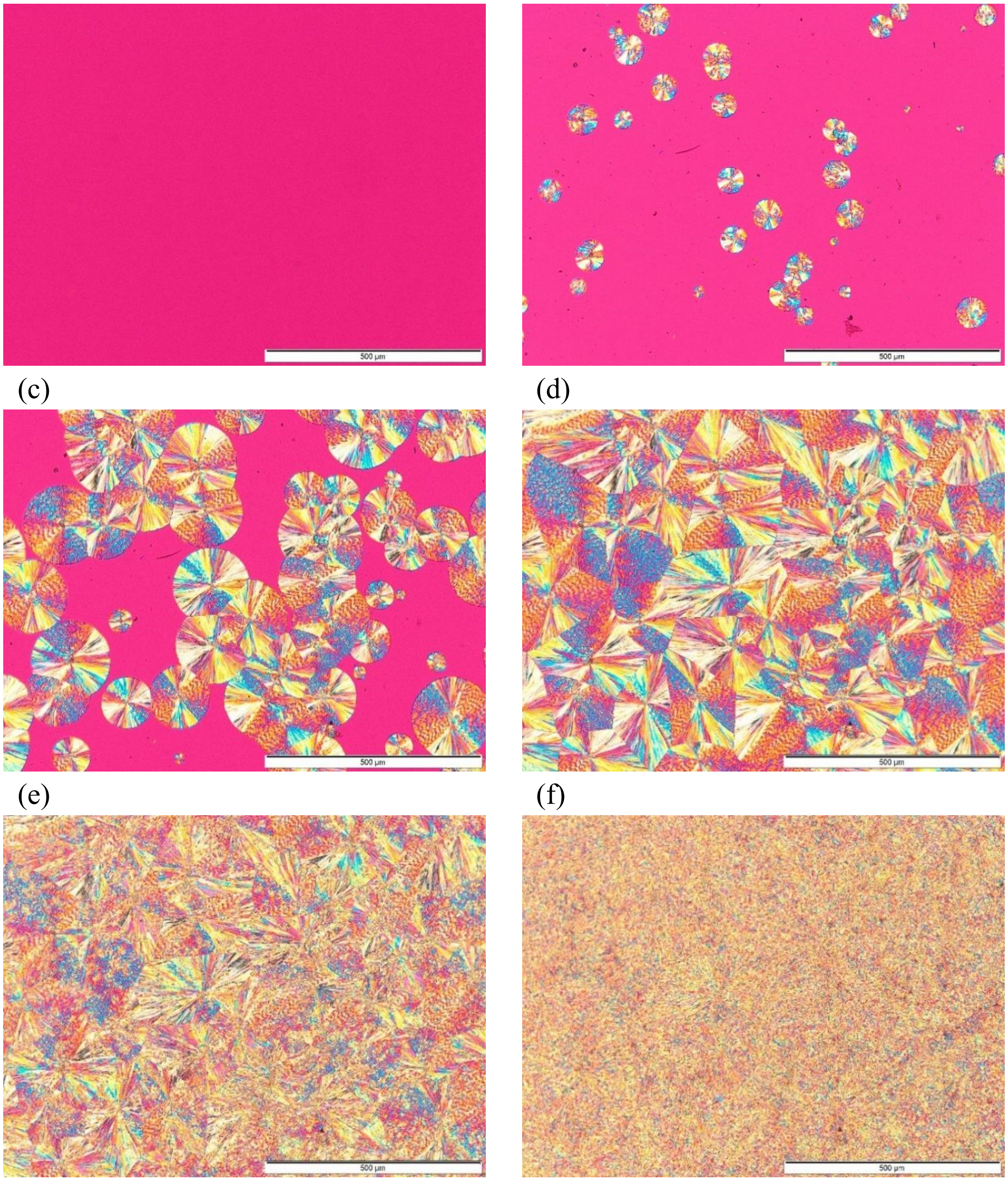

Figure S13. PLOM micrographs of PBS/PCL/$BS_{78}CL_{22}$ (50/50/10) sample that was cooled down from 150 ºC (a) to 85 ºC and held at this temperature for (b) 1 min, (c) 5 min, and (d) 10 min. Then the sample was cooled down to 40 ºC and held at this temperature for (e) 1 min, and (f) 5 min. The scale bar is 500 µm.

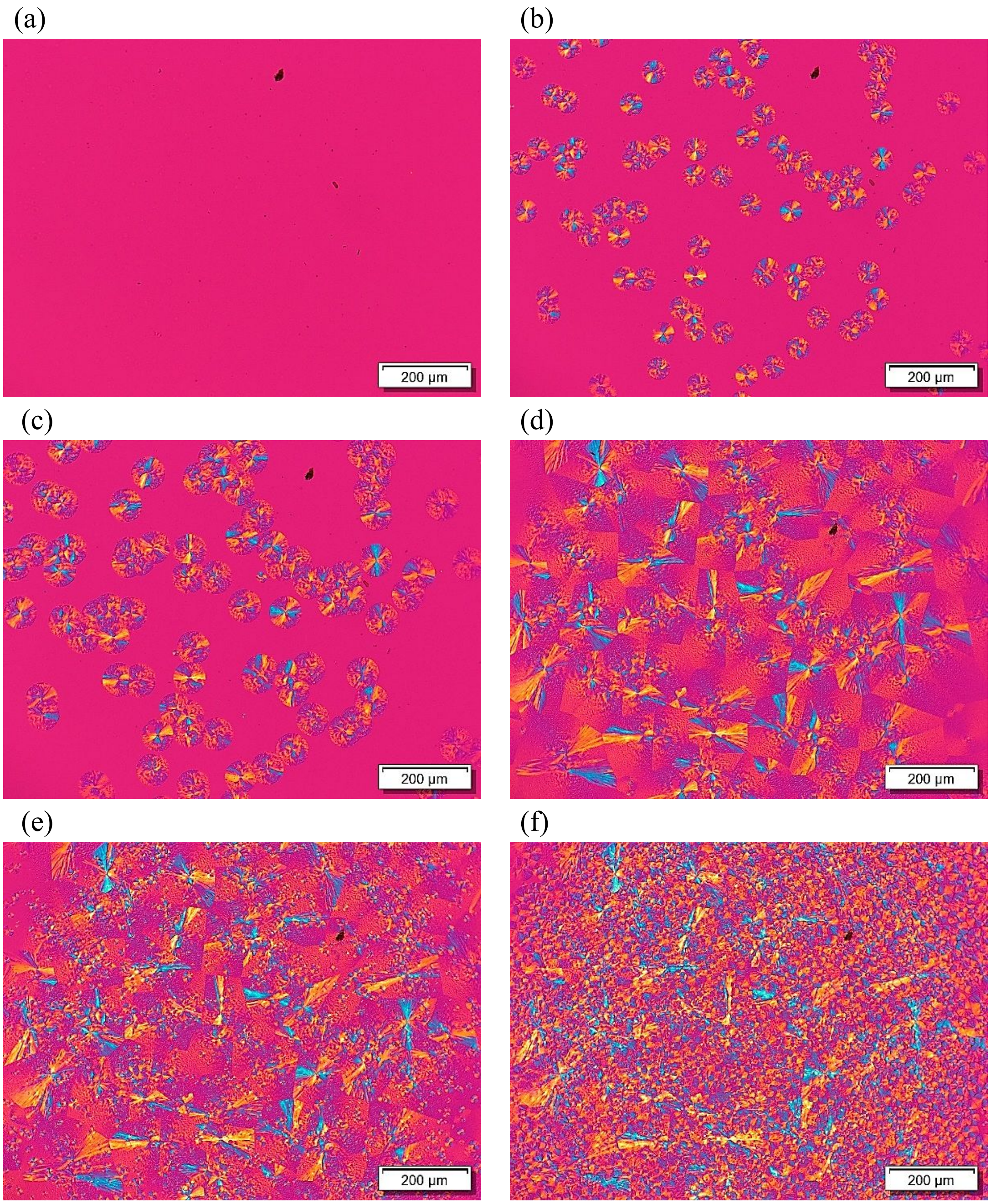

Figure S14. PLOM micrographs of PBS/PCL/$BS_{46}CL_{54}$ (50/50/10) sample that was cooled down from 150 ºC (a) to 85 ºC and held at this temperature for (b) 2 min, (c) 10 min. Then the sample was cooled down to 40 ºC and held at this temperature for (d) 30 sec, (e) 1 min and (f) 5 min. The scale bar is 200 µm.

Figure S15 illustrates the impact of the $BS_{15}CL_{85}$ addition on the morphology of the PBS/PCL blend. In the molten state, Figure S15a shows phase separation (at the measured scale), with domains smaller than those in the binary blend (Figure 4a), suggesting a certain compatibilizer effect. In the solid state, Figures S15b-d show that the PBS phase's

spherulites are smaller, banded, and denser than those in the binary blend. The $BS_{15}CL_{85}$ addition also creates a distinct mixture of banded and non-banded regions within the spherulites. Considering the larger spherulitic size and lower nucleation density compared to the $BS_{78}CL_{22}$ addition, the $BS_{15}CL_{85}$ addition results in lower miscibility.

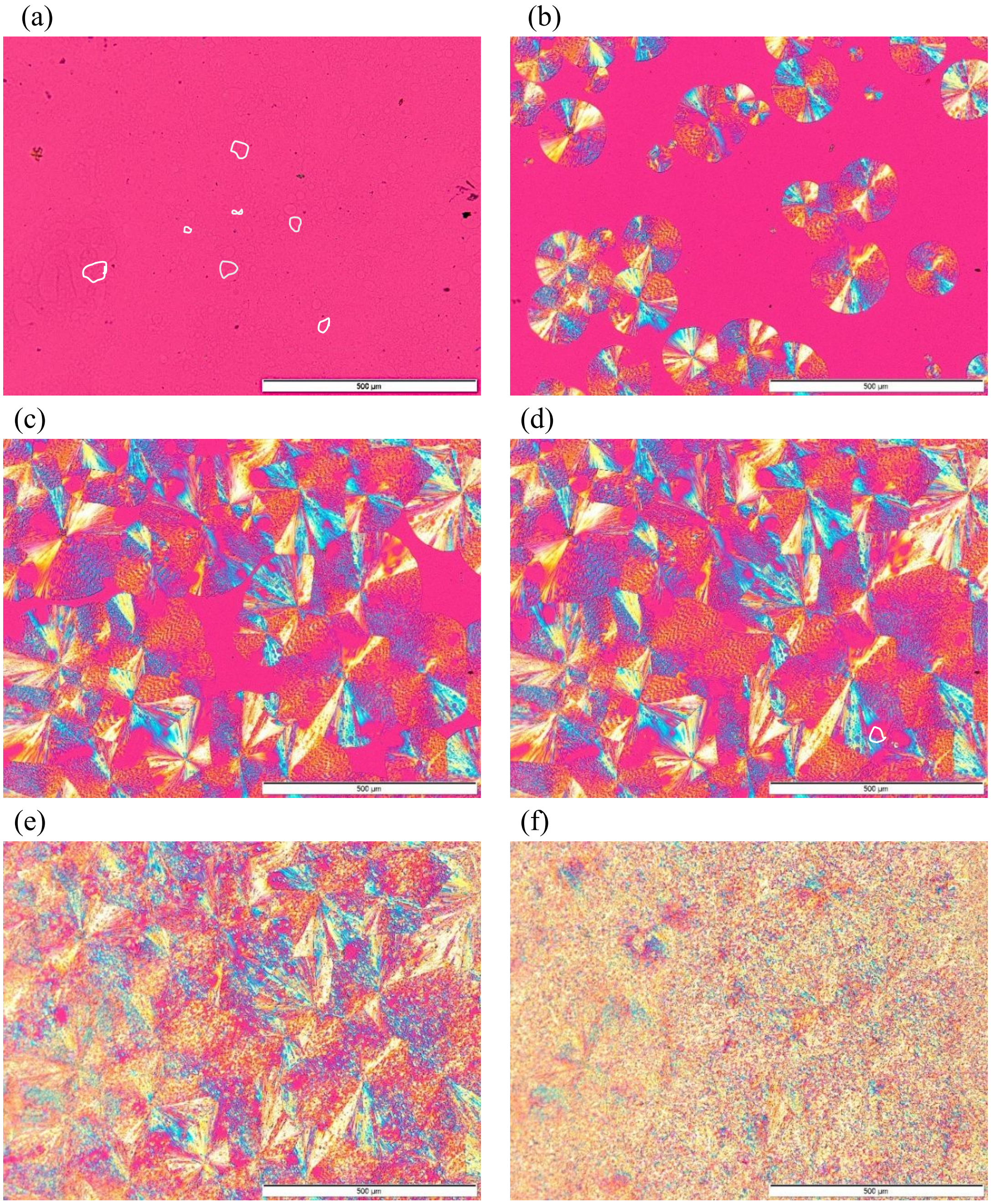

Figure S15. PLOM micrographs of PBS/PCL/$BS_{15}CL_{85}$ (50/50/10) sample that was cooled down from 150 ºC (a) to 85 ºC and held at this temperature for (b) 2 min, (c) 5 min, and (d) 10 min. Then the sample was cooled down to 40 ºC and held at this temperature for (e) 1 min, and (f) 5 min. The scale bar is 500 µm.

S2.5. FT-IR studies of PBS in banded and non-banded areas

The FT-IR spectra of the PBS/PCL/$BS_{78}CL_{22}$ blend (Figure S16) were collected from selected non-banded and banded regions within individual spherulites to resolve local chemical and conformational variations. Figure 16a–c show spectra from both regions in the ranges 3050–2800 cm^{-1}, 1500–700 cm^{-1}, and 910–990 cm^{-1}, respectively. Polarized FT-IR measurements, Figure S17, acquired after holding the sample for 20 min at 85 °C further highlight differences in molecular orientation between the non-banded and banded zones across the full 4000–700 cm^{-1} spectral window. Additional spectra from the non-banded region in the 1500–900 cm^{-1} and 1800–1650 cm^{-1} ranges provide higher-resolution insights into the vibrational modes sensitive to crystallinity and segmental ordering in the multicomponent blend.

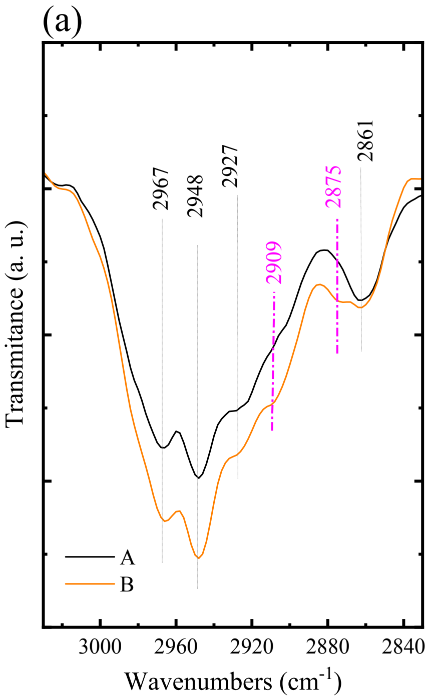

(b)

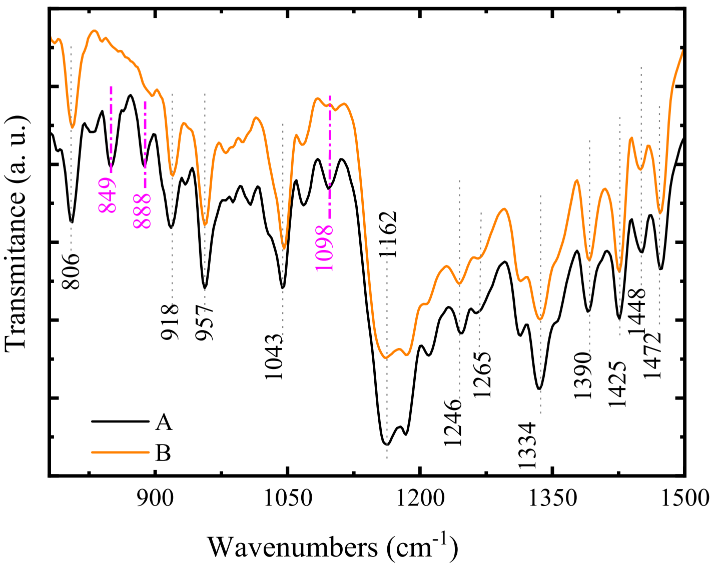

(c)

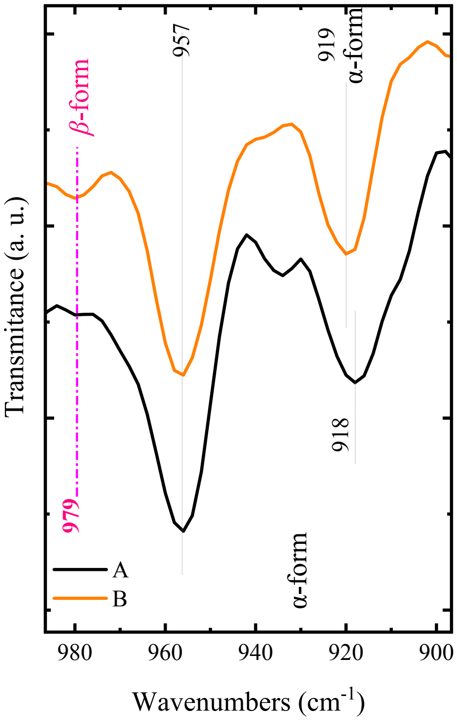

Figure S16: FT-IR spectra of PBS/PCL/$BS_{78}CL_{22}$ blend sample at specific non-banded (A) and banded (B) regions at 3050-2800 cm^{-1} (a), 1500-700 cm^{-1} (b) and 910-990 cm^{-1} (c).

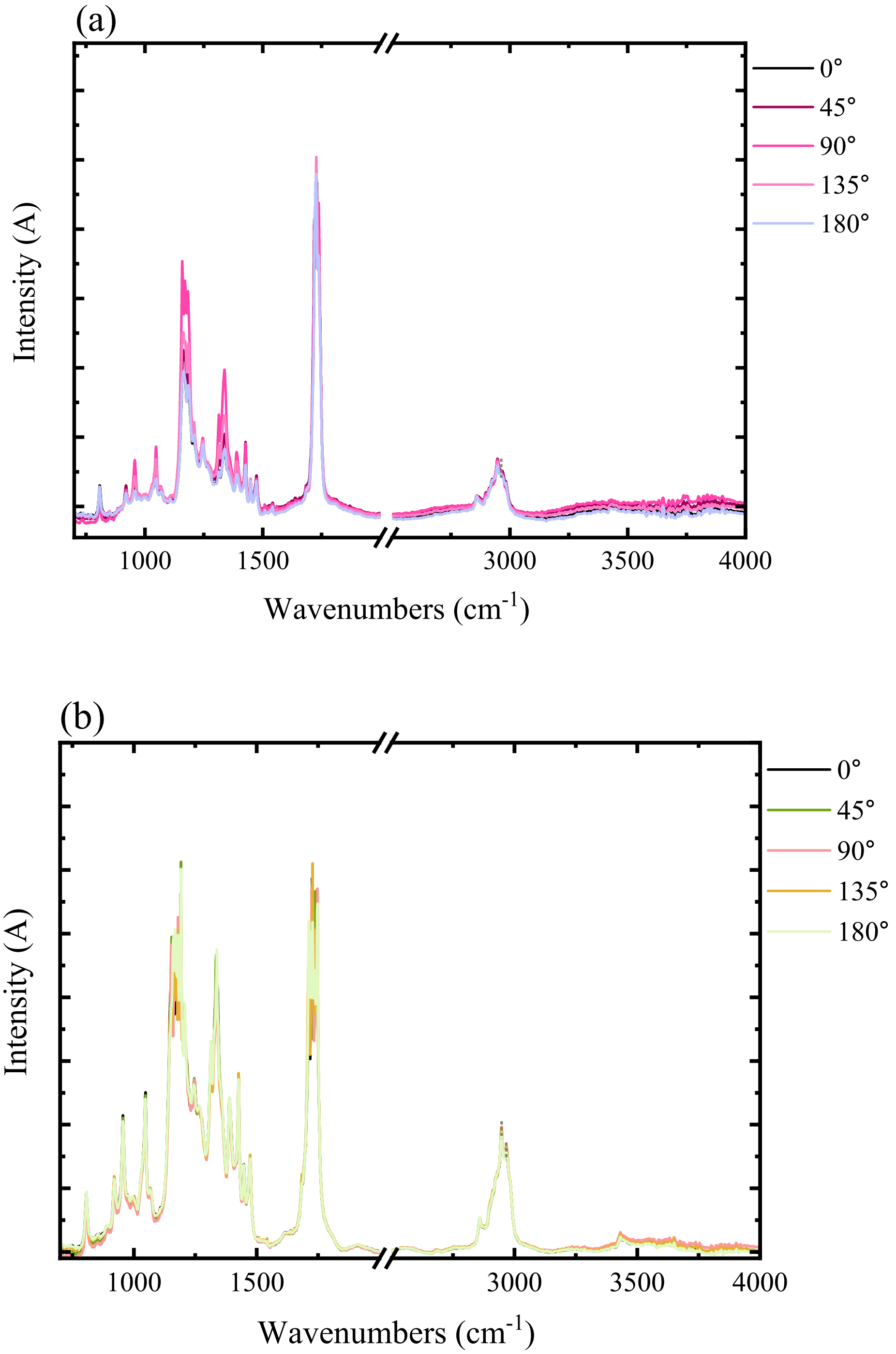
(a)
Intensity (A)
Wavenumbers (cm-1)
1000
1500
3000
3500
4000
0°
45°
90°
135°
180°
(b)
Intensity (A)
Wavenumbers (cm-1)
1000
1500
3000
3500
4000
0°
45°
90°
135°
180°

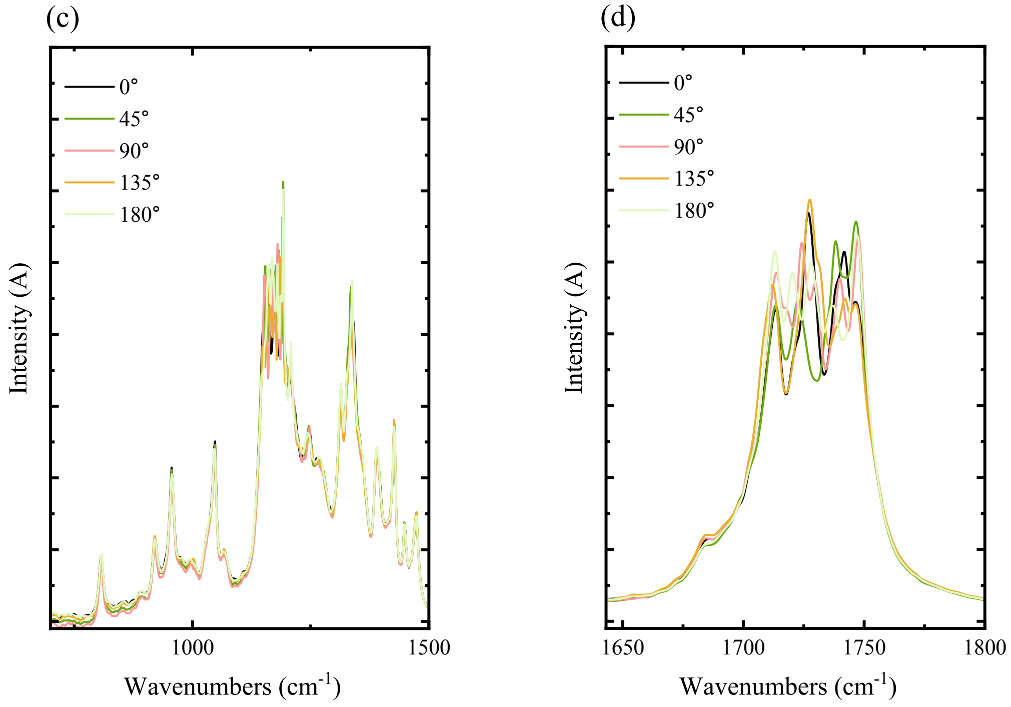

Figure S17. Polarized FT-IR spectra after holding 20 min at 85 ºC for the indicated non-banded (a) and banded (b) region in a single spherulite for PBS/PCL/$BS_{78}CL_{22}$ sample in the full spectral range, from 4000 to 700 cm^{-1}. FT-IR spectra of PBS/PCL/$BS_{78}CL_{22}$ blend sample at specific non-banded (A) region at 1500-900 cm^{-1} (c), 1800-1650 cm^{-1} (d).

S2.6. WAXD/SAXS Experiments

In-situ simultaneous WAXD/SAXS experiments were conducted on all the materials. The patterns were recorded during cooling (from the molten state) and heating scans at a scan rate of 20 °C/min. First, each component of the ternary blend was individually characterized.

S.2.6.1. Homopolymers

The WAXD patterns of the PCL, see Figures S18a, showed sharp peaks related to the PCL main reflections at q = 15.3 nm^{-1} and q = 17.0 nm^{-1}, and a shoulder at q = 15.7 nm^{-1} assigned to (110), (200), and (111) crystal planes, respectively. These reflections correspond to the orthorhombic unit cell of the PCL, with a = 0.748, b = 0.498, and c = 1.726 nm.[14, 15] For the PBS, the WAXD patterns (see Figures S19c and S19d) exhibited strong intensity reflections at q = 14.1 nm^{-1} and 16.3 nm^{-1}, and medium intense reflections at q = 15.7 nm^{-1} and 20.3 nm^{-1} (not shown). These reflections were assigned to the (020), (110), (021), and (111) crystal planes, respectively. They correspond to the main

reflections of the monoclinic unit cell of the α-form PBS, with a = 0.532, b = 0.912, c = 1.090 nm, and β = 123.9°.[16, 17] It is worth noting that the PBS can also crystallize under stretching,[17] forming β-form crystals (monoclinic, with a = 0.584, b = 0.832, c = 1.186 nm, and β = 131.6°).

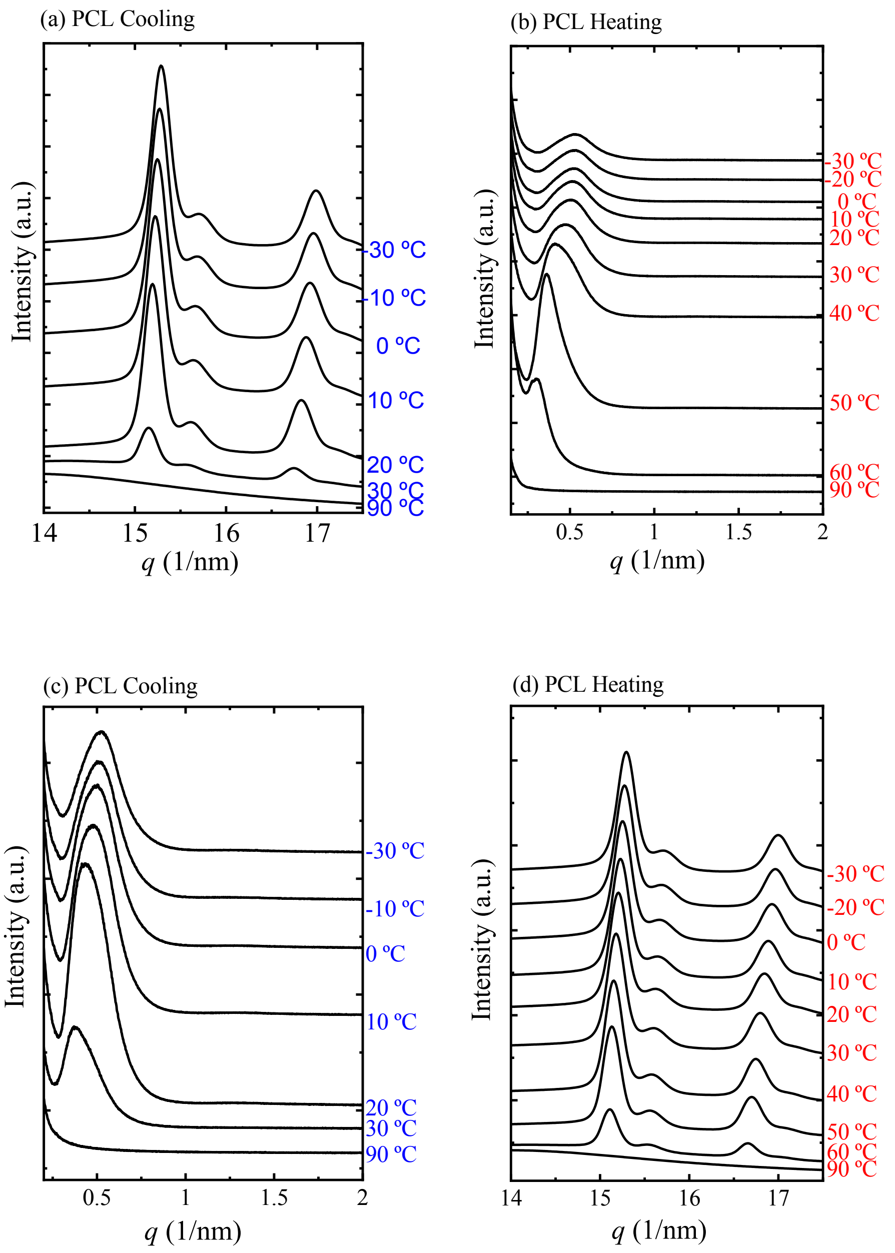

Figure S18. *In-situ* SAXS (a-b) and WAXD (c-d) profiles of the neat PCL during cooling/heating runs at a rate of 20 ºC/min.

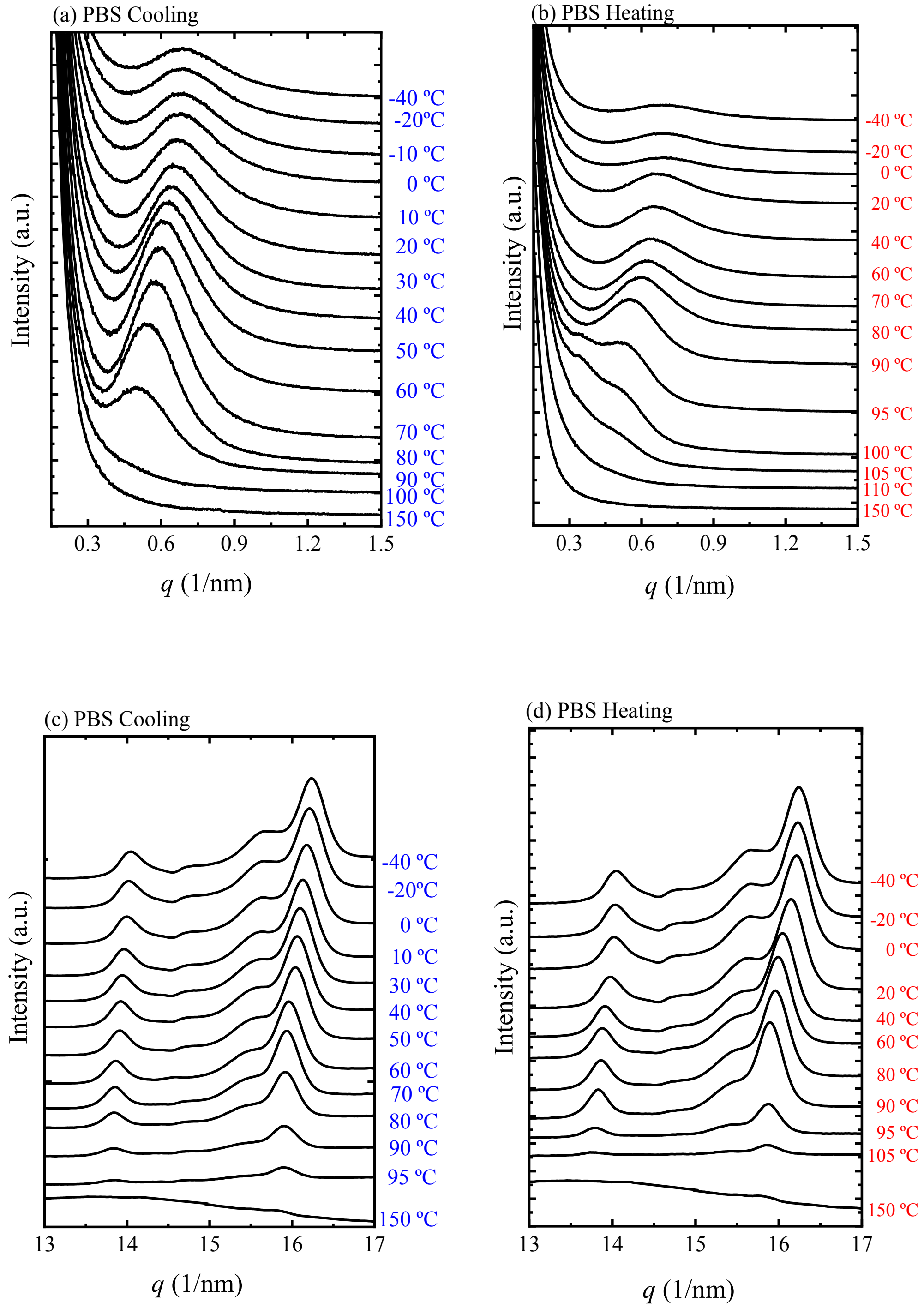

Figure S19. In-situ SAXS (a-b) and WAXD (c-d) profiles of the neat PBS during cooling/heating runs at a rate of 20 ºC/min.

S2.6.2. Binary and Ternary Blends: PBS/PCL Blends with Random Copolymers as Compatibilizing Agents

The 50/50 PBS/PCL blends exhibit the main reflections of both PBS and PCL without significant variation; see Figure S21a. The calculated inter-planar distances, *d*-spacings, remain unchanged, whereas lower long periods values were found in the 50/50 PBS/PCL blends compared with the neat materials. The results are listed in Table S2.

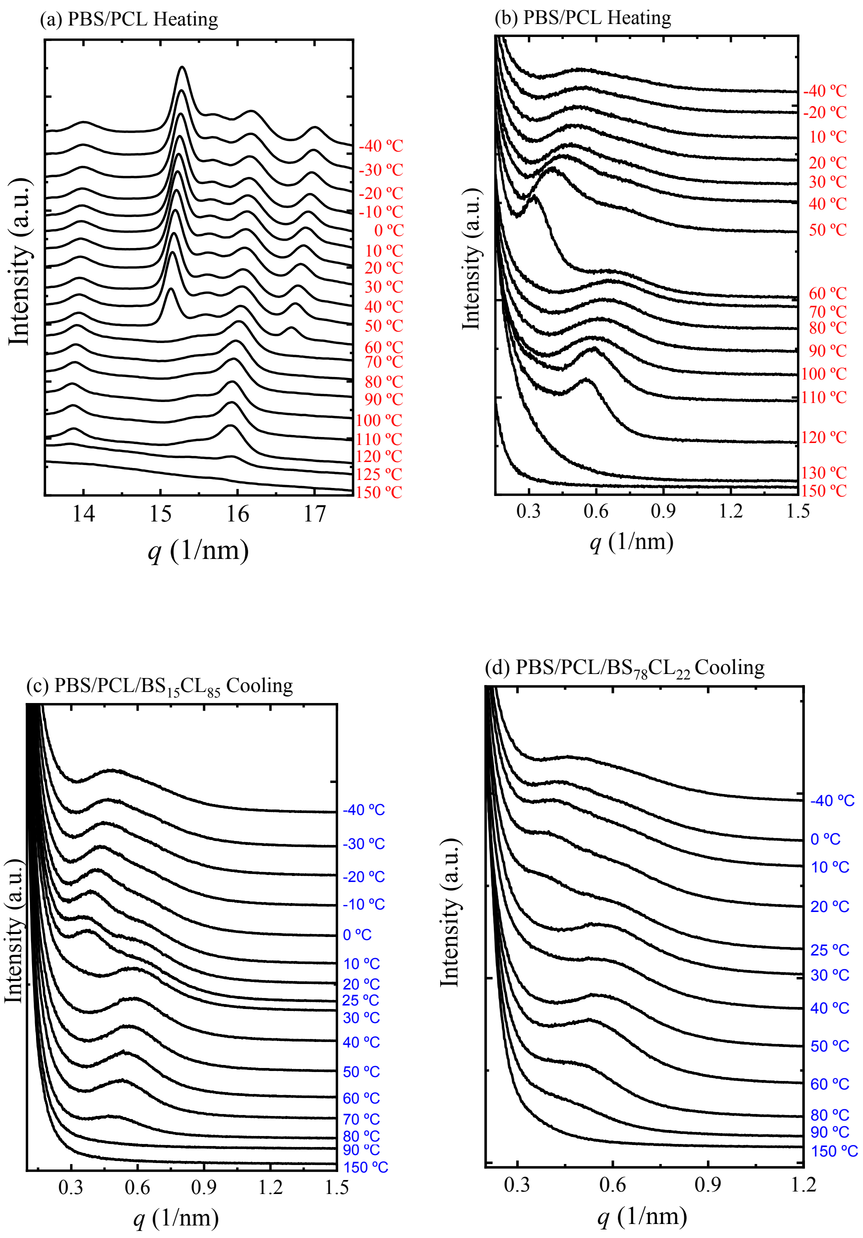

Figure S20. WAXD (a) and SAXS (b) of PBS/PCL 50/50 sample during the melting at a rate of 20 ºC/min. (c) SAXS of PBS/PCL/$BS_{15}CL_{85}$ sample during cooling at a rate of 20 ºC/min. (c) SAXS of PBS/PCL/$BS_{78}CL_{22}$ sample during cooling at a rate of 20 ºC/min.

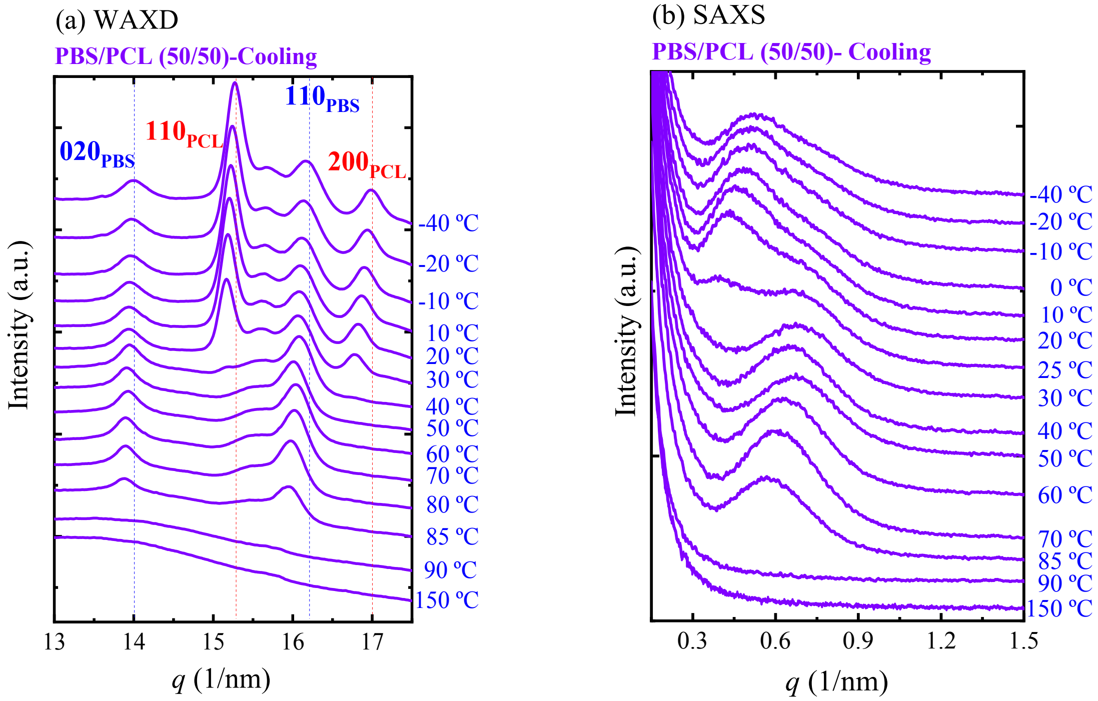

Figure S21. WAXD (a) and SAXS (b) of PBS/PCL (50/50) during the dynamic crystallization at a cooling rate of 20 ºC/min.

In Figure S21b, the SAXS patterns observed in the molten state for binary blends showed similar features, suggesting that the immiscible nature in the molten state is preserved.

For the solid state, the WAXD and SAXS patterns of the 50/50 PBS/PCL blends, measured during the cooling from the melt (Figure S21a) confirmed the distinct and independent crystallization of the PBS and PCL components of the binary blend, similar to the PLOM results. In Figure S21a, first, the PBS crystallizes (see the reflections from the indicated (020) and (110) PBS planes) at approximately 85 °C, followed by the PCL (see the reflections from the indicated (110) and (200) PCL planes) at around 30 °C. Figure S21b shows the evolution of the SAXS profiles. As expected, at 85 °C, the single peak corresponds to the PBS phase. Then, the PCL forms as secondary lamellae at random locations around 30 °C. These secondary PCL lamellae are thicker than the primary PBS lamellae (d^* values of 16.5 nm vs. 10.8 nm). From 25 °C to about 0 °C, well-defined double scattering peaks appear; however, at temperatures below 0 ºC, a sharp PCL peak overlaps the PBS peak. According to the dual lamellar stack model, PCL lamellae are primarily located in individual stacks, separate from the primarily formed PBS stacks.[18]

The BS_xCL_y copolymers were characterized in previous works.[2, 3, 19] Briefly, the $BS_{78}CL_{22}$ and $BS_{15}CL_{85}$ exhibited WAXD patterns similar to those of PBS and PCL, respectively, with

a slight shift in the q positions due to the inclusion of comonomers. The calculated d-spacings are higher than those of the parent components, reflecting changes in the unit cell due to comonomer inclusion, as expected for isodimorphic copolymers, see Table S4. In the case of the $BS_{46}CL_{54}$, situated at the pseudo-eutectic point, both comonomers can crystallize at higher temperatures for the BS fraction and at lower temperatures for the CL fraction, resulting in significantly shifted q positions and, consequently, higher d-spacings compared to the neat materials.

Table S4. Data extracted from in-situ WASD and SAXS profiles at -40 ºC for the indicated samples.

Sample	From SAXS profile		From WAXD profile	
	q (1/nm)	L_p (nm)	q (1/nm)	*d spacing* (nm)
Neat PBS	0.689	9.12	14.05, 16.25	0.447, 0.386
Neat PCL	0.522	12.04	15.28, 16.99	0.411, 0.370
PBS/PCL 50/50	0.542	11.58	13.99, 16.10<br>15.27, 17.01	0.449,0.390<br>0.411, 0.396
PBS/PCL/$BS_{78}CL_{22}$ 50/50/10	0.460	13.65	13.89, 16.02<br>15.15, 16.83	0.452, 0.393<br>0.414, 0.374

S2.7. Nanobeam X-ray imaging

The intensity maps of the 110 $_{PBS}$, 201 $_{PBS}$, 020 $_{PBS}$, 200 $_{PCL}$, and 110 $_{PCL}$ reflections (Figure S22) clearly resolve the spatial distribution of crystalline orientations within the growing spherulites. The solid white lines highlight the impingement boundaries between neighboring spherulites, serving as visual guides to delineate regions where growth fronts collided. See the main text for more details.

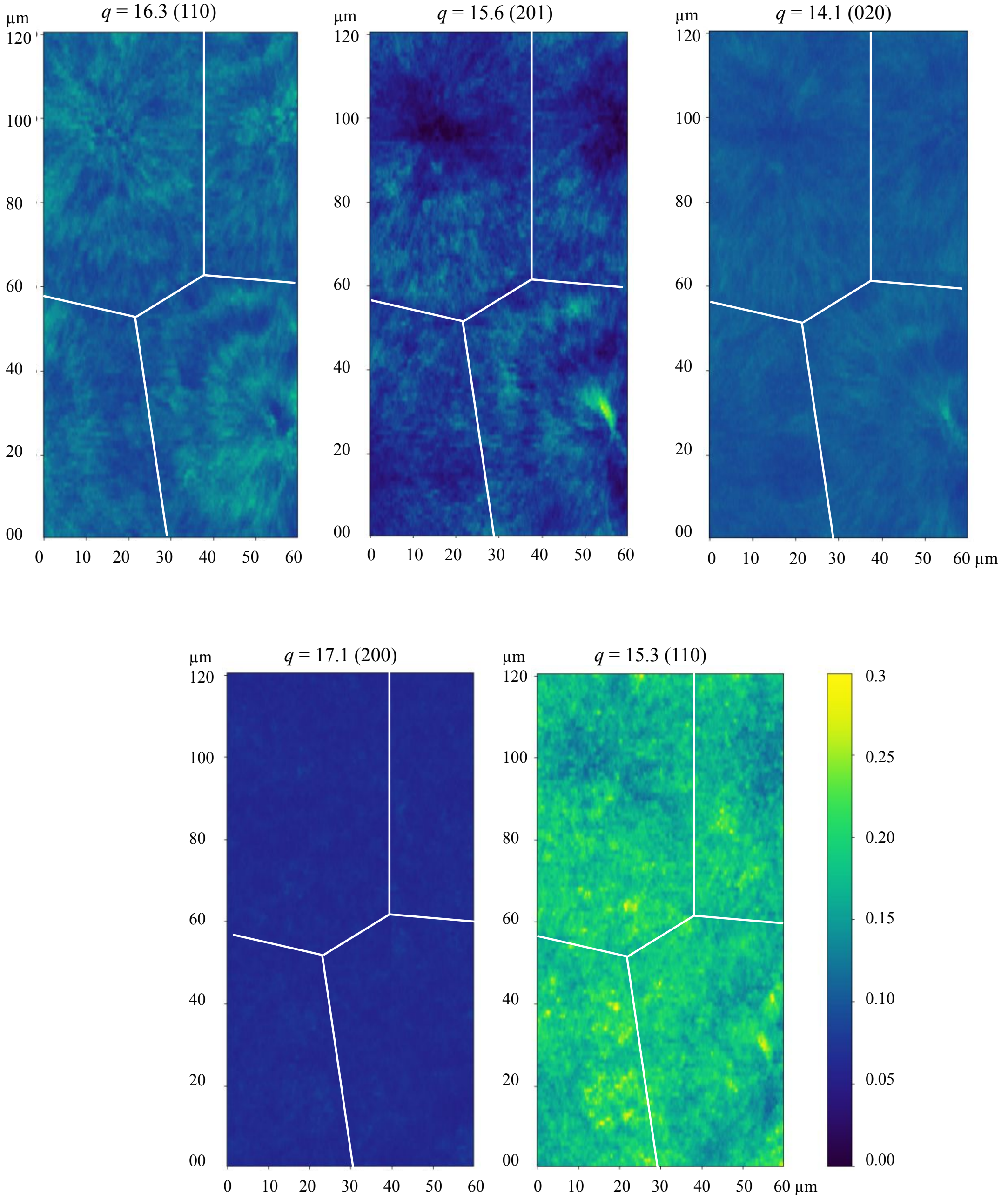

Figure S22. Intensity map of the 110$_{PBS}$ (a), 201$_{PBS}$ (b), 020$_{PBS}$ (c), 200$_{PCL}$ (d), 110$_{PCL}$ (e) reflections. The solid white lines are drawn to guide the eye, indicating the borders between spherulites where they impinged.

S2.8. Degradation studies, Hydrolytic and enzymatic degradation

Figure S23-24 and Table S4-5 show that the hydrolytic degradation of the samples progressed very slowly, resulting in only minimal weight loss over the full 9-week testing period. This limited mass reduction, less than 2%) indicates that under the applied conditions, erosion of both the amorphous and crystalline fractions is negligible, and the polymer matrix remains largely intact throughout the duration of the study.

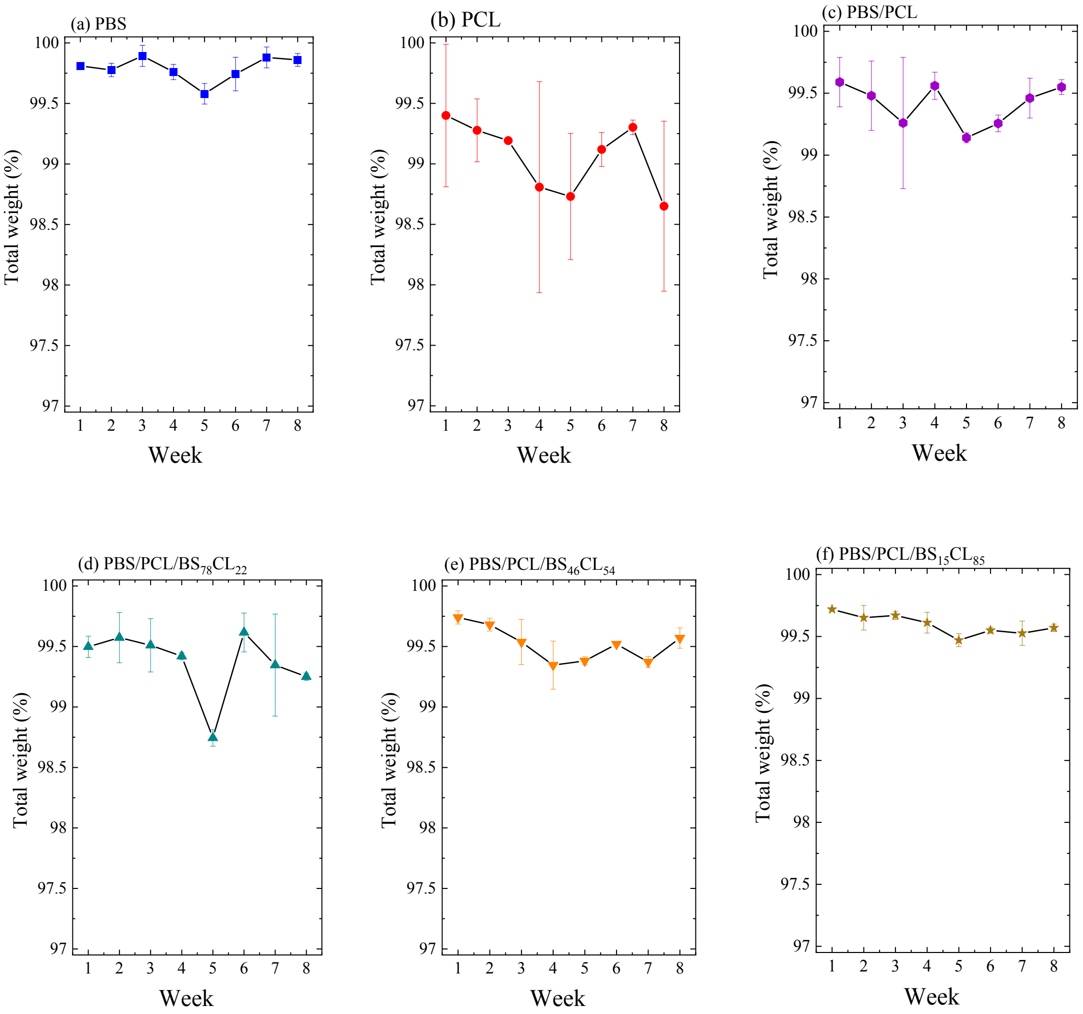

Figure S23. Representation of weight loss over time for (a) PBS, (b) PCL, (c) PBS/PCL, (d) PBS/PCL/$BS_{78}CL_{22}$, (e) PBS/PCL/$BS_{46}CL_{54}$, and (f) PBS/PCL/$BS_{15}CL_{85}$.

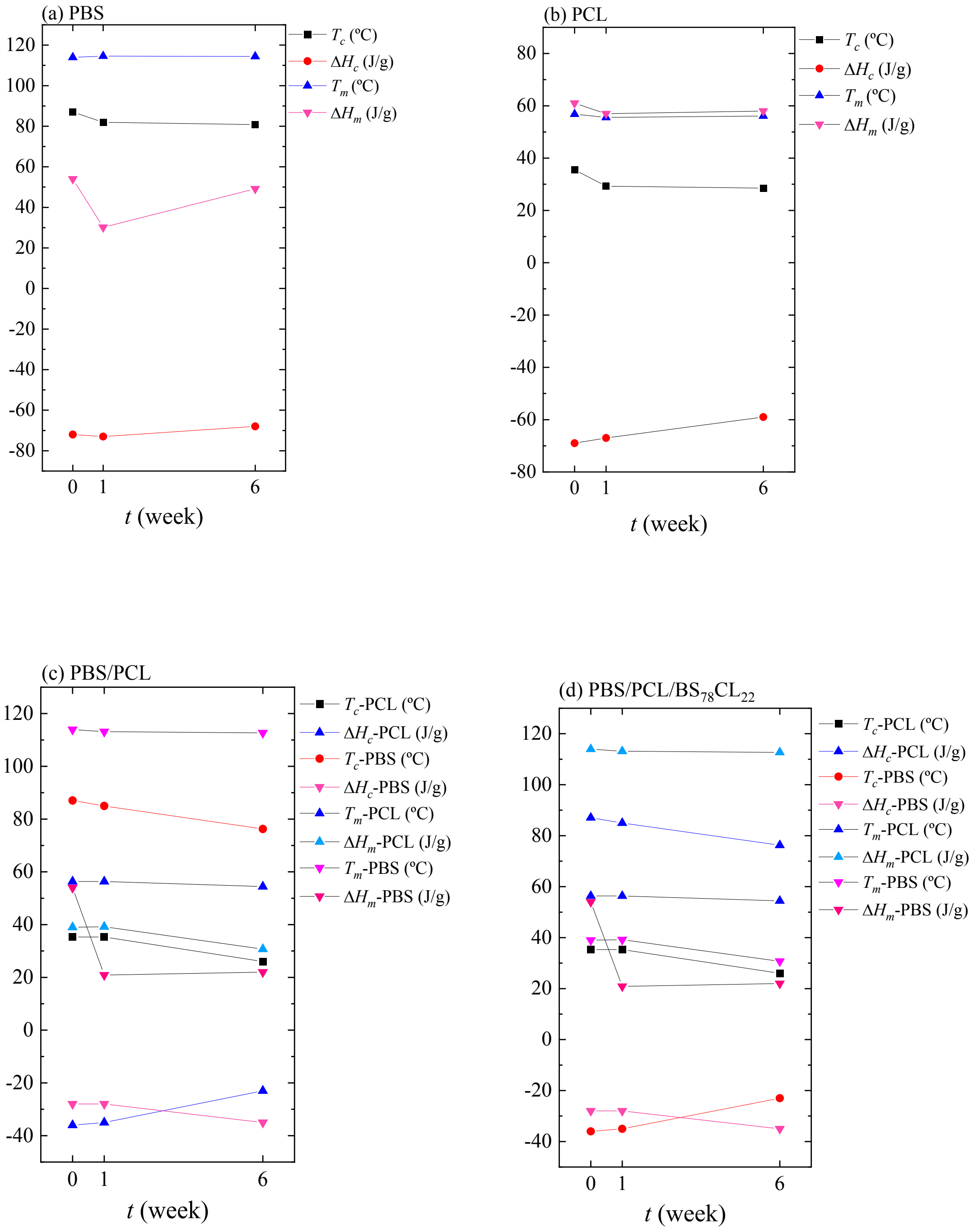

(a) PBS
T_c (ºC)
ΔH_c (J/g)
T_m (ºC)
ΔH_m (J/g)
t (week)
(b) PCL
T_c (ºC)
ΔH_c (J/g)
T_m (ºC)
ΔH_m (J/g)
t (week)
(c) PBS/PCL
T_c-PCL (ºC)
ΔH_c-PCL (J/g)
T_c-PBS (ºC)
ΔH_c-PBS (J/g)
T_m-PCL (ºC)
ΔH_m-PCL (J/g)
T_m-PBS (ºC)
ΔH_m-PBS (J/g)
t (week)
(d) PBS/PCL/$BS_{78}CL_{22}$
T_c-PCL (ºC)
ΔH_c-PCL (J/g)
T_c-PBS (ºC)
ΔH_c-PBS (J/g)
T_m-PCL (ºC)
ΔH_m-PCL (J/g)
T_m-PBS (ºC)
ΔH_m-PBS (J/g)
t (week)

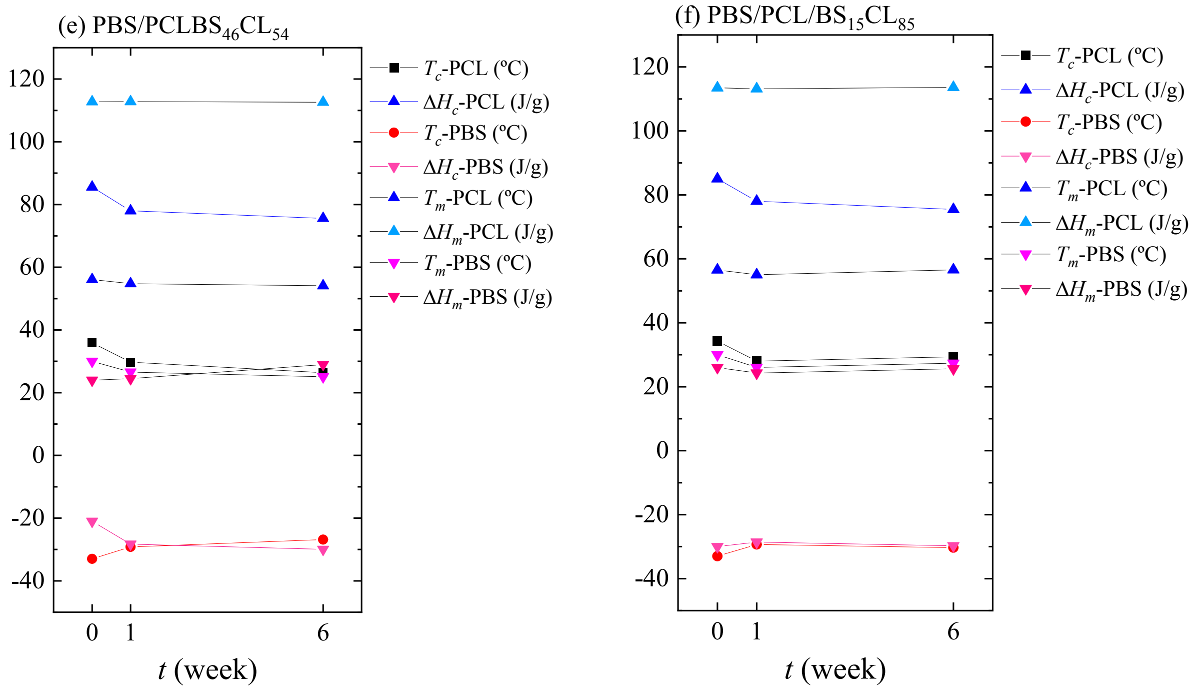

Figure S24. Values of the thermal transitions for each of the mixtures obtained by differential scanning calorimetry, (a) PBS, (b) PCL, (c) PBS/ PCL, (d) PBS/ PCL/ $BS_{78}CL_{22}$, (e) PBS /PCL /$BS_{46}CL_{54}$, and (f) PBS/ PCL/ $BS_{15}CL_{85}$.

Table S5. Summary of thermal transitions for the cooling and for the second heating runs at 10 ºC/min, after 1 week. (Hydrolytic degradation)

	PBS-rich phase										PCL-rich phase				
			cooling		Heating						cooling		Heating		
Sample	T_g (ºC) PBS	T_g (ºC) PCL	T_c (ºC)	ΔH_c (J/g)	T_{cc} (ºC)	ΔH_{cc} (J/g)	T_m (ºC)	ΔH_m (J/g)	X_c (%)		T_c (ºC)	ΔH_c (J/g)	T_m (ºC)	ΔH_m (J/g)	X_c (%)
PBS	-22.6	-	82.0	-73	107.8	-1	114.6	30	29		-	-	-	-	-
PBS/PCL	-25.8	-54.8	85.0	-28	105.2	-3	113.1	21	43		35.3	-35	56.4	39	56
PBS/PCL/$BS_{78}CL_{22}$	-21.7	-53.7	77.0	-33	101.9	-1	112.2	26	42		28.9	-29	54.0	24	34
PBS/PCL/$BS_{46}CL_{54}$	-31.7	-55.4	78.0	-28	103.4	-1	112.8	24	43		29.7	-29	54.8	27	35
PBS/PCL/$BS_{15}CL_{85}$	-25.5	-66.3	78.0	-29	10308	-1	113.2	24	45		28.0	-29	55.0	26	32
PCL	-	-51.9	-	-	-	-	-	-	-		29.3	-67	55.6	57	41

Table S6. Summary of thermal transitions for the cooling and for the second heating runs at 10 ºC/min, after 6 weeks. (Hydrolytic degradation)

	PBS-rich phase										PCL-rich phase				
			cooling		Heating						cooling		Heating		
Sample	T_g (ºC) PBS	T_g (ºC) PCL	T_c (ºC)	ΔH_c (J/g)	T_{cc} (ºC)	ΔH_{cc} (J/g)	T_m (ºC)	ΔH_m (J/g)	X_c (%)		T_c (ºC)	ΔH_c (J/g)	T_m (ºC)	ΔH_m (J/g)	X_c (%)
PBS	-30.5	-	80.8	-68	106.2	-5	114.4	49	50		-	-	-	-	-
PBS/PCL	-27.6	-47.7	76.3	-23	101.6	-1	112.7	22	21		26.0	-35	54.4	31	44
PBS/PCL/$BS_{78}CL_{22}$	-25.1	-53.4	75.1	-33	100.7	-2	112.9	31	31		28.4	-28	54.3	24	33
PBS/PCL/$BS_{46}CL_{54}$	-24.0	-46.0	75.6	-30	101.4	-1	112.6	29	29		26.4	-27	54.1	25	33
PBS/PCL/$BS_{15}CL_{85}$	-25.8	-45.5	75.4	-30	103.4	-1	113.6	26	25		29.3	-30	56.6	27	34
PCL	-	-51.9	-	-	-	-	-	-	-		28.5	-59	56.1	58	42

DSC analysis (first heating runs) tracked ΔH_m changes over time for enzymatic degradation. For PCL (Figure S25a), ΔH_m decreased, indicating enzymatic attack not only in the amorphous regions but also in crystalline regions. Complete loss of crystallinity ($\Delta H_m \approx 0$) occurred after 35 days in the blends, consistent with prior studies.[20-23] Initially, BS-rich copolymers accelerated PCL degradation, but at longer times, CL-rich copolymers led to greater overall weight loss.

For PBS (Figure S25b), ΔH_m increased, suggesting the preferential degradation of the amorphous regions and recrystallization during exposure to the degradation media. In blends, BS-rich copolymers ($BS_{46}CL_{54}$, $BS_{78}CL_{22}$) induced an early rise in $\mathit{\Delta} H_m$ that later stabilized, suggesting enhanced PBS crystallinity and reduced degradability. In contrast, CL-rich copolymers ($BS_{15}CL_{85}$) and the binary blend showed a continuous $\mathit{\Delta} H_m$ decreases, consistent with disrupted PCL crystallinity. Overall, blending with PCL promotes biodegradation in PBS-rich phases by facilitating enzyme access to amorphous domains.[16]

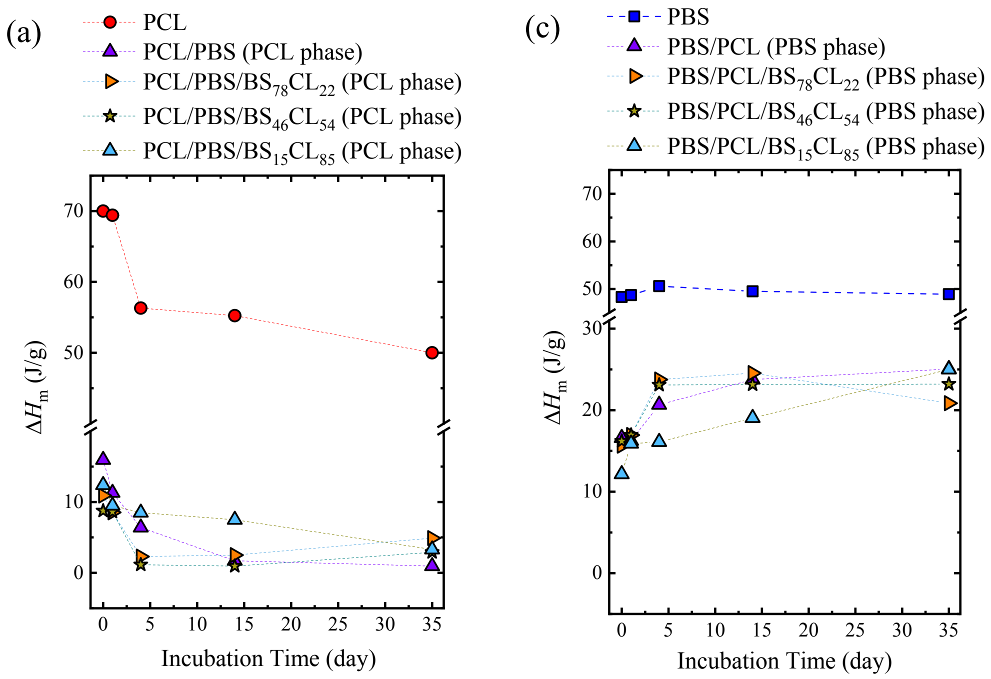

Figure S25. Enthalpy of fusion values versus incubation time for (a) PCL phase and (b) PBS phase of indicated ternary blend samples.

S3. Isodimorphism Definition

comonomers differ sufficiently to create a competition between exclusion from and partial inclusion into the crystal lattice during crystallization. In this case, although the major comonomer predominantly dictates the crystalline structure, a limited amount of the minor comonomer is always incorporated within the crystal unit cell. As a consequence, crystallization occurs across the entire composition range, and such systems are classified as isodimorphic copolymers. These materials form two distinct crystalline phases whose crystal structures closely resemble those of the corresponding parent homopolymers. When the melting temperature is plotted as a function of copolymer composition, a characteristic pseudoeutectic behavior is observed. On either side of the eutectic composition, crystallization yields a single crystalline phase enriched in the major component, with only limited inclusion of the minor comonomer. Notably, recent studies have shown that at the pseudoeutectic composition, the coexistence of two crystalline phases is possible, depending on the thermal history, with unit cell parameters analogous to those of the parent homopolymers.[2, 3, 19, 24-29]

References

(1) Candal, M. V.; Calafel, I.; Aranburu, N.; Fernandez, M.; Gerrica-Echevarria, G.; Santamaria, A.; Müller, A. J. Thermo-rheological effects on successful 3D printing of biodegradable polyesters. *Additive Manufacturing* **2020**, *36*, 101408.
(2) Safari, M.; Martinez de Ilarduya, A.; Mugica, A.; Zubitur, M.; Muñoz-Guerra, S.; Müller, A. J. Tuning the thermal properties and morphology of isodimorphic poly [(butylene succinate)-ran-(ε-caprolactone)] copolyesters by changing composition, molecular weight, and thermal history. *Macromolecules* **2018**, *51* (23), 9589-9601.
(3) Safari, M.; Otaegi, I.; Aramburu, N.; Guerrica-Echevarria, G.; de Ilarduya, A. M.; Sardon, H.; Müller, A. J. Synthesis, structure, crystallization and mechanical properties of isodimorphic PBS-ran-PCL copolyesters. *Polymers* **2021**, *13* (14), 2263.
(4) Woodruff, M. A.; Hutmacher, D. W. The return of a forgotten polymer—Polycaprolactone in the 21st century. *Progress in polymer science* **2010**, *35* (10), 1217-1256.
(5) Xu, J.; Guo, B. H. Poly (butylene succinate) and its copolymers: Research, development and industrialization. *Biotechnology journal* **2010**, *5* (11), 1149-1163.
(6) Colmenero, J.; Arbe, A. Segmental dynamics in miscible polymer blends: recent results and open questions. *Soft Matter* **2007**, *3* (12), 1474-1485.
(7) Lodge, T. P.; McLeish, T. C. Self-concentrations and effective glass transition temperatures in polymer blends. *Macromolecules* **2000**, *33* (14), 5278-5284.
(8) Peñas, M. I.; Pérez-Camargo, R. A.; Hernández, R.; Müller, A. J. A review on current strategies for the modulation of thermomechanical, barrier, and biodegradation properties of poly (butylene succinate)(PBS) and its random copolymers. *Polymers* **2022**, *14* (5), 1025.
(9) Righetti, M. C.; Di Lorenzo, M. L.; Cavallo, D.; Müller, A. J.; Gazzano, M. Structural evolution of poly (butylene succinate) crystals on heating with the formation of a dual lamellar population, as monitored by temperature-dependent WAXS/SAXS analysis. *Polymer* **2023**, *268*, 125711.

(10) John, J.; Mani, R.; Bhattacharya, M. Evaluation of compatibility and properties of biodegradable polyester blends. *Journal of Polymer Science Part A: Polymer Chemistry* **2002**, *40* (12), 2003-2014.
(11) Liu, Q.; Zhou, X.-M. Preparation of Poly (butylene succinate)/poly (ϵ-caprolactone) Blends Compatibilized With Poly (butylene succinate-co-ϵ-caprolactone) Copolymer. *Journal of Macromolecular Science, Part A* **2015**, *52* (8), 625-629.
(12) Wang, W.; Buzzi, S.; Fenni, S. E.; Carmeli, E.; Wang, B.; Liu, G.; Müller, A. J.; Cavallo, D. Surface nucleation of dispersed droplets in double semicrystalline immiscible blends with different matrices. *Macromolecular Chemistry and Physics* **2022**, *223* (21), 2200202.
(13) Mandala, Y. H.; Woo, E. M.; Ni'mah, H.; Nurkhamidah, S. Surface-relief and interior lamellar assembly in Janus-face spherulites of Poly (butylene succinate) crystallized with Poly (ethylene oxide). *Polymer* **2019**, *176*, 168-178.
(14) Bittiger, H.; Marchessault, R.; Niegisch, W. Crystal structure of poly-ε-caprolactone. *Structural Science* **1970**, *26* (12), 1923-1927.
(15) Hu, H.; Dorset, D. L. Crystal structure of poly (iε-caprolactone). *Macromolecules* **1990**, *23* (21), 4604-4607.
(16) Wang, X.; Zhou, J.; Li, L. Multiple melting behavior of poly (butylene succinate). *European Polymer Journal* **2007**, *43* (8), 3163-3170.
(17) Ichikawa, Y.; Kondo, H.; Igarashi, Y.; Noguchi, K.; Okuyama, K.; Washiyama, J. Crystal structures of α and β forms of poly (tetramethylene succinate). *Polymer* **2000**, *41* (12), 4719-4727.
(18) Verma, R.; Marand, H.; Hsiao, B. Morphological changes during secondary crystallization and subsequent melting in poly (ether ether ketone) as studied by real time small angle X-ray scattering. *Macromolecules* **1996**, *29* (24), 7767-7775.
(19) Safari, M.; Mugica, A.; Zubitur, M.; Martinez de Ilarduya, A.; Muñoz-Guerra, S.; Müller, A. J. Controlling the isothermal crystallization of isodimorphic PBS-ran-PCL random copolymers by varying composition and supercooling. *Polymers* **2019**, *12* (1), 17.
(20) Gigli, M.; Negroni, A.; Soccio, M.; Zanaroli, G.; Lotti, N.; Fava, F.; Munari, A. Enzymatic hydrolysis studies on novel eco-friendly aliphatic thiocopolyesters. *Polymer degradation and stability* **2013**, *98* (5), 934-942.
(21) Narancic, T.; Verstichel, S.; Reddy Chaganti, S.; Morales-Gamez, L.; Kenny, S. T.; De Wilde, B.; Babu Padamati, R.; O'Connor, K. E. Biodegradable plastic blends create new possibilities for end-of-life management of plastics but they are not a panacea for plastic pollution. *Environmental science & technology* **2018**, *52* (18), 10441-10452.
(22) Shaiju, P.; Dorian, B.-B.; Senthamaraikannan, R.; Padamati, R. B. Biodegradation of poly (butylene succinate)(PBS)/stearate modified magnesium-aluminium layered double hydroxide composites under marine conditions prepared via melt compounding. *Molecules* **2020**, *25* (23), 5766.
(23) Shirahama, H.; Kawaguchi, Y.; Aludin, M. S.; Yasuda, H. Synthesis and enzymatic degradation of high molecular weight aliphatic polyesters. *Journal of applied polymer science* **2001**, *80* (3), 340-347.
(24) Pérez-Camargo, R. A.; Arandia, I.; Safari, M.; Cavallo, D.; Lotti, N.; Soccio, M.; Müller, A. J. Crystallization of isodimorphic aliphatic random copolyesters: Pseudo-eutectic behavior and double-crystalline materials. *European Polymer Journal* **2018**, *101*, 233-247.
(25) Pérez-Camargo, R. A.; Safari, M.; Rodríguez, J. T.; Liao, Y.; Müller, A. J. Structure, morphology and crystallization of isodimorphic random copolymers: Copolyesters, copolycarbonates and copolyamides. *Polymer* **2023**, *287*, 126412.
(26) Safari, M.; Leon Boigues, L.; Shi, G.; Maiz, J.; Liu, G.; Wang, D.; Mijangos, C.; Müller, A. J. Effect of Nanoconfinement on the Isodimorphic Crystallization of Poly (butylene succinate-ran-caprolactone) Random Copolymers. *Macromolecules* **2020**, *53* (15), 6486-6497.
(27) Safari, M.; Maiz, J.; Shi, G.; Juanes, D.; Liu, G.; Wang, D.; Mijangos, C.; Alegría, Á.; Müller, A. J. How confinement affects the nucleation, crystallization, and dielectric relaxation of poly (butylene succinate) and poly (butylene adipate) infiltrated within nanoporous alumina templates. *Langmuir* **2019**, *35* (47), 15168-15179.

(28) Safari, M.; Pérez-Camargo, R. A.; Ballester-Bayarri, L.; Liu, G.; Mugica, A.; Zubitur, M.; Wang, D.; Müller, A. J. Biodegradable binary blends of poly (butylene succinate) or poly (ε-caprolactone) with poly (butylene succinate-ran-ε-caprolactone) copolymers: crystallization behavior. *Polymer* **2022**, *256*, 125206.
(29) Safari, M.; Torres, J.; Pérez-Camargo, R. A.; Martínez de Ilarduya, A.; Mugica, A.; Zubitur, M.; Sardon, H.; Liu, G.; Wang, D.; Müller, A. J. How the aliphatic glycol chain length determines the pseudoeutectic composition in biodegradable isodimorphic poly (alkylene succinate-ran-caprolactone) random copolyesters. *Biomacromolecules* **2024**, *25* (11), 7392-7409.
